# Performance of stenting in femoropopliteal disease: a systematic literature review and meta-analysis of proportions

**DOI:** 10.57264/cer-2025-0152

**Published:** 2026-03-10

**Authors:** Yann Gouëffic, Antonia Bosworth Smith, Fabian Distler, Anya Lissina, Juliane Hafermann, Rhodri Saunders, Andrew Holden, Sabine Steiner

**Affiliations:** 1 Vascular & Endovascular Center, Groupe Hospitalier Paris Saint Joseph, Paris, Paris, France; 2 Coreva Scientific GmbH & Co. KG, Koenigswinter, North Rhine-Westphalia, Germany; 3 Vascular Intervention Research Unit, Auckland Hospital, Auckland, Auckland, New Zealand; 4 Division of Angiology, Department of Medicine II, Medical University Vienna, Vienna, Vienna, Austria; 5 Helmholtz Institute for Metabolic, Obesity & Vascular Research of Helmholtz Center Munich at the University of Leipzig & University Hospital Leipzig, Leipzig, Saxony, Germany

**Keywords:** drug-eluting stent, femoropopliteal artery, paclitaxel, peripheral arterial disease, pooled rates, primary patency, target lesion revascularization

## Abstract

**Aim:**

To evaluate the performance of four stent types (BMS, bare metal stents; Eluvia, a polymer-based paclitaxel-eluting stent; Viabahn, a covered stent; and Zilver PTX, a polymer-free paclitaxel-coated stent) in femoropopliteal lesions at 12 and 24 months using a meta-analysis of proportions.

**Materials & methods:**

This systematic review (PROSPERO CRD42024528559) used PubMed to identify relevant single-arm and comparative studies (with ≥50 patients/study) published between 1 January 2009 and 1 July 2024. Data on patient/lesion characteristics and outcomes were extracted. Subgroup analyses were based on lesion length (<150 mm vs ≥150 mm) and study quality. A random-intercept logistic regression model was used to pool the data. The 95% CI around the pooled effect was calculated using Knapp–Hartung adjustments.

**Results:**

Data were extracted from 141 of the 870 screened studies, corresponding to 35,897 patients. The mean patient age was 70.9 (range: 63.3–80.0) years; 69.6% were male. The overall mean lesion length was 153.1 (range: 37–330) mm. Although all stent types performed well in the main analysis, Eluvia exhibited consistently high primary patency and low target lesion revascularization rates at both timepoints and across lesion lengths. Mortality rates for all stent types were stable for short lesions but more variable for long lesions.

**Conclusion:**

All stents used in short lesions performed well; however, Eluvia also demonstrated reliable performance in long lesions.

## Plain language summary: How well do stents work for blocked arteries between the hip & the knee: an overview of published evidence

**What is this article about?:**

Stenting is one of the treatments available for narrowed or blocked leg arteries. This study focused on four different stent types that are used in the artery that runs between the hip and the knee. We reviewed the available scientific literature on these stents and then combined the results from different studies to find out how often stenting is successful at keeping the artery open for 1 year and for 2 years. We also looked at the success of stents used in long or short sections of closed off artery.

**What were the results?:**

We combined the results from 141 studies that looked at 35,897 patients overall. The average performance and a range where 95% of the results would fall (the 95% CI) was calculated. Although all stents performed well, one stent infused with a drug had a range that did not overlap with the other stents, this could mean that this stent has a better performance.

**Why is this important?:**

The results of this analysis provide a summary of the performance of the different stent types which can help physicians understand the options available to them.

Peripheral arterial disease (PAD), a chronic vascular condition, is a substantial cause of morbidity, leading to mobility impairment, pain, limb amputation and death [1,2]. Treatment options include lifestyle modifications, pharmacological therapies and revascularization [3].

The femoropopliteal artery is frequently affected in PAD [4]. Endovascular interventions, including plain old balloon angioplasty (POBA) and stent placement, restore blood flow to stenotic or occluded femoropopliteal arteries. Drug-coated balloons are associated with higher patency rates than POBA, reducing the need for reintervention [5]. However, both strategies result in a frequent need for scaffolding to manage acute procedural complications [6]. The requirement for provisional stenting increases with lesion complexity and exceeds 40% in long lesions and chronic total occlusions (CTOs), highlighting the necessity for stent placement in these cases [7].

Despite the widespread use of stents in the treatment of femoropopliteal disease, clinicians face challenges in selecting the most appropriate stent due to limited comparative data. Numerous clinical trials have evaluated self-expanding nitinol stents, drug-eluting stents and covered stents in shorter to moderate-length lesions (reviewed in [8–10]). However, the evidence for longer, more complex real-world lesions is sparse. Additionally, there is a lack of randomized controlled trials (RCTs) directly comparing the effectiveness of different stent types in femoropopliteal lesions [11–13]. Thus, there is a pressing need to systematically evaluate the available evidence on stent performance in femoropopliteal disease. Understanding how different stent types perform across key clinical outcomes will be essential to improving treatment decisions and optimizing patient care.

The present study aimed to systematically review and synthesize the available literature on the four stent types commonly used to treat femoropopliteal disease: bare metal stents (BMS; specifically self-expanding nitinol stents); Eluvia™ (Boston Scientific, MA, USA), a polymer-based paclitaxel-eluting stent; Viabahn^®^ (W. L. Gore and Associates, AZ, USA), a covered stent; and Zilver^®^ PTX^®^ (Cook Medical, IN, USA), a polymer-free paclitaxel-coated stent.

## Materials & methods

The protocol was registered with PROSPERO (CRD42024528559). The findings were reported according to the PRISMA checklist (Supplementary Table 1) [14].

A systematic search of PubMed was performed on 30 January 2024 and updated on 8 July 2024. The final search included studies published between January 2009 and July 2024. The PICOS (population, intervention, comparison, outcome, study type) framework was adopted to guide the literature selection process. The full search strategy was published in the PROSPERO protocol. The identified studies were screened for duplicates and relevance using Laser AI (Evidence Prime Inc., Hamilton, Canada; https://www.laser.ai/) and PICO Portal (NY, USA; www.picoportal.org) and organized using the Citavi reference manager (Lumivero, CO, USA).

Studies meeting the following criteria were selected: evaluating the use of a vascular stent of interest in femoropopliteal arteries; reporting at least one primary outcome; patients with superficial femoral artery lesions or PAD; minimum of 50 participants per study. Abstract-only publications, commentaries, editorials, nonoriginal research articles (including reviews) and case reports were excluded.

Two reviewers independently screened titles and abstracts, followed by full-text assessment of eligible studies. Data were extracted using a standardized Excel (Microsoft, WA, USA) sheet. Disagreements regarding study eligibility or data extraction were resolved by a third reviewer. The extracted data included patient characteristics: age, sex, comorbidities, claudication, chronic limb-threatening ischemia (CLTI) and renal function; lesion characteristics: length, calcification, location and CTO; outcomes: primary patency, target lesion revascularization (TLR), all-cause mortality, major amputation, clinical improvement, stent fractures, walking distance and the ankle-brachial index. Outcomes were assessed at 12 and 24 months. The term ‘data points’ was used to indicate the number of study arms.

### Outcome definitions

The primary outcomes were primary patency and all TLR events. The secondary outcomes were mortality, major amputations, stent fractures, walking distance and clinical improvement. Primary patency was included as defined in the papers. Restenosis was not analyzed as an independent outcome but instead converted to primary patency rate by inverting the value. This was only done if a study did not report primary patency and if restenosis was defined using duplex ultrasound and angiographic assessments. If no TLR was reported, it was calculated from the inversion of freedom from TLR. If only clinically driven (CD)-TLR was reported, this outcome was used as the primary measure. Major amputations were defined as occurring above the ankle; if the study only reported freedom from major amputation, the inverse was calculated. Walking distance was assessed using the walking impairment questionnaire score. Clinical improvement was defined as improvement in the Rutherford classification from baseline.

### Statistical analysis & interpretation of results

Meta-analyses were conducted using R (version 4.4.2; R Core Team, 2024; R Foundation, Vienna, Austria), specifically the ‘metafor’ [15] and ‘meta’ [16] packages. A random-effects model was applied to account for the expected heterogeneity. Pooled proportions were calculated using a random-intercept logistic regression model. The Knapp–Hartung adjustment was applied to determine the 95% CI around the pooled effect.

The outcomes of a meta-analysis of proportion provides no direct comparison between interventions. What the outcome of each analysis shows, is an indication of the average effect and the underlying distribution of that effect for each stent type. As noted by Knol *et al.* [17], nonoverlapping 95% CIs from independent samples (as provided in this meta-analysis of proportions) could be considered statistically significant as the probability of it occurring at random is 0.0056, a factor of 10 smaller than the commonly used p-value cut-off of 0.05. These findings were validated in a study of clinical chemistry laboratory results, with the real data matching closely to the limits calculated by Knol *et al.* [18]. As such, we consider meta-analysis of proportion results that do not have overlapping 95% CIs to come from different underlying effect distributions. Superiority or inferiority is not implied, as potential confounders that impact on the underlying distribution cannot be discounted.

Due to the large number of studies and the resulting size of the forest plots, the pooled proportions are presented in condensed forest plots in the main body of the manuscript. The detailed forest plots are in the Supplementary Material.

### Risk of bias & additional analyses

Risk of bias was assessed using the Downs and Black Quality Appraisal tool [19]. This appraisal tool is suitable for all included study types. Scores were rated as ‘excellent’ (25–27), ‘good’ (20–24), ‘fair’ (15–19) or ‘poor’ (≤14).

Subgroup analyses were performed based on the mean lesion length reported in the study (short <150 mm vs long ≥150 mm). *Post hoc* sensitivity analyses were done based on the use of core laboratory adjudication by excluding studies that did not use this, and based on the risk of bias scores by excluding all studies rated ‘poor’ in Downs and Black Quality Appraisal [19].

## Results

### Study selection & characteristics

**Figure 1.**
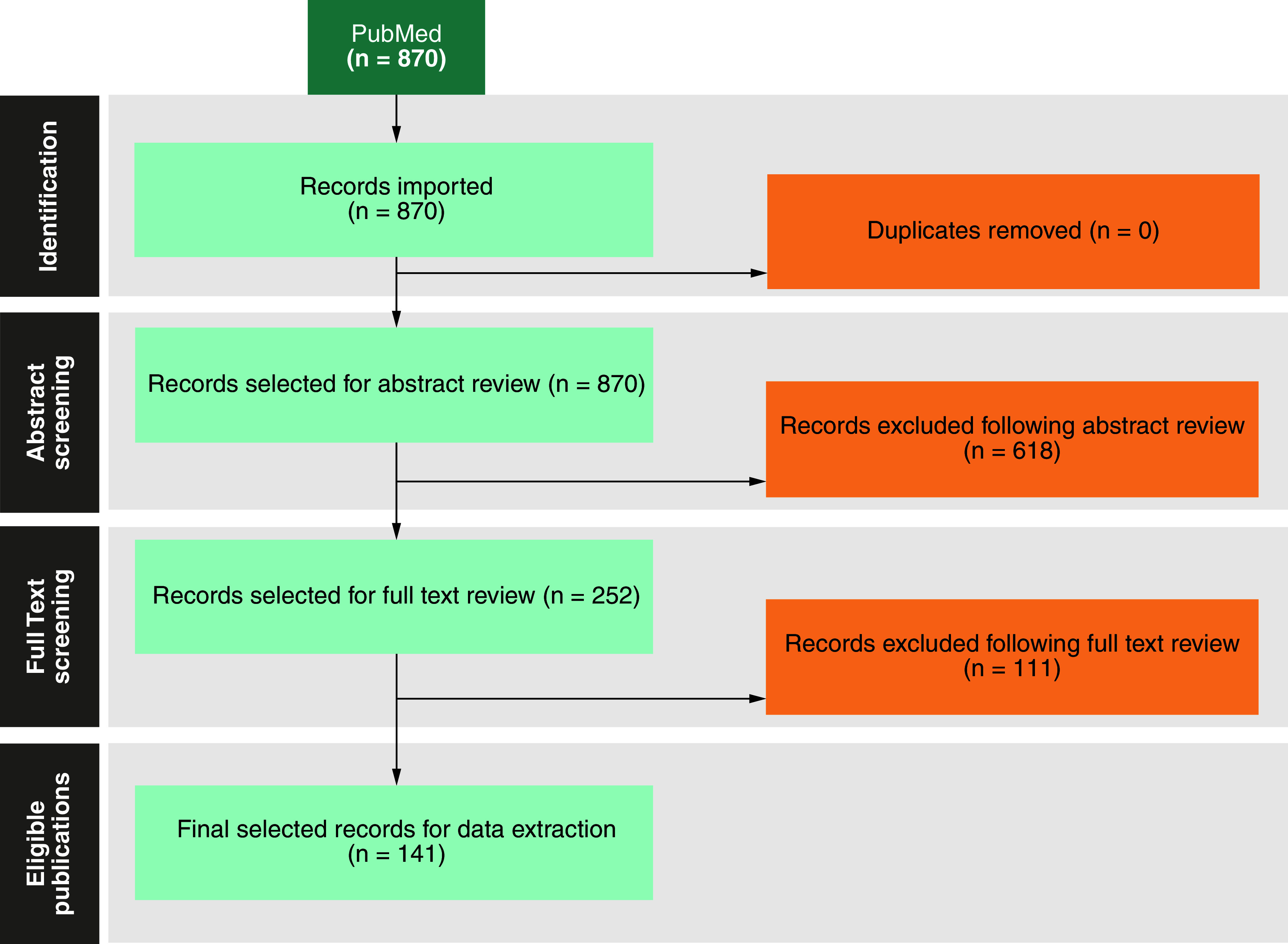
The PRISMA workflow diagram for the systematic literature review.

The study selection process is summarized in the PRISMA diagram (Figure 1). The initial search retrieved 870 unique articles, of which 252 were selected for full-text review. A total of 111 studies did not meet the inclusion criteria during full-text screening; consequently, 141 studies comprising 35,897 patients were selected for data extraction. 

The studies were prospective (25/141), nonrandomized prospective (47/141) and retrospective (69/141). Their patient and lesion characteristics are summarized in Table 1. The mean patient age was 70.9 (range: 63.3–80.0) years; 69.6% (range: 44.1–100.0%) of the patients were male. The most frequently reported comorbidities were hypertension (80.8% [range: 22.9–99.0%]) and diabetes mellitus (49.8% [range: 19.5–100.0%]). Although reduced kidney function and cardiovascular disease were also prevalent, they had broad definitions and were heterogeneously reported. The reported rate of CLTI was 35.2% (range: 0–100%). The overall mean lesion length was 153.1 mm (range: 37.0–330.0) (Eluvia: 159.4 mm [range: 70.8–230.0]; Zilver PTX: 159.6 mm [range: 64.2–330.0]; Viabahn: 229.7 mm [range: 173.0–290.0]; BMS: 140.5 mm [range: 37.0–330.0]) (Supplementary Table 2). The mean CTO rate was 55.7% (range: 16.4–100.0%).

**Table 1.** Summary of principal patient and lesion characteristics reported by the included studies.

Article	Stent type	Population	Lesion characteristics		Patient characteristics			Ref.
		Patients per intervention group (n)	Mean lesion length (mm)	CTO (%)	Mean age (years)	Male sex (%)	CLTI (%)	
Abi-Khalil *et al.* 2022	BMS	35	65.5	NR	70.9	77.1	NR	[20]
Abi-Khalil *et al.* 2022	Eluvia	27	72.6	NR	67.7	81.5	NR	[20]
AbuRahma *et al.* 2022	Zilver PTX	94	208	68	69.3	57	68	[21]
Armstrong *et al.* 2014	BMS	64	93	31	67	50	NR	[22]
Armstrong *et al.* 2014	BMS	84	257	63	70	56	NR	[22]
Armstrong *et al.* 2020	BMS	118	144.5	59.3	68.7	77.1	23.7	[23]
Astarcıoglu *et al.* 2017	BMS	53	330	100	69	79.2	0	[24]
Bausback *et al.* 2019^†^	Zilver PTX	75	155.5	52	69.5	76	16	[25]
Bertges *et al.* 2021	BMS	2113	230	56	71	58	69	[26]
Bianchini Massoni *et al.* 2023	Zilver PTX	203	107	75.4	73.5	66.5	75.9	[27]
Bisdas *et al.* 2018	Eluvia	62	200	79	71	63	58	[28]
Bosiers *et al.* 2009	BMS	151	96.4	40	67.8	73.5	NR	[29]
Bosiers *et al.* 2011	BMS	100	242	NR	70	66	29	[30]
Bosiers *et al.* 2015^†^	Viabahn	39	173	23.1	67.7	74.4	NR	[31]
Bosiers *et al.* 2023^†^	Zilver PTX	113	241.7	92	69.6	69	29.2	[32]
Bunte *et al.* 2018	BMS	250	77.3	23.6	67.7	61.6	NR	[33]
Chan *et al.* 2015	BMS	153	105.6	34	76.7	62.7	NR	[34]
Cheban *et al.* 2023^†^	Zilver PTX	30	220	100	63.3	83.3	NR	[35]
Dake *et al.* 2013	Zilver PTX	787	99.5	38.3	67.1	73.4	11	[36]
Dake *et al.* 2016^†^	Zilver PTX	236	66.4	32.8	67.9	65.7	8.9	[37]
Davaine *et al.* 2012	BMS	58	220	NR	71.4	72.4	59.7	[38]
Davaine *et al.* 2013	BMS	58	210	NR	70.5	73.2	62	[39]
Dearing *et al.* 2009	BMS	219	129	43	76.2	44.1	NR	[40]
Dick *et al.* 2009^†^	BMS	34	82	38	69	74	NR	[41]
Elmahdy *et al.* 2017	BMS	213	179	100	70.9	66.2	33.8	[42]
Falkowski *et al.* 2020^†^	Zilver PTX	126	93.8	NR	66.6	64	NR	[43]
Falkowski *et al.* 2020^†^	BMS	130	127.6	NR	64.9	63	NR	[43]
Fujihara *et al.* 2016	Zilver PTX	60	188.1	48.3	72.5	65	31.6	[44]
Fujihara *et al.* 2023	BMS	299	97.1	33.4	75	72.9	34.3	[45]
Gabrielli *et al.* 2015	BMS	30	80	100	NR	70	20	[46]
Gabrielli *et al.* 2015	BMS	41	80	100	NR	61	17	[46]
Garcia *et al.* 2015	BMS	264	78.1	25	68.7	63.6	NR	[47]
Geraghty *et al.* 2013^†^	Viabahn	72	190	61.1	69	62.5	NR	[48]
Golzar *et al.* 2020	Eluvia	50	162.8	32	68.2	64	NR	[49]
Gostev *et al.* 2022	BMS	143	250	NR	63.5	81.1	NR	[50]
Gostev *et al.* 2023	BMS	52	198	100	67	74.5	16.3	[51]
Gouëffic *et al.* 2020^†^	Zilver PTX	86	69	38	71	72	NR	[13]
Gouëffic *et al.* 2020^†^	BMS	85	76	35	68	73	NR	[13]
Gouëffic *et al.* 2022^†^	BMS	267	72.2	39.9	68.9	67.4	NR	[12]
Gouëffic *et al.* 2022^†^	Eluvia	508	75.6	42.3	68.9	71.5	NR	[12]
Gray *et al.* 2018^†^	Zilver PTX	156	81.8	30	67.8	67	NR	[11]
Gray *et al.* 2018^†^	Eluvia	309	86.5	31	68.5	66	NR	[11]
Gray *et al.* 2022	BMS	257	71	28	68	66.1	NR	[52]
Guo *et al.* 2015	BMS	53	314.8	NR	74.2	67.9	Nr	[53]
Guzzardi *et al.* 2021	BMS	99	150.4	41.9	74.5	69.7	64.4	[54]
Haine *et al.* 2019	Zilver PTX	77	116	71.6	72.3	64.1	36.2	[55]
Hendriks *et al.* 2020	BMS	117	71.4	30.6	69.4	70.9	NR	[56]
Horie *et al.* 2024a	BMS	204	155	42.2	75	75	NR	[57]
Horie *et al.* 2024a	Viabahn	137	265	67.9	77	65.7	NR	[57]
Horie *et al.* 2024b	BMS	177	37	59.7	77	60.5	31.6	[58]
Hu *et al.* 2011	BMS	138	203.5	100	74.35	71.01	NR	[59]
Ichihashi *et al.* 2019	Zilver PTX	220	164	45.2	73.1	79.5	26.5	[60]
Ichihashi *et al.* 2022	Eluvia	211	230	64	75	72.5	NR	[61]
Ichihashi *et al.* 2022	Viabahn	293	242	77.5	74	78.2	NR	[61]
Iida *et al.* 2009	BMS	126	83	17	72	73	26	[62]
Iida *et al.* 2011a	BMS	861	152	54	72	70	26	[63]
Iida *et al.* 2011b	BMS	585	142	53	72	72	23	[64]
Iida *et al.* 2015	Zilver PTX	690	170	45	73.6	71	32	[65]
Iida *et al.* 2019^†^	BMS	52	92	29	74	71	NR	[66]
Iida *et al.* 2024	Eluvia	1097	186	53.2	75	69.4	34.8	[67]
Ito *et al.* 2021a	BMS	427	NR	NR	70	69.8	43	[68]
Ito *et al.* 2021a	BMS	157	NR	NR	76	62.4	46.8	[68]
Ito *et al.* 2021b	BMS	701	141	58.2	74	70.2	27.2	[69]
Jeon-Slaughter *et al.* 2018	BMS	784	156.5	63.65	64.09	82.65	22.83	[70]
Jeon-Slaughter *et al.* 2018	Zilver PTX	174	162.5	63.79	64.67	80.46	22.99	[70]
Kang *et al.* 2016	Zilver PTX	63	218.9	69.8	66.3	57.1	NR	[71]
Karashima *et al.* 2021	BMS	453	180	54	74	70	37	[72]
Karpenko *et al.* 2022^†^	BMS	35	229.2	65.7	65	71.43	NR	[73]
Katsuki *et al.* 2019	BMS	1250	143	40.3	73	69.8	NR	[74]
Katsuki *et al.* 2019	Zilver PTX	285	146	40.7	73	74.7	NR	[74]
Katsuki *et al.* 2020	Viabahn	53	217.5	83	77.7	72	NR	[75]
Kawamura *et al.* 2009	BMS	80	NR	NR	65	55	NR	[76]
Kichikawa *et al.* 2019	Zilver PTX	905	146	41.5	73.5	70.3	21.4	[77]
Kim *et al.* 2024	Eluvia	104	168.3	57.7	68.2	82.7	30.1	[78]
Ko *et al.* 2019^†^	BMS	66	238	87.9	70.3	81.8	30.3	[79]
Ko *et al.* 2019^†^	BMS	59	245	89.8	70	89.8	18.6	[79]
Kum *et al.* 2021	Eluvia	NR^‡^	201	NR	NR	NR	NR	[80]
Labed *et al.* 2021	BMS	64	295	85.9	80	62.5	76.6	[81]
Laird *et al.* 2012^†^	BMS	134	70	17	68	70.9	NR	[82]
Laird *et al.* 2014	BMS	196	60.7	29.9	68.7	63.3	NR	[83]
Laird *et al.* 2018^†^	BMS	197	107.6	42.1	66.7	71.6	NR	[84]
Laird *et al.* 2018^†^	BMS	70	117.9	37.1	67.9	70	NR	[84]
Lammer *et al.* 2013^†^	BMS	69	173.2	70	69.44	75	NR	[85]
Lammer *et al.* 2013^†^	Viabahn	72	189.8	79	68.85	67	NR	[85]
Lammer *et al.* 2015^†^	BMS	69	173.2	70	69.44	75	NR	[86]
Lammer *et al.* 2015^†^	Viabahn	72	189.8	79	68.85	67	NR	[86]
Lee *et al.* 2021	Zilver PTX	93	194.8	77.5	70.8	77.5	50	[87]
Leopardi *et al.* 2014	Zilver PTX	69	130.7	NR	70.4	81.1	18.8	[88]
Lichtenberg *et al.* 2014	BMS	118	111.5	56.7	71.9	54.2	NR	[89]
Lichtenberg *et al.* 2019	BMS	60	81.6	16.4	70.3	63.3	15.8	[90]
Liistro *et al.* 2019^†^	Zilver PTX	96	140.7	64	74.2	76	59.4	[91]
Loureiro *et al.* 2024	BMS	79	NR	NR	66.9	72.2	NR	[92]
Low *et al.* 2022	BMS	62	150	NR	76	69	NR	[93]
Ma *et al.* 2022	BMS	157	187	79.4	71.9	69.4	52.86	[94]
Marples *et al.* 2022	Viabahn	36	254.7	53	76	55	NR	[95]
Matsumi *et al.* 2016a	BMS	68	244	100	72.5	70.6	20.8	[96]
Matsumi *et al.* 2016b	BMS	107	114.3	31.4	70.4	73.8	33.5	[97]
Matsumi *et al.* 2016b	BMS	325	117.3	42.5	74.2	74.5	7.2	[97]
Matsumura *et al.* 2013	BMS	287	89.1	48.1	67.7	66.2	NR	[98]
McQuade *et al.* 2010^†^	Viabahn	40	NR	NR	72	80	NR	[99]
Meng *et al.* 2018	Zilver PTX	24	105.4	NR	70.92	79.2	83.3	[100]
Meng *et al.* 2018	BMS	70	107.9	NR	70.86	74.3	97.1	[100]
Miura *et al.* 2018^†^	BMS	85	96	36.5	73.4	64.7	NR	[101]
Miura *et al.* 2018^†^	Zilver PTX	85	110.5	50.6	73.1	70.6	NR	[101]
Montero-Baker *et al.* 2016	BMS	147	184.5	53	72.2	51	0.7	[102]
Mori *et al.* 2017	BMS	279	156	56	73.1	67	33	[103]
Mori *et al.* 2017	Zilver PTX	27	NR	NR	NR	72	15	[103]
Müller-Hülsbeck *et al.* 2016	Eluvia	57	70.8	46	69.3	82	NR	[104]
Müller-Hülsbeck *et al.* 2017	Eluvia	57	70.8	46	69.3	82	NR	[105]
Myint *et al.* 2016	BMS	97	151.5	43	75.3	70.10309	19	[106]
Nakamura *et al.* 2018	BMS	74	80.7	20.8	72.8	75.7	0	[107]
Nakao *et al.* 2024	Eluvia	173	201	41	76	66	59	[108]
Nanto *et al.* 2015	BMS	1476	NR	NR	73	68.09	29.67	[109]
Oberto *et al.* 2017	Zilver PTX	67	104	46.3	70.1	79	NR	[110]
Okuno *et al.* 2019	BMS	260	NR	NR	73.6	66.1	39.4	[111]
Palena *et al.* 2024	BMS	92	261	100	73.2	88	100	[112]
Park *et al.* 2022^†^	Zilver PTX	48	238.6	72.9	71.1	79.2	22.9	[113]
Park *et al.* 2022^†^	Zilver PTX	55	245.1	85.2	70.8	85.5	31.5	[113]
Phair *et al.* 2020a	Zilver PTX	56	64.15	41.1	70.5	60.7	NR	[114]
Phair *et al.* 2020b	Zilver PTX	97	115	41.2	68.2	56.7	NR	[115]
Phillips *et al.* 2018	Zilver PTX	41	139	34	69.7	71	NR	[116]
Phillips *et al.* 2018	Zilver PTX	48	330	83	67.9	54	NR	[116]
Powell *et al.* 2017	BMS	299	93.2	44.7	67.4	74.2	NR	[117]
Rammos *et al.* 2024	BMS	121	125.8	64	72.2	61.2	100	[118]
Rammos *et al.* 2024	BMS	381	126.2	54.4	69.4	66.9	0	[118]
Rastan *et al.* 2013^†^	BMS	119	41.3	32.9	72	63.9	NR	[119]
Salamaga *et al.* 2023	BMS	77	152.8	66	71	75	57	[120]
Saratzis *et al.* 2019	BMS	136	150	78	76	63	NR	[121]
Saxon *et al.* 2013	Viabahn	113	190	56	67	61.1	NR	[122]
Scheinert *et al.* 2011	BMS	107	90.2	30.8	68.9	71.9	16.8	[123]
Scheinert *et al.* 2013	BMS	101	58.4	47.5	73.1	52.5	NR	[124]
Schulte *et al.* 2012	BMS	744	63.9	37.6	68.6	66.7	NR	[125]
Shehada *et al.* 2022	Eluvia	75	140	71	72	59	27	[126]
Shehada *et al.* 2022	BMS	124	160	84	74	59	16	[126]
Shibata *et al.* 2023	Eluvia	98	160	56.7	76.9	68.3	28.8	[127]
Shibata *et al.* 2023	Zilver PTX	86	185.7	53.1	75.3	71.9	32.3	[127]
Soga *et al.* 2010	BMS	511	150.5	50	72	71	24	[128]
Soga *et al.* 2011	BMS	807	145.3	54.4	72.3	68.9	25.8	[129]
Stavroulakis *et al.* 2016	BMS	89	116	55	71	62	NR	[130]
Stavroulakis *et al.* 2021	Eluvia	130	194	74	71	63	NR	[131]
Steiner *et al.* 2016	BMS	470	125	53	70.5	67	19	[132]
Steiner *et al.* 2016	BMS	432	148	58	67.6	70	25	[132]
Stern *et al.* 2021	Zilver PTX	64	201.5	NR	70.46	71.9	29.7	[133]
Sullivan *et al.* 2021	BMS	271	81.2	30	68.4	66.4	NR	[134]
Suzuki *et al.* 2016	BMS	240	120	37	72	65	35	[135]
Suzuki *et al.* 2016	BMS	1265	140	55	73	70	32	[135]
Tan *et al.* 2022a	Zilver PTX	65	171	53.8	73.8	72.3	NR	[136]
Tan *et al.* 2022b	BMS	250	200	58	75	60	45	[137]
Teymen *et al.* 2018	BMS	49	147.02	49	64.35	71.4	NR	[138]
Torsello *et al.* 2024	Eluvia	130	194	74	71	63	31	[139]
Treitl *et al.* 2017	BMS	85	41.7	NR	70.5	58.8	NR	[140]
Treitl *et al.* 2017	BMS	67	51.4	NR	72.5	64.2	NR	[140]
Treitl *et al.* 2017	BMS	70	52.8	NR	74.7	70	NR	[140]
Tsujimura *et al.* 2021	Zilver PTX	271	220	59	74	67.2	NR	[141]
Tsujimura *et al.* 2021	Viabahn	174	255	69	75	74.1	NR	[141]
Van Meirvenne *et al.* 2023	BMS	128	143	NR	75.5	53	45	[142]
Vartanian *et al.* 2013	BMS	NR^‡^	NR	30	72	76	52	[143]
Vartanian *et al.* 2013	Viabahn	NR^‡^	NR	59	68.5	75	33	[143]
Vent *et al.* 2017	BMS	58	220	NR	71.4	72.4	59.7	[144]
Vent *et al.* 2017	Zilver PTX	45	252	NR	67	71.1	46.7	[144]
Watanabe *et al.* 2018	BMS	104	263	100	72.8	72.4	26	[145]
Watanabe *et al.* 2018	BMS	95	269	100	74.4	65.8	48.6	[145]
Werner *et al.* 2013	BMS	100	69.5	29	67.6	76	NR	[146]
Werner *et al.* 2014	BMS	470	126.4	52.6	70.5	67.2	18.2	[147]
Wittig *et al.* 2024^†^	Eluvia	60	191.7	78.3	66.5	81.7	NR	[148]
Yang *et al.* 2022	BMS	246	147.7	81.2	73.2	60.2	87.3	[149]
Ye *et al.* 2022	Zilver PTX	178	79	50	67.4	78.8	NR	[150]
Ye *et al.* 2023	Viabahn	65	290	71.6	73.1	77.6	61.2	[151]
Yokoi *et al.* 2016	Zilver PTX	907	147	41.6	73.5	70.3	21.5	[152]
Yoshioka *et al.* 2023	Eluvia	65	174.7	61.5	75.3	72.3	NR	[153]
Zamani *et al.* 2021	Zilver PTX	57	200	NR	65.8	93	67	[154]
Zamani *et al.* 2021	Viabahn	74	260	NR	64	100	31	[154]
Zamani *et al.* 2021	BMS	95	280	NR	65.4	99	39	[154]
Zeller *et al.* 2014	Zilver PTX	97	195	62.9	68.2	63.9	NR	[155]
Zeller *et al.* 2016^†^	BMS	26	63.3	46	66.8	65	0	[156]
Zeller *et al.* 2016^†^	BMS	50	65.8	44	68	66	4	[156]
Zhao *et al.* 2021	BMS	39	187.8	61.9	66.5	61.5	23.6	[157]

† Prospective randomized studies included in this analysis.

‡ Number of limbs reported instead of the number of patients.

In some cases, more than one arm in a study used the same stent type but different brands. A more detailed table of patient and lesion characteristics is included in the Supplementary Data (Supplementary Table 3).

BMS: Bare metal stent (specifically self-expanding nitinol stent); CLTI: Chronic limb-threatening ischemia; CTO: Chronic total occlusion; NR: Not reported.

Overall, 38 (27.0%) included studies reported using core laboratory adjudication. According to the Downs and Black Quality Appraisal tool [19] (Supplementary Table 4), 94/141 (66.7%) studies had a rating of ‘fair’ or above (‘excellent’: n = 2; ‘good’: n = 18; ‘fair’: n = 74; ‘poor’: n = 47).

### Synthesis of results for the primary outcomes

**Figure 2.**
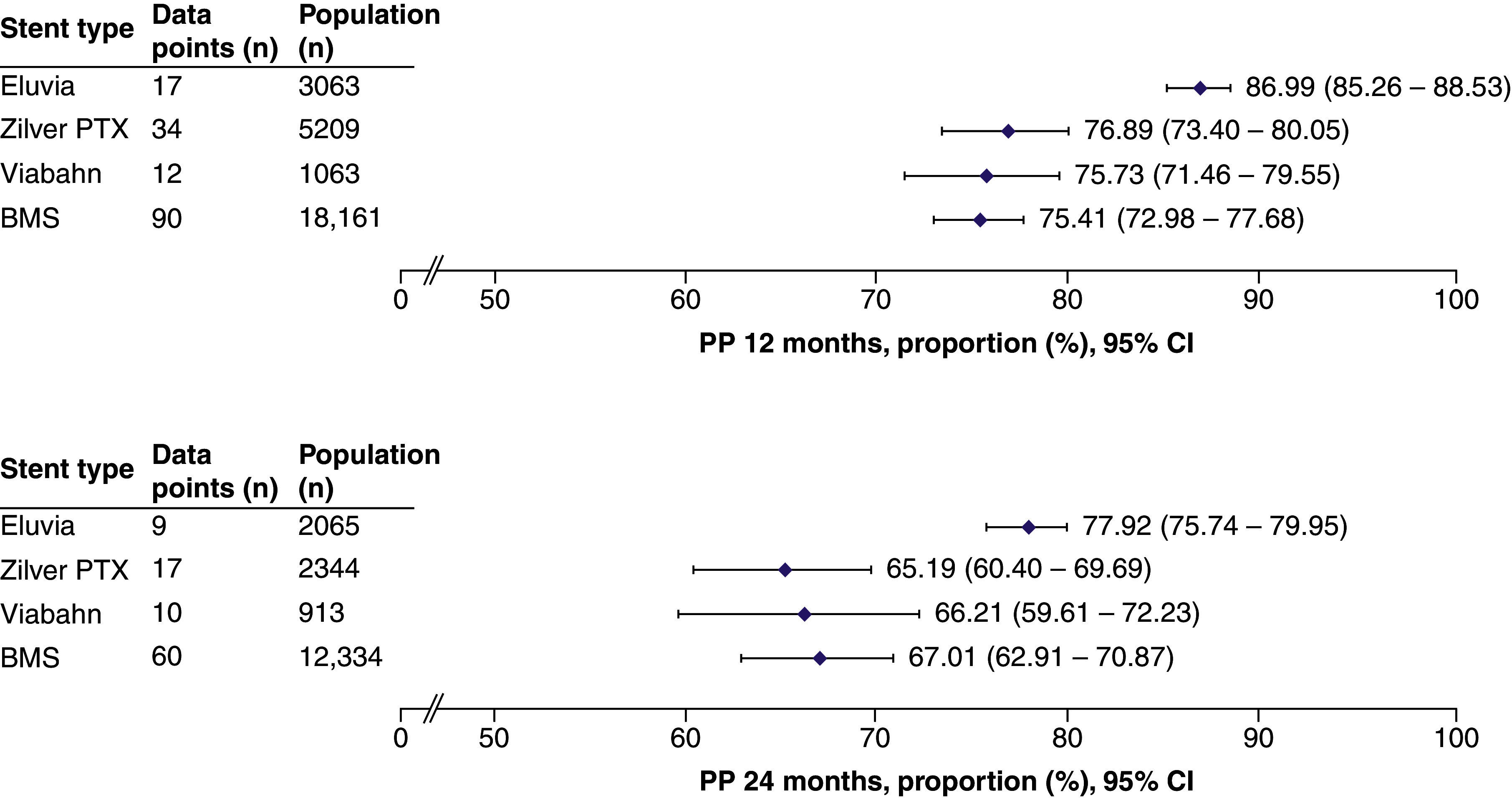
Summary forest plots showing primary patency at 12 and 24 months post stenting. The pooled estimates of primary patency (and the corresponding 95% CI values) are shown for each stent type at 12 months (top panel) or 24 months (bottom panel). The full forest plot for each stent type at each time point can be found in Supplementary Figures 1–8. These data were extracted from the following references: Eluvia [11,12,20,28,49,61,67,78,80,104,105,108,126,127,131,139,148,153]; Zilver PTX [11,13,21,25,27,35,37,44,60,65,71,77,87,88,91,100,101,103,110,113–116,127,133,141,144,150,152,154,155,158]; Viabahn [48,57,61,75,85,86,95,99,122,141,151,154,158]; BMS [12,13,20,22,24,29,30,33,34,38–42,45–47,50,51,53,54,56–59,62–64,66,69,72,73,76,79,81–86,89,90,92–94,96–98,100–103,106,107,109,111,112,117,119–121,123–126,128–130,132,134,135,137,140,142,144–147,149,154,156,157]. BMS: Bare metal stent; CI: Confidence interval; PP: Primary patency.

Primary patency was reported in 129/141 (91%) studies. The total patient population across all stent types included 27,496 patients (153 data points) at 12 months and 17,656 patients (96 data points) at 24 months. Eluvia exhibited primary patency rates with the lower bound of its 95% CI exceeding the upper bound of that of the other stent types at both timepoints (Figure 2).

**Figure 3.**
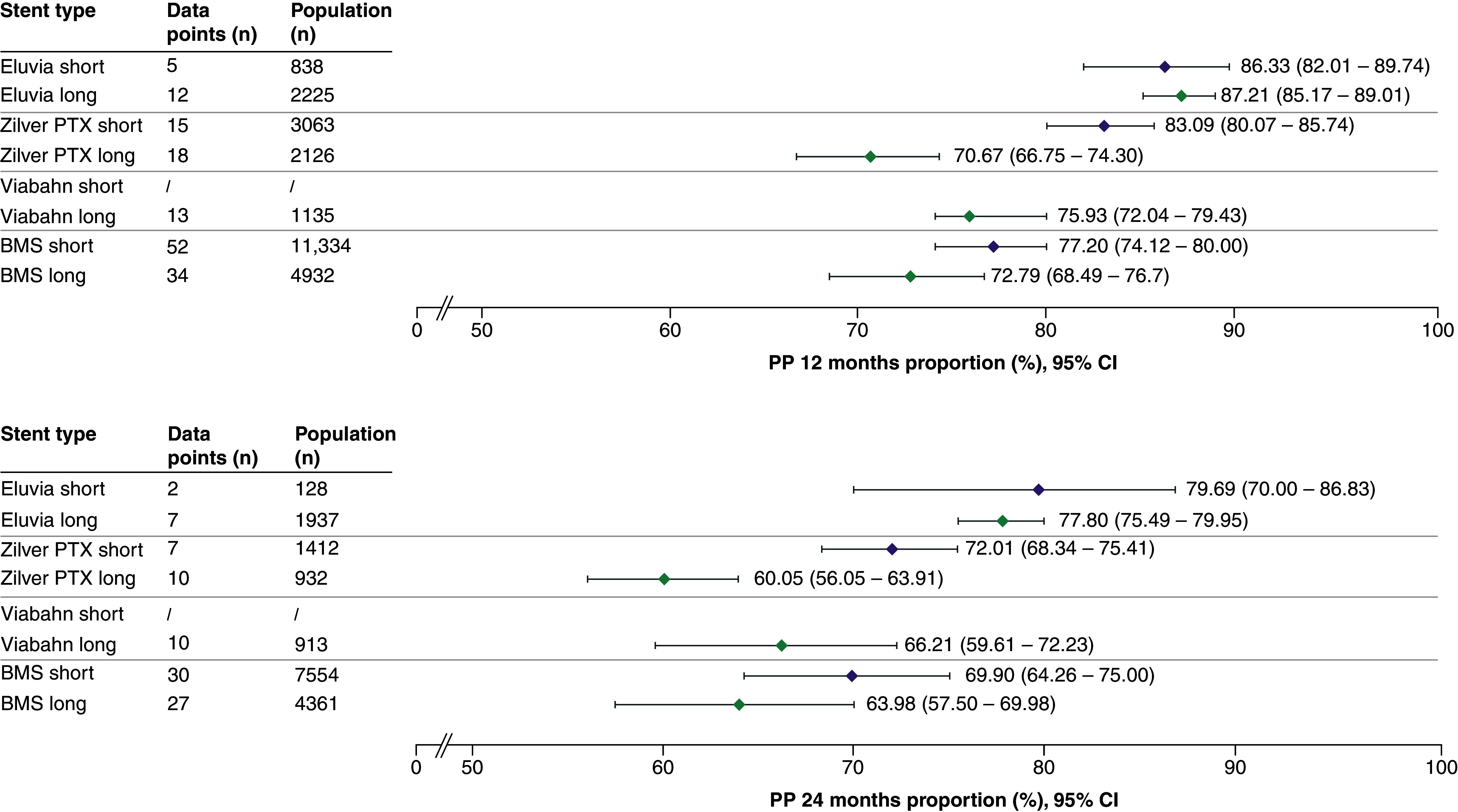
Summary forest plots showing primary patency for short and long lesions at 12 and 24 months post stenting. The pooled estimates of primary patency (and the corresponding 95% CI values) are shown for each stent type in short (orange) or long (blue) lesions at 12 months (top panel) or 24 months (bottom panel). The full forest plot for each stent type at each time point can be found in Supplementary Figures 9–14. BMS: Bare metal stent; CI: Confidence interval; PP: Primary patency.

In the subgroup analysis, Eluvia achieved consistently high primary patency in both short and long lesions at 12 and 24 months (Figure 3). Zilver PTX performed better in short than in long lesions at both timepoints. Viabahn was not used in short lesions; however, it achieved a primary patency of 75.93 (95% CI: 72.04–79.43) and 66.21 (95% CI: 59.61–72.23) at 24 months in long lesions. The performance of BMSs in both short lesions and long lesions declined at 24 versus 12 months.

**Figure 4.**
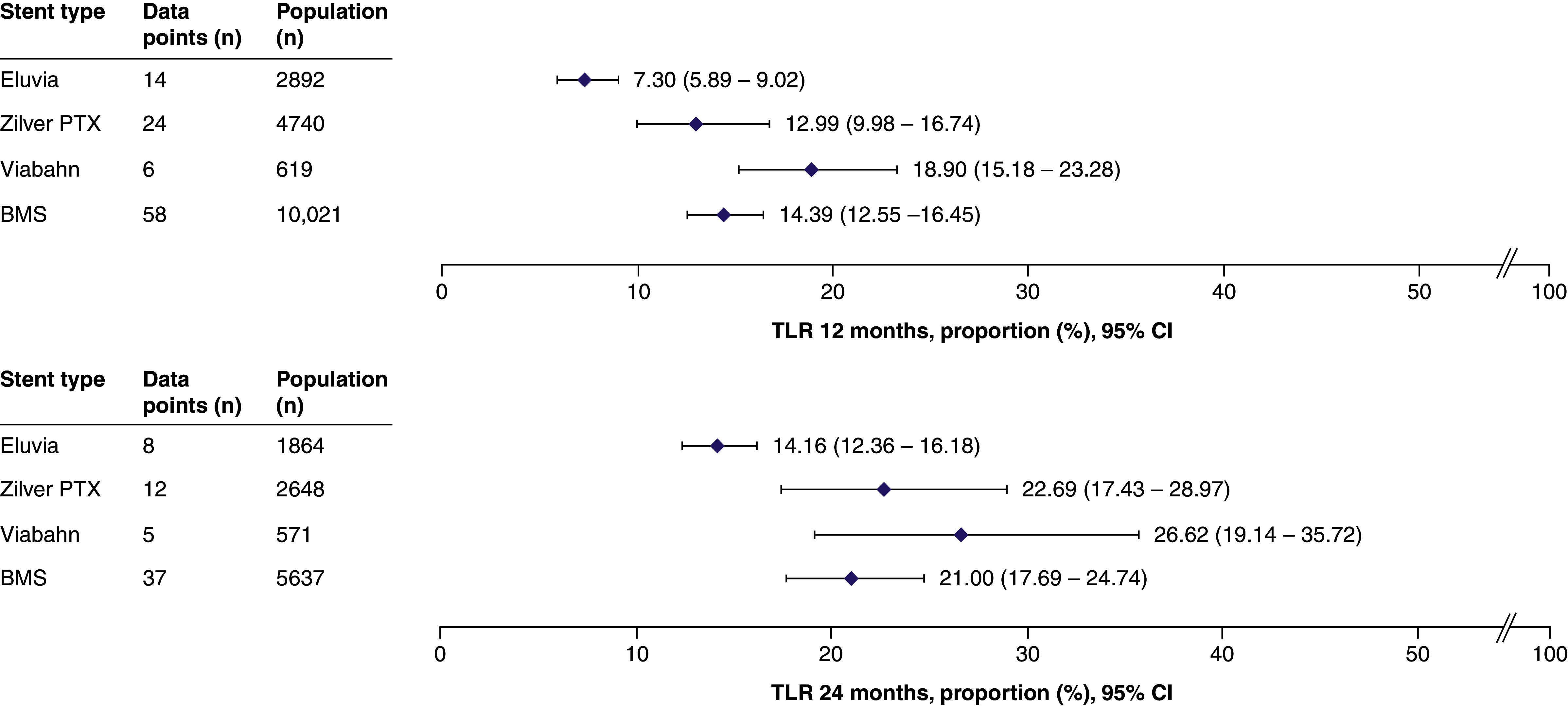
Summary forest plots showing target lesion revascularization at 12 and 24 months post stenting. The pooled estimates of TLR (and the corresponding 95% CI values) are shown for each stent type at 12 months (top panel) or 24 months (bottom panel). The full forest plot for each stent type at each time point can be found in Supplementary Figures 30–37. These data were extracted from the following references: Eluvia [8,11,28,49,61,67,78,105,126,127,131,139,148,153]; Zilver PTX [11,13,25,27,35–37,44,55,60,70,77,87,91,100,101,113,114,127,144,150,152,154,155,158]; Viabahn [31,57,58,61,85,151,154]; BMS [12,13,23,24,29,30,33,38,45,47,51,52,54,56–58,66,68–70,76,81–86,89,90,94,97,98,100,101,106,107,112,117–119,121,125,126,130,134,140,142,144–147,154,156,157]. BMS: Bare metal stent; CI: Confidence interval; TLR: Target lesion revascularization.

TLR was reported in 85/141 (60%) studies. The total patient population included 18,272 patients (102 data points) at 12 months and 10,720 patients (62 data points) at 24 months. Eluvia exhibited the lowest TLR rates among the stent types at both timepoints, with the upper limit of its 95% CI staying below the lower limit of the 95% CI of the other stents (Figure 4).

**Figure 5.**
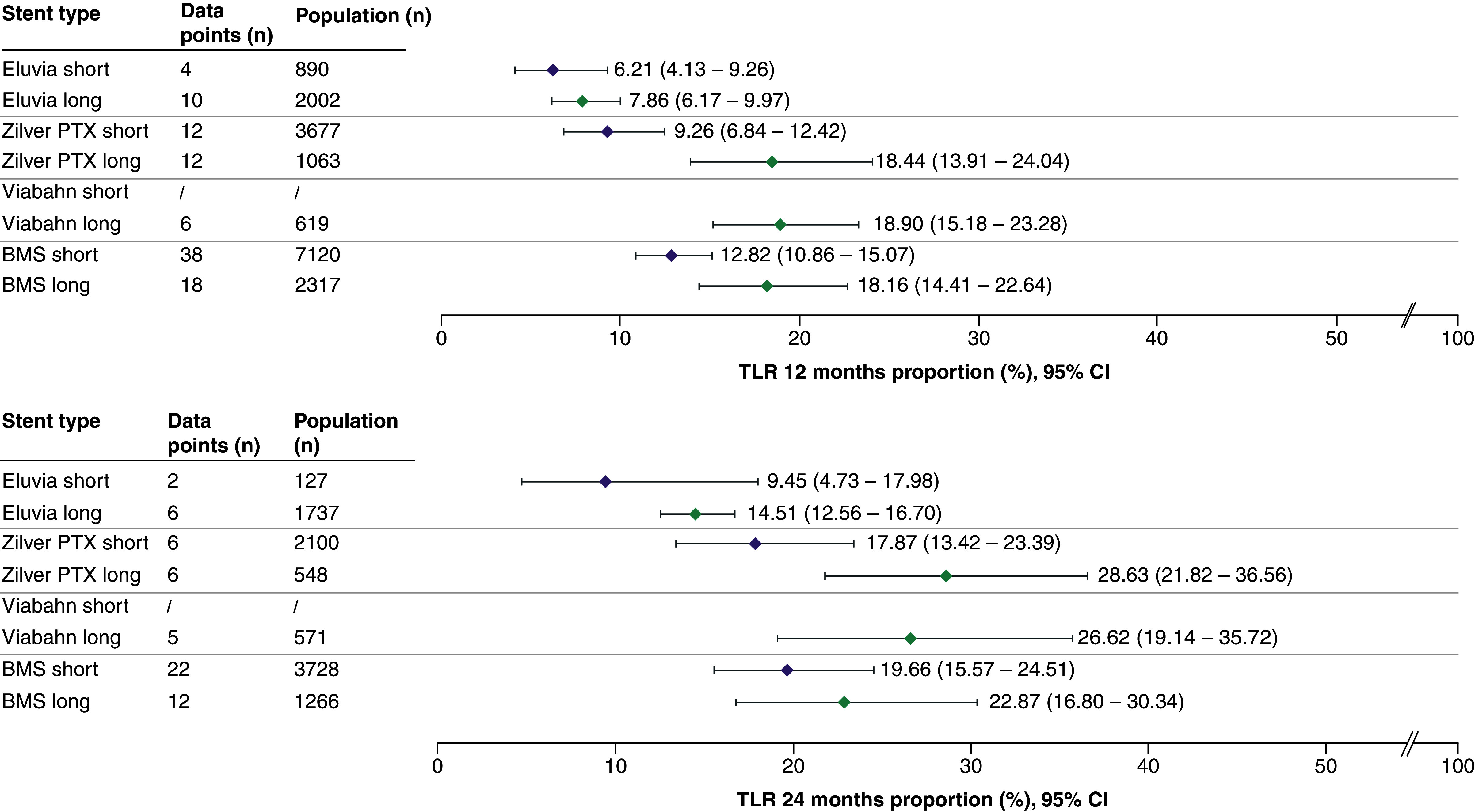
Summary forest plots showing target lesion revascularization for short and long lesions at 12 and 24 months post stenting. The pooled estimates of TLR (and the corresponding 95% CI values) are shown for each stent type in short (orange) or long (blue) lesions at 12 months (top panel) or 24 months (bottom panel). The full forest plot for each stent type at each time point can be found in Supplementary Figures 38–43. BMS: Bare metal stent; CI: Confidence interval; TLR: Target lesion revascularization.

The TLR rate was similar for long and short lesions treated with Eluvia (Figure 5). Meanwhile, the TLR rate for long lesions treated with Zilver PTX was higher than that for short lesions. As no studies reported the use of Viabahn in short lesions, no subgroup analysis was possible. Considerable overlap in 95% CIs was observed when comparing the performance of BMS in long and short lesions.

The results of the sensitivity analyses, based on the reporting of core laboratory adjudication (primary patency: Supplementary Table 5 & Supplementary Figures 15–21, TLR: Supplementary Table 7 & Supplementary Figures 44–49) and Downs and Black quality assessment (primary patency: Supplementary Table 6 & Supplementary Figures 22–29, TLR: Supplementary Table 8 & Supplementary Figures 50–57), did not substantially alter the results of the main analysis. However, including only the studies that reported using core laboratory adjudication in the sensitivity analysis increased the variability of the estimated effect (i.e., widened the 95% CIs).

### Synthesis of results for the secondary outcomes

**Figure 6.**
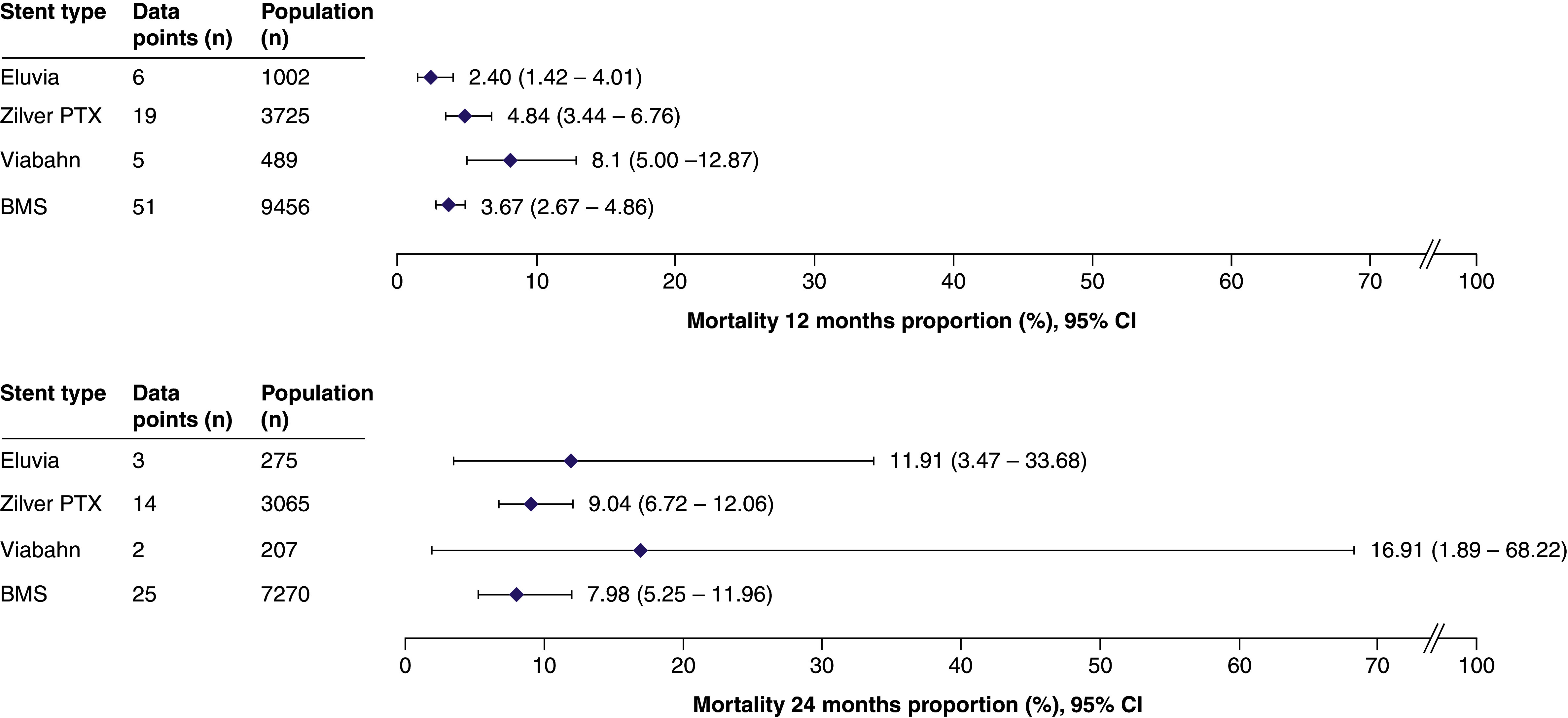
Summary forest plots showing mortality at 12 and 24 months post stenting. The pooled estimates of mortality (and the corresponding 95% CI values) are shown for each stent type at 12 months (top panel) or 24 months (bottom panel). The full forest plot for each stent type at each time point can be found in Supplementary Figures 58–65. These data were extracted from the following references: Eluvia [11,12,28,49,78,126,131,148]; Zilver PTX [11,13,36,43,55,60,65,70,71,74,77,87,91,100,101,110,116,141,144,150,152,154,155,158]; Viabahn [31,58,122,141,154]; BMS [13,23,24,29,30,33,38,39,41,43,46,51,54,56–58,68,70,74,79,81,83,89,94,98,103,106,118,119,123–126,128,138,140,144,146,147,154,156,157]. BMS: Bare metal stent: CI: Confidence interval.

Mortality was reported in 78/141 studies (55%) for 14,672 patients (81 data points) and 10,817 patients (44 data points) at 12 and 24 months, respectively. The performance of the stents appeared similar with regards to this outcome (Figure 6).

Fewer mortality data points were available for the subgroup analysis than in the main analysis (Supplementary Figures 66–70). At 12 months, mortality rates were comparable across lesion lengths for all stents. At 24 months, more variation in mortality rate was observed, with long lesions resulting in higher mortality rates than short lesions. The results of the sensitivity analysis, based on the reporting of core laboratory adjudication (Supplementary Table 9 & Supplementary Figures 71–76) and Downs and Black quality assessment, (Supplementary Table 10 & Supplementary Figures 77–84) were comparable to those of the main analysis.

The other secondary outcomes were inconsistently reported and did not generate meaningful results for all stent types. The incidence of major amputations was reported by 52/141 (37%) of studies and was higher at 24 months (range: 1.2–2.3%) than at 12 months (range: 0.5–1.4%) (Supplementary Table 11 & Supplementary Figures 85–92). The number of studies reporting stent fractures was also relatively low (45/141, 32%). This outcome was similar at 12 months (range: 0.2–4.8%) and 24 months (range: 0.0–4.4%) but was highly heterogeneous, likely due to the low number of data points (Supplementary Table 12 & Supplementary Figures 93–98). We also attempted to examine clinical improvement associated with each stent type (range: 79.7–91.0% at 12 months) at both timepoints (Supplementary Table 13 & Supplementary Figures 99–101); however, low study numbers reporting data in our timeframe (26/141, 18%) and the heterogeneity between studies in reporting this outcome limited the utility of the findings. The analysis of walking impairment questionnaire scores (reported by 10/141 [7%] studies) was also not informative due to insufficient data (data not shown).

## Discussion

This meta-analysis of proportions included 141 studies evaluating the performance of four stent types in patients with femoropopliteal disease, with a focus on primary patency and TLR at 12- and 24-months post stenting. We found that Eluvia demonstrated consistent performance (i.e., similar primary patency and TLR rates) across lesion lengths and follow-up times, which was not the case for the other stents. As expected, stent performance decreased over time, with lower performance at 24 months than 12 months. Although the reasons for variability in stent performance were not investigated in this study, they may be linked to differences in stent, lesion and/or patient characteristics.

Long or complex lesions in the femoropopliteal segment are especially challenging to treat as they are subjected to continuous mechanical stress [159]. Such lesions are associated with an increased risk of intimal hyperplasia (with subsequent restenosis or reocclusion), stent fracture and ultimately, lower patency and freedom from TLR rates [6]. To date, few studies have demonstrated the successful application of nitinol stents in long, complex femoropopliteal lesions [42,49,160]. Paclitaxel-eluting stents may be especially important in the treatment of longer lesions at a high risk of restenosis [49]. Eluvia is designed to achieve controlled, sustained drug release over time [161], whereas Zilver PTX releases the drug at a faster rate [162]. This may be one explanation for the difference in the performance of these stents in long lesions observed in the present study.

Patient characteristics are an important factor during stent selection. Patients with long and/or complex femoropopliteal lesions often present with more severe disease, characterised by higher rates of CTO, and the presence of CLTI [81,160,163]. In accordance, we found that more patients with long than with short lesions presented with CTO (68.3 vs 44.2%) or CLTI (37.8 vs 34.5%). The overall characteristics of patients included in our study align with the femoropopliteal patient profile reported by patient registries [78,146]. However, the effect of patient characteristics on outcomes fell outside the scope of the present study and was not controlled for in our analysis. Ultimately, patient-level data will be required to address the true impact of patient characteristics on stent selection and performance. At present, our understanding of stent performance in diverse patient populations is also limited by the fact that many of the registry datasets presented at symposia are rarely formally disseminated. Greater access to this information would enable clinicians to make more informed treatment decisions and support evidence-based market access for new medical technologies.

Inconsistent outcome reporting emerged as a notable barrier in this analysis, particularly as it prevented a detailed evaluation of secondary outcomes. We found that primary patency and TLR often lacked clear definitions and required conversion for cross-study comparisons. For instance, TLR was reported as CD-TLR or any TLR and primary patency had to sometimes be derived from restenosis. The consistent reporting of outcomes such as CD-TLR is particularly important for patients with advanced femoropopliteal disease, as it provides a clinically meaningful measure of treatment failure and a reliable indicator of whether reintervention is required. Secondary outcomes, such as stent fractures and clinical improvement, were only reported sporadically. Although many studies presented walking distance scores, the heterogeneity in reporting prevented any meaningful analysis of this outcome. Overall, the current lack of consistent outcome reporting complicates the assessment of a stent’s true clinical benefit. We recommend the adoption of standardized definitions and uniform reporting criteria to enable more accurate medical device comparisons.

Considering that only around a third of the included studies used core laboratory adjudication, the need for the standardization of reporting practices is clear. Adjudication by an independent core laboratory reduces operator bias and ensures that key outcomes are accurately reported. As such, it is a key feature of RCT design. Discrepancies between operator- and core-laboratory-assessed outcomes have been documented [25,164]. Thus, the use of core laboratory adjudication in femoropopliteal stenting may help mitigate operator bias such as the underestimation of residual stenosis. However, because the use of core laboratory adjudication is still not routinely implemented, focusing on studies that included this quality assurance process paradoxically reduced the robustness of the reported data in the sensitivity analysis. Thus, future studies should prioritise core laboratory adjudication and standardized outcome definitions to enhance cross-study comparability. Establishing a Clinical Events Committee to align best practices for long-term outcome reporting represents a valuable next step, while supplementing RCT and real-word data with findings from Delphi consensus studies promises to further enhance collaboration among experts to improve patient outcomes [165].

Similarly, sensitivity analyses based on study quality (assessed using the Downs and Black Quality Appraisal tool) did not alter our overall conclusions. It is possible that studies rated as ‘poor’ may not have reported values that differed significantly from those originating from studies with a better rating. This may, in part, reflect a limitation of the Downs and Black Quality Appraisal tool, whereby single-arm studies are inherently penalised because of their design rather than the methodological rigour with which they were conducted. Given that this analysis was not restricted to comparative data, the inclusion of single-arm studies remains a valuable and appropriate source of information to characterise outcomes associated with specific stent types.

A key strength of this study is that it used a combination of randomized, prospective, nonrandomized and retrospective studies to provide a robust, yet generalisable, measure of stent performance in femoropopliteal lesions. Although the nature of a pooled rates meta-analysis prevents direct comparison, the lack of overlap between the 95% CIs of Eluvia and those of the other stents indicates that this stent may exhibit superior performance across lesion lengths and follow-up timepoints.

This study also had limitations. Key end points such as primary patency and TLR were not consistently defined across the included studies. The derivation of primary patency or TLR via conversion may have introduced some misclassification bias; however, these conversions were only performed when certain stringent conditions were met. Studies that did not report the mean/median lesion length (e.g., they reported the median lesion length or the mean lesion length for the whole population but not a subset treated with the stent of interest) could not be included in the subgroup analyses. Only 12- and 24-month follow-up durations were investigated. Moreover, outcomes were more frequently reported at 12 than at 24 months (152 vs 94 data points for primary patency; 106 vs 65 data points for TLR), reducing the robustness of the long-term data. We cannot guarantee the quality of the included data as only 27.5% of the studies reported using core laboratory adjudication and almost a third did not achieve a Downs and Black rating of ‘fair’ or above. The inclusion of single-arm studies may have reduced the reliability of the pooled estimates by introducing confounding in the patient and study characteristics that could not have been fully adjusted for in the absence of direct comparators. The BMS group comprises stents made by different manufacturers and with designs, which inevitably introduces intragroup heterogeneity. Nonetheless, previous studies have adopted the same grouping of BMSs, and specifically self-expanding bare nitinol stents [12,22,53,59,62–64,68–70,72,76,81,85,86,96,97,100,109,111,129,132,143,145,154,157]. The generalised linear mixed model used enabled the estimation of heterogeneity while considering variability across individual studies. Although this approach is robust, it is characterised by wide 95% CIs, particularly when one of the studies reports zero events for an outcome. An expected limitation of pooled analyses is that they rely on aggregate (i.e., cohort means) rather than primary patient-level data. Finally, we acknowledge that the results of this meta-analysis of proportions are not a substitute for direct, comparative data. As such, future studies should build on this work by comparing the performance of different stent types in RCTs and individual patients. In addition, an analysis of longer-term follow-up is needed to effectively capture data on stent durability and late-stage complications.

## Conclusion

The lack of RCTs comparing stent performance in PAD limits the feasibility of conducting a head-to-head meta-analysis directly comparing stent effectiveness within the femoropopliteal segment. The present meta-analysis of proportions provides pooled estimates and corresponding CIs for outcomes related to the performance of four common stent types in both short and long femoropopliteal lesions across two key timepoints. The underlying distribution of effects did on occasion differ between the stent types. We found that Eluvia performed consistently well in both short and long lesions, even at 24-month follow-up. Although they are no substitute for direct, comparative data, our findings offer valuable insights into stent performance, helping clinicians make more informed, case-by-case treatment decisions based on lesion characteristics and long-term outcomes. Additionally, this study demonstrates how the inclusion of long-lesion data in clinical practice can help guide stent selection strategies. Conclusive comparative findings from RCTs and studies involving patient-level data are needed to build on this work.

## Summary points

- This systematic literature review included 141 studies on four stent types in femoropopliteal arterial disease and pooled their results in a meta-analysis of proportions.
- The four stent types are bare metal stents (BMS), a polymer-based paclitaxel-eluting stent (Eluvia), a covered stent (Viabahn) and a polymer-free paclitaxel-coated stent (Zilver PTX).
- Primary patency and target lesion revascularization were pooled at 12 and 24 months, and a subgroup analysis was done for short lesions (<150 mm) and long lesions (≥150 mm).
- We found that only one stent, Eluvia, demonstrated consistent performance (i.e., similar primary patency and target lesion revascularization rates) across lesion lengths and follow-up times.
- As expected, stent performance in general decreased over time, with lower performance at 24 months than 12 months.
- Mortality rates for short lesions were stable for all stent types, but more variable for long lesions.
- Inconsistent definitions were a potential barrier to the pooling of data across clinical studies.
- Standardization in the reporting of outcomes and the use of core laboratory adjudication would allow for better, cross-study comparison.

## Supplementary Material


