# Supplementary material for: Performance of stenting in femoropopliteal disease: a systematic literature review and meta-analysis of proportions
Source: J Comp Eff Res. 2026 Mar 10;15(4):e250152. doi: 10.57264/cer-2025-0152 (PMC13044811; doi:10.57264/cer-2025-0152)
Supplement: Supplementary file 1 [file cer-15-250152-s1.docx]

**Supplementary Data**

**Performance of stenting in femoropopliteal disease: Systematic literature review and meta-analysis of proportion**

**List of supplementary tables**

[Supplementary Table 1. The PRISMA checklist](#_Toc216797808)

[Supplementary Table 2. Summary of reported lesion lengths](#_Toc216797809)

[Supplementary Table 3. Further patient and lesion characteristics and core laboratory adjudication](#_Toc216797810)

[Supplementary Table 4. Downs and Black Quality Appraisal and characteristics of the included studies](#_Toc216797811)

[Supplementary Table 5. Primary patency at 12 and 24 months post stenting based on the use of core laboratory adjudication*](#_Toc216797812)

[Supplementary Table 6. Primary patency at 12 and 24 months post stenting based on the Downs and Black Quality Appraisal score*](#_Toc216797813)

[Supplementary Table 7. Target lesions revascularisation at 12 and 24 months post stenting based on the use of core laboratory adjudication*](#_Toc216797814)

[Supplementary Table 8. Target lesion revascularisation at 12 and 24 months post stenting based on the Downs and Black Quality Appraisal score*](#_Toc216797815)

[Supplementary Table 9. Mortality at 12 and 24 months post stenting based on the use of core laboratory adjudication*](#_Toc216797816)

[Supplementary Table 10. Mortality at 12 and 24 months post stenting based on the Downs and Black Quality Appraisal score*](#_Toc216797817)

[Supplementary Table 11. Major amputations at 12 and 24 months post stenting](#_Toc216797818)

[Supplementary Table 12. Stent fractures at 12 and 24 months post stenting](#_Toc216797819)

[Supplementary Table 13. Clinical improvement at 12 months post stenting*](#_Toc216797820)

**List of supplementary figures**

[Supplementary Figure 1. Primary patency at 12 months for Eluvia™.](#_Toc216797821)

[Supplementary Figure 2. Primary patency at 12 months for Zilver® PTX®.](#_Toc216797822)

[Supplementary Figure 3. Primary patency at 12 months for Viabahn.](#_Toc216797823)

[Supplementary Figure 4. Primary patency at 12 months for BMS.](#_Toc216797824)

[Supplementary Figure 5. Primary patency at 24 months for Eluvia™.](#_Toc216797825)

[Supplementary Figure 6. Primary patency at 24 months for Zilver® PTX®.](#_Toc216797826)

[Supplementary Figure 7. Primary patency at 24 months for Viabahn.](#_Toc216797827)

[Supplementary Figure 8. Primary patency at 24 months for BMS.](#_Toc216797828)

[Supplementary Figure 9. Primary patency in short and long lesions at 12 months for Eluvia™.](#_Toc216797829)

[Supplementary Figure 10. Primary patency in short and long lesions at 12 months for Zilver® PTX®.](#_Toc216797830)

[Supplementary Figure 11. Primary patency in short and long lesions at 12 months for BMS.](#_Toc216797831)

[Supplementary Figure 12. Primary patency in short and long lesions at 24 months for Eluvia™.](#_Toc216797832)

[Supplementary Figure 13. Primary patency in short and long lesions at 24 months for Zilver® PTX®.](#_Toc216797833)

[Supplementary Figure 14. Primary patency in short and long lesions at 24 months for BMS.](#_Toc216797834)

[Supplementary Figure 15. Primary patency at 12 months for Eluvia™ according to studies that reported using core laboratory adjudication.](#_Toc216797835)

[Supplementary Figure 16. Primary patency at 12 months for Zilver® PTX® according to studies that reported using core laboratory adjudication.](#_Toc216797836)

[Supplementary Figure 17. Primary patency at 12 months for Viabahn according to studies that reported using core laboratory adjudication.](#_Toc216797837)

[Supplementary Figure 18. Primary patency at 12 months for BMS according to studies that reported using core laboratory adjudication.](#_Toc216797838)

[Supplementary Figure 19. Primary patency at 24 months for Zilver® PTX® according to studies that reported using core laboratory adjudication.](#_Toc216797839)

[Supplementary Figure 20. Primary patency at 24 months for Viabahn according to studies that reported using core laboratory adjudication.](#_Toc216797840)

[Supplementary Figure 21. Primary patency at 24 months for BMS according to studies that reported using core laboratory adjudication.](#_Toc216797841)

[Supplementary Figure 22. Primary patency at 12 months for Eluvia™ according to studies with a Downs and Black Quality Appraisal rating of fair or above.](#_Toc216797842)

[Supplementary Figure 23. Primary patency at 12 months for Zilver® PTX® according to studies with a Downs and Black Quality Appraisal rating of fair or above.](#_Toc216797843)

[Supplementary Figure 24. Primary patency at 12 months for Viabahn according to studies with a Downs and Black Quality Appraisal rating of fair or above.](#_Toc216797844)

[Supplementary Figure 25. Primary patency at 12 months for BMS according to studies with a Downs and Black Quality Appraisal rating of fair or above.](#_Toc216797845)

[Supplementary Figure 26. Primary patency at 24 months for Eluvia™ according to studies with a Downs and Black Quality Appraisal rating of fair or above.](#_Toc216797846)

[Supplementary Figure 27. Primary patency at 24 months for Zilver® PTX® according to studies with a Downs and Black Quality Appraisal rating of fair or above.](#_Toc216797847)

[Supplementary Figure 28. Primary patency at 24 months for Viabahn according to studies with a Downs and Black Quality Appraisal rating of fair or above.](#_Toc216797848)

[Supplementary Figure 29. Primary patency at 24 months for BMS according to studies with a Downs and Black Quality Appraisal rating of fair or above.](#_Toc216797849)

[Supplementary Figure 30. Target lesion revascularisation at 12 months for Eluvia™.](#_Toc216797850)

[Supplementary Figure 31. Target lesion revascularisation at 12 months for Zilver® PTX®.](#_Toc216797851)

[Supplementary Figure 32. Target lesion revascularisation at 12 months for Viabahn.](#_Toc216797852)

[Supplementary Figure 33. Target lesion revascularisation at 12 months for BMS.](#_Toc216797853)

[Supplementary Figure 34. Target lesion revascularisation at 24 months for Eluvia™.](#_Toc216797854)

[Supplementary Figure 35. Target lesion revascularisation at 24 months for Zilver® PTX®.](#_Toc216797855)

[Supplementary Figure 36. Target lesion revascularisation at 24 months for Viabahn.](#_Toc216797856)

[Supplementary Figure 37. Target lesion revascularisation at 24 months for BMS.](#_Toc216797857)

[Supplementary Figure 38. Target lesion revascularisation in short and long lesions at 12 months for Eluvia™.](#_Toc216797858)

[Supplementary Figure 39. Target lesion revascularisation in short and long lesions at 12 months for Zilver® PTX®.](#_Toc216797859)

[Supplementary Figure 40. Target lesion revascularisation in short and long lesions at 12 months for BMS.](#_Toc216797860)

[Supplementary Figure 41. Target lesion revascularisation in short and long lesions at 24 months for Eluvia™.](#_Toc216797861)

[Supplementary Figure 42. Target lesion revascularisation in short and long lesions at 24 months for Zilver® PTX®.](#_Toc216797862)

[Supplementary Figure 43. Target lesion revascularisation in short and long lesions at 24 months for BMS.](#_Toc216797863)

[Supplementary Figure 44. Target lesion revascularisation at 12 months for Eluvia™ according to studies that reported using core laboratory adjudication.](#_Toc216797864)

[Supplementary Figure 45. Target lesion revascularisation at 12 months for Zilver® PTX® according to studies that reported using core laboratory adjudication.](#_Toc216797865)

[Supplementary Figure 46. Target lesion revascularisation at 12 months for Viabahn according to studies that reported using core laboratory adjudication.](#_Toc216797866)

[Supplementary Figure 47. Target lesion revascularisation at 12 months for BMS according to studies that reported using core laboratory adjudication.](#_Toc216797867)

[Supplementary Figure 48. Target lesion revascularisation at 24 months for Zilver® PTX® according to studies that reported using core laboratory adjudication.](#_Toc216797868)

[Supplementary Figure 49. Target lesion revascularisation at 24 months for BMS according to studies that reported using core laboratory adjudication.](#_Toc216797869)

[Supplementary Figure 50. Target lesion revascularisation at 12 months for Eluvia™ according to studies with a Downs and Black Quality Appraisal rating of fair or above.](#_Toc216797870)

[Supplementary Figure 51. Target lesion revascularisation at 12 months for Zilver® PTX® according to studies with a Downs and Black Quality Appraisal rating of fair or above.](#_Toc216797871)

[Supplementary Figure 52. Target lesion revascularisation at 12 months for Viabahn according to studies with a Downs and Black Quality Appraisal rating of fair or above.](#_Toc216797872)

[Supplementary Figure 53. Target lesion revascularisation at 12 months for BMS according to studies with a Downs and Black Quality Appraisal rating of fair or above.](#_Toc216797873)

[Supplementary Figure 54. Target lesion revascularisation at 24 months for Eluvia™ according to studies with a Downs and Black Quality Appraisal rating of fair or above.](#_Toc216797874)

[Supplementary Figure 55. Target lesion revascularisation at 24 months for Zilver® PTX® according to studies with a Downs and Black Quality Appraisal rating of fair or above.](#_Toc216797875)

[Supplementary Figure 56. Target lesion revascularisation at 24 months for Viabahn according to studies with a Downs and Black Quality Appraisal rating of fair or above.](#_Toc216797876)

[Supplementary Figure 57. Target lesion revascularisation at 24 months for BMS according to studies with a Downs and Black Quality Appraisal rating of fair or above.](#_Toc216797877)

[Supplementary Figure 58. Mortality at 12 months for Eluvia™.](#_Toc216797878)

[Supplementary Figure 59. Mortality at 12 months for Zilver® PTX®.](#_Toc216797879)

[Supplementary Figure 60. Mortality at 12 months for Viabahn.](#_Toc216797880)

[Supplementary Figure 61. Mortality at 12 months for BMS.](#_Toc216797881)

[Supplementary Figure 62. Mortality at 24 months for Eluvia™.](#_Toc216797882)

[Supplementary Figure 63. Mortality at 24 months for Zilver® PTX®.](#_Toc216797883)

[Supplementary Figure 64. Mortality at 24 months for Viabahn.](#_Toc216797884)

[Supplementary Figure 65. Mortality at 24 months for BMS.](#_Toc216797885)

[Supplementary Figure 66. Mortality for short and long lesions at 12 months for Eluvia™.](#_Toc216797886)

[Supplementary Figure 67. Mortality for short and long lesions at 12 months for Zilver® PTX®.](#_Toc216797887)

[Supplementary Figure 68. Mortality for short and long lesions at 12 months for BMS.](#_Toc216797888)

[Supplementary Figure 69. Mortality for short and long lesions at 24 months for Zilver® PTX®.](#_Toc216797889)

[Supplementary Figure 70. Mortality for short and long lesions at 24 months for BMS](#_Toc216797890)

[Supplementary Figure 71. Mortality at 12 months for Eluvia™ according to studies that reported using core laboratory adjudication.](#_Toc216797891)

[Supplementary Figure 72. Mortality at 12 months for Zilver® PTX® according to studies that reported using core laboratory adjudication.](#_Toc216797892)

[Supplementary Figure 73. Mortality at 12 months for Viabahn according to studies that reported using core laboratory adjudication.](#_Toc216797893)

[Supplementary Figure 74. Mortality at 12 months for BMS according to studies that reported using core laboratory adjudication.](#_Toc216797894)

[Supplementary Figure 75. Mortality at 24 months for Zilver® PTX® according to studies that reported using core laboratory adjudication.](#_Toc216797895)

[Supplementary Figure 76. Mortality at 24 months for BMS according to studies that reported using core laboratory adjudication.](#_Toc216797896)

[Supplementary Figure 77. Mortality at 12 months for Eluvia™ according to studies with a Downs and Black Quality Appraisal rating of fair or above.](#_Toc216797897)

[Supplementary Figure 78. Mortality at 12 months for Zilver® PTX® according to studies with a Downs and Black Quality Appraisal rating of fair or above.](#_Toc216797898)

[Supplementary Figure 79. Mortality at 12 months for Viabahn according to studies with a Downs and Black Quality Appraisal rating of fair or above.](#_Toc216797899)

[Supplementary Figure 80. Mortality at 12 months for BMS according to studies with a Downs and Black Quality Appraisal rating of fair or above.](#_Toc216797900)

[Supplementary Figure 81. Mortality at 24 months for Eluvia™ according to studies with a Downs and Black Quality Appraisal rating of fair or above.](#_Toc216797901)

[Supplementary Figure 82. Mortality at 24 months for Zilver® PTX® according to studies with a Downs and Black Quality Appraisal rating of fair or above.](#_Toc216797902)

[Supplementary Figure 83. Mortality at 24 months for Viabahn according to studies with a Downs and Black Quality Appraisal rating of fair or above.](#_Toc216797903)

[Supplementary Figure 84. Mortality at 24 months for BMS according to studies with a Downs and Black Quality Appraisal rating of fair or above.](#_Toc216797904)

[Supplementary Figure 85. Major amputations at 12 months for Eluvia™.](#_Toc216797905)

[Supplementary Figure 86. Major amputations at 12 months for Zilver® PTX®.](#_Toc216797906)

[Supplementary Figure 87. Major amputations at 12 months for Viabahn®.](#_Toc216797907)

[Supplementary Figure 88. Major amputations at 12 months for BMS.](#_Toc216797908)

[Supplementary Figure 89. Major amputations at 24 months for Eluvia™.](#_Toc216797909)

[Supplementary Figure 90. Major amputations at 24 months for Zilver® PTX®.](#_Toc216797910)

[Supplementary Figure 91. Major amputations at 24 months for Viabahn.](#_Toc216797911)

[Supplementary Figure 92. Major amputations at 24 months for BMS.](#_Toc216797912)

[Supplementary Figure 93. Stent fractures at 12 months for Eluvia™.](#_Toc216797913)

[Supplementary Figure 94. Stent fractures at 12 months for Zilver® PTX®.](#_Toc216797914)

[Supplementary Figure 95. Stent fractures at 12 months for Viabahn.](#_Toc216797915)

[Supplementary Figure 96. Stent fractures at 12 months for BMS.](#_Toc216797916)

[Supplementary Figure 97. Stent fractures at 24 months for Zilver® PTX®.](#_Toc216797917)

[Supplementary Figure 98. Stent fractures at 24 months for BMS.](#_Toc216797918)

[Supplementary Figure 99. Clinical improvement at 12 months for Eluvia™.](#_Toc216797919)

[Supplementary Figure 100. Clinical improvement at 12 months for Zilver® PTX®.](#_Toc216797920)

[Supplementary Figure 101. Clinical improvement at 12 months for BMS.](#_Toc216797921)

#

# Supplementary tables

Supplementary Table 1. The PRISMA checklist

| **Section and Topic** | **Item #** | **Checklist item** | **Location where item is reported** |
| --- | --- | --- | --- |
| **TITLE** | | |  |
| Title | 1 | Identify the report as a systematic review. | Line 1-2 |
| **ABSTRACT** | | |  |
| Abstract | 2 | See the PRISMA 2020 for Abstracts checklist. |  |
| **INTRODUCTION** | | |  |
| Rationale | 3 | Describe the rationale for the review in the context of existing knowledge. | Line 33 – 40 |
| Objectives | 4 | Provide an explicit statement of the objective(s) or question(s) the review addresses. | Line 41 - 45 |
| **METHODS** | | |  |
| Eligibility criteria | 5 | Specify the inclusion and exclusion criteria for the review and how studies were grouped for the syntheses. | Line 56 - 59 |
| Information sources | 6 | Specify all databases, registers, websites, organisations, reference lists and other sources searched or consulted to identify studies. Specify the date when each source was last searched or consulted. | Line 49 - 52 |
| Search strategy | 7 | Present the full search strategies for all databases, registers and websites, including any filters and limits used. | Line 51 – 52 |
| Selection process | 8 | Specify the methods used to decide whether a study met the inclusion criteria of the review, including how many reviewers screened each record and each report retrieved, whether they worked independently, and if applicable, details of automation tools used in the process. | Line 52 – 62 |
| Data collection process | 9 | Specify the methods used to collect data from reports, including how many reviewers collected data from each report, whether they worked independently, any processes for obtaining or confirming data from study investigators, and if applicable, details of automation tools used in the process. | Line 60 - 62 |
| Data items | 10a | List and define all outcomes for which data were sought. Specify whether all results that were compatible with each outcome domain in each study were sought (e.g. for all measures, time points, analyses), and if not, the methods used to decide which results to collect. | Line 62 – 67 |
|  | 10b | List and define all other variables for which data were sought (e.g. participant and intervention characteristics, funding sources). Describe any assumptions made about any missing or unclear information. | Line 62 - 77 |
| Study risk of bias assessment | 11 | Specify the methods used to assess risk of bias in the included studies, including details of the tool(s) used, how many reviewers assessed each study and whether they worked independently, and if applicable, details of automation tools used in the process. | Line 88 - 90 |
| Effect measures | 12 | Specify for each outcome the effect measure(s) (e.g. risk ratio, mean difference) used in the synthesis or presentation of results. | Line 80 - 83 |
| Synthesis methods | 13a | Describe the processes used to decide which studies were eligible for each synthesis (e.g. tabulating the study intervention characteristics and comparing against the planned groups for each synthesis (item #5)). | Line 68 - 77 |
|  | 13b | Describe any methods required to prepare the data for presentation or synthesis, such as handling of missing summary statistics, or data conversions. | Line 68 - 77 |
|  | 13c | Describe any methods used to tabulate or visually display results of individual studies and syntheses. | Line 84 - 86 |
|  | 13d | Describe any methods used to synthesize results and provide a rationale for the choice(s). If meta-analysis was performed, describe the model(s), method(s) to identify the presence and extent of statistical heterogeneity, and software package(s) used. | Line 79 -83 |
|  | 13e | Describe any methods used to explore possible causes of heterogeneity among study results (e.g. subgroup analysis, meta-regression). | Line 88 - 94 |
|  | 13f | Describe any sensitivity analyses conducted to assess robustness of the synthesized results. | Line 88 - 94 |
| Reporting bias assessment | 14 | Describe any methods used to assess risk of bias due to missing results in a synthesis (arising from reporting biases). |  |
| Certainty assessment | 15 | Describe any methods used to assess certainty (or confidence) in the body of evidence for an outcome. | Line 82 – 83 |
| **RESULTS** | | |  |
| Study selection | 16a | Describe the results of the search and selection process, from the number of records identified in the search to the number of studies included in the review, ideally using a flow diagram. | Line 97 - 100 |
|  | 16b | Cite studies that might appear to meet the inclusion criteria, but which were excluded, and explain why they were excluded. |  |
| Study characteristics | 17 | Cite each included study and present its characteristics. | Table 1 |
| Risk of bias in studies | 18 | Present assessments of risk of bias for each included study. | Supplementary table 4 |
| Results of individual studies | 19 | For all outcomes, present, for each study: (a) summary statistics for each group (where appropriate) and (b) an effect estimate and its precision (e.g. confidence/credible interval), ideally using structured tables or plots. | Supplementary table 3, supplementary figures 1 - 101 |
| Results of syntheses | 20a | For each synthesis, briefly summarise the characteristics and risk of bias among contributing studies. | Line 101 - 112 |
|  | 20b | Present results of all statistical syntheses conducted. If meta-analysis was done, present for each the summary estimate and its precision (e.g. confidence/credible interval) and measures of statistical heterogeneity. If comparing groups, describe the direction of the effect. | Line 114 – 157, supplementary tables and figures |
|  | 20c | Present results of all investigations of possible causes of heterogeneity among study results. | Line 114 – 157 |
|  | 20d | Present results of all sensitivity analyses conducted to assess the robustness of the synthesized results. | Line 131 - 136 |
| Reporting biases | 21 | Present assessments of risk of bias due to missing results (arising from reporting biases) for each synthesis assessed. |  |
| Certainty of evidence | 22 | Present assessments of certainty (or confidence) in the body of evidence for each outcome assessed. | Manuscript figures and Supplementary figures |
| **DISCUSSION** | | |  |
| Discussion | 23a | Provide a general interpretation of the results in the context of other evidence. | Line 159 - 186 |
|  | 23b | Discuss any limitations of the evidence included in the review. | Line 214 - 227 |
|  | 23c | Discuss any limitations of the review processes used. | Line 214 - 227 |
|  | 23d | Discuss implications of the results for practice, policy, and future research. | Line 195 – 196, 204 – 208 |
| **OTHER INFORMATION** | | |  |
| Registration and protocol | 24a | Provide registration information for the review, including register name and registration number, or state that the review was not registered. | Line 47 |
|  | 24b | Indicate where the review protocol can be accessed, or state that a protocol was not prepared. | Line 47 |
|  | 24c | Describe and explain any amendments to information provided at registration or in the protocol. | Line 92 - 94 |
| Support | 25 | Describe sources of financial or non-financial support for the review, and the role of the funders or sponsors in the review. | Title page |
| Competing interests | 26 | Declare any competing interests of review authors. | Title page |
| Availability of data, code and other materials | 27 | Report which of the following are publicly available and where they can be found: template data collection forms; data extracted from included studies; data used for all analyses; analytic code; any other materials used in the review. | Supplementary material |

Supplementary Table 2. Summary of reported lesion lengths

| **Eluvia** | **Data points*** | **Mean lesion length (mm)** | **Range (mm)** |
| --- | --- | --- | --- |
| Overall | 17 | 159.4 | 70.8-230.0 |
| Short lesions | 5 | 89.1 | 70.8-140.0 |
| Long lesions | 12 | 188.6 | 160.0-230.0 |
| Lesion length not reported | 0 | | |
| **Zilver PTX** | **Data points*** | **Mean lesion length** | **Range (min)** |
| Overall | 39 | 159.6 | 64.2-330.0 |
| Short lesions | 19 | 108.5 | 64.2-147.0 |
| Long lesions | 20 | 208.1 | 155.5-330.0 |
| Lesion length not reported | 1 | | |
| **Viabahn** | **Data points*** | **Mean lesion length** | **Range (min)** |
| Overall | 11 | 226.4 | 173.0-290.0 |
| Short lesions | 0 | 0 | 0 |
| Long lesions | 11 | 226.4 | 173.0-290.0 |
| Lesion length not reported | 2 | | |
| **BMS** | **Data points*** | **Mean lesion length** | **Range (min)** |
| Overall | 96 | 140.5 | 37.0-330.0 |
| Short lesions | 59 | 96.7 | 37.0-148.0 |
| Long lesions | 37 | 209.9 | 150.0-330.0 |
| Lesion length not reported | 7 | | |

* Number of study arms. When subsequent studies were done on the same population, the information about lesion length was not included again to prevent duplication. Abbreviations: NR, not reported; PP, primary patency.

Supplementary Table 3. Further patient and lesion characteristics and core laboratory adjudication

| Paper ID | Calcification | TASC classification | Claudicants | Diabetes (%) | Hypertension (%) | Cardiovascular disease (%) | Renal function | Core-lab adjudication of doppler sound scans and angiograms |  |
| --- | --- | --- | --- | --- | --- | --- | --- | --- | --- |
| Abi-Khalil et al., 2022 | None 2.9% Mild 8.6% Moderate 37.1% Severe 51.4% | A 68.6% B 22.9% C 8.6% D 0% | NR | 25.7 | 60 | NR | Creatinine >1.4 mg/dl 11.4% | NR |  |
| Abi-Khalil et al., 2022 | None 22.2% Mild 14.8% Moderate 29.6% Severe 33.3% | A 55.6% B 29.6% C 7.4% D 7.4% | NR | 22.2 | 81.5 | NR | Creatinine >1.4 mg/dl 1% | NR |  |
| AbuRahma et al., 2022 | NR | A - C 55% D 45% | 32 | 43 | 57 | 51 (coronary artery disease) | Chronic kidney disease 18% | No |  |
| Armstrong et al., 2014 | None-mild: 80 Moderate-severe:20 | NR | NR | 59 | 89 | Coronary artery disease - 57 Heart failure - 30 | GFR - 68mg/dL | No |  |
| Armstrong et al., 2014 | None-mild: 61 Moderate-severe: 39 | NR | NR | 60 | 87 | Coronary arterial disease - 39 Heart failure - 31 | GFR - 60mg/dL | No |  |
| Armstrong et al., 2020 | Heavily calcified lesion 41.5 | NR | NR | 44.9 | 89.8 | NR | Chronic kidney disease 14.4 | Yes |  |
| Astarcıoglu et al., 2017 | NR | D 100% | NR | 47.9 | 77 | Coronary artery disease 58.3 | Renal failure 2.0% | NR |  |
| Bausback et al., 2019 | None to mild 37.3% Moderate to moderately severe 28.0% Severe 34.6% | NR | 84 | 30.7 | 81.3 | Cerebrovascular disease 12.0 | Renal insufficiency glomerular filtration rate <60 ml/min/1.73 m^2 18.7% | Yes |  |
| Bertges et al., 2021 | NR | NR | 40 | 56 | 88 | Coronary artery disease32 Chronic heart failure 22 | Renal insufficiency (creatinine ≥ 2.0mg/dL) 5% Dialysis 9% | NR |  |
| Bianchini Massoni et al., 2023 | 0 | NR | 24.1 | 56.7 | 85.2 | Coronary artery disease  45.8 | Chronic renal failure 32% | NR |  |
| Bisdas et al., 2018 | Moderate or severe 42% | NR | 52 | 37 | 92 | Congestive heart disease 40 Cerebrovascular disease 27 | Chronic kidney disease 18% Dialysis 5% | No |  |
| Bosiers et al., 2009 | None: 55.6%  Moderate: 28.5%  Not evaluable: 15.9% | NR | NR | 45.7 | 79.5 | Angina 25.2  Arrhythmia 14.2  Congestive heart failure 8.8 | Renal insufficiency 14.7 | Yes |  |
| Bosiers et al., 2011 | 79 | C&D 100% | 71 | 27 | 61 | NR | Renal insufficiency 21 | No |  |
| Bosiers et al., 2015 | 0.333 | NR | 87.2 | 33.3 | 69.3 | NR | Renal insufficiency: 5.1 | Yes |  |
| Bosiers et al., 2023 | NR | C: 8% D: 92% | 70.8 | 27.4 | 65.5 | Coronary artery disease 23.0 Cerebrovascular disease 7.1 | Renal insufficiency 9.7 | NR |  |
| Bunte et al., 2018 | NR | NR | NR | 47.2 | 88.8 | Previous percutaneous coronary intervention 39.9 | NR | Yes |  |
| Chan et al., 2015 | 46 | Per lesion A 30 B 48 C 19 D 2 | 58 | 67 | 83 | NR | NR | No |  |
| Cheban et al., 2023 | NR | NR | NR | 49 | 90 | Chronic heart failure (I-II NYHA) 67 Heart rhythm disturbance 17 | Chronic kidney disease 79% | NR |  |
| Dake et al., 2013 | None 19.6% Little 32.7% Moderate 29.0% Severe 18.7% | NR | 88.6 | 100 | 79.7 | NR | Renal disease 11.3% | Yes |  |
| Dake et al., 2016 | None 1.7% Little 25.7% Moderate 35.3% Severe 37.3% | NR | 90.3 | 49.6 | 89 | NR | Renal disease 10.2 | Yes |  |
| Davaine et al., 2012 | NR | C 62.9% D 37.1% | 40.3 | 43.1 | 82.8 | Coronary arterial disease - 48.3 | Renal insufficiency - 22.4 | Yes |  |
| Davaine et al., 2013 | Mild - 24% Moderate - 30% Severe - 46% | C 67% D 33% | NR | 41.5 | 90.2 | Coronary arterial disease - 53.6 Chronic heart failure - 19.5 | Chronic renal failure 21.4 | Yes |  |
| Dearing et al., 2009 | NR | A 22.9% B 40.8% C 18.9% D 17.4% | NR | 43.4 | 72.6 | Coronary arterial disease 55.3 | Creatinine > 2.0 - 18% Dialysis dependent 7.2% | No |  |
| Dick et al., 2009 | NR | NR | 91 | 29 | 79 | Symptomatic coronary artery disease 35 | NR | NR |  |
| Elmahdy et al., 2017 | Severe 31.3% Moderate 35.8% | TASC II C 72.9 D 27% | 66.2 | 40.3 | 62.4 | Previous coronary percutaneous coronary intervention 48.8 Previous coronary artery bypass graft 15.9 | Renal insufficiency 29.5% | No |  |
| Falkowski et al., 2020 | NR | NR | NR | 37 | 81 | Coronary artery disease 44 | Chronic kidney disease 11.1 | No |  |
| Falkowski et al., 2020 | NR | NR | NR | 40 | 82 | Coronary artery disease 51 | Chronic kidney disease 12.3 | No |  |
| Fujihara et al., 2016 | NR | A/B 38.4% C/D 61.6% | 70 | 63.3 | 96.6 | NR | Dialysis 41.6% | Yes |  |
| Fujihara et al., 2023 | Severe calcification 86.7% | NR | NR | 66.9 | 88.6 | Coronary artery disease 56.5 Chronic heart failure 16.4 | Chronic renal failure 52.8% | Yes |  |
| Gabrielli et al., 2015 | NR | NR | NR | 53 | 80 | NR | NR | No |  |
| Gabrielli et al., 2015 | NR | NR | NR | 46 | 71 | NR | NR | No |  |
| Garcia et al., 2015 | Mild 27.3% Moderate 28.0% Severe 44.7% | TASC II  A 55.5% B 38.9% C 5.7% D 0.0% | NR | 43.5 | 93.9 | History of coronary artery disease 66.9 | Renal insufficiency 9.1% | Yes |  |
| Geraghty et al., 2013 | None - 19.4% Mild - 18.1% Moderate - 43.1% Severe - 19.4% | C&D 100% | NR | 43.1 | 87.5 | Congestive heart failure 4.2 | NR | Yes |  |
| Golzar et al., 2020 | None/Mild 28% Moderate 42% Severe 28% Unknown 2% | NR | NR | 40 | 92 | Coronary artery disease 56 | Renal insufficiency - 6% | Yes |  |
| Gostev et al., 2022 | NR | NR | NR | 19.5 | 91.6 | Coronary artery disease 81.8 Heart failure 91.6 | Renal insufficiency 35.6% | No |  |
| Gostev et al., 2023 | None 9.1% Moderate 90.9% | TASC  c 47.3 D 52.7 | Severe claudication 83.6 | 20 | 96.3 | Ischemic heart disease 94.5 Congestive heart failure 90.9 | Renal insufficiency 7.2% | No |  |
| Gouëffic et al., 2020 | NR | NR | NR | 48 | 69 | Coronary artery disease - 31 | Renal insufficiency - 9% | Yes |  |
| Gouëffic et al., 2020 | NR | NR | NR | 26 | 61 | Coronary artery disease - 40 | Renal insufficiency - 7% | Yes |  |
| Gouëffic et al., 2022 | None - 12.2% Mild - 28.3% Moderate - 26% Severe - 31.1% Unknown - 2,4% | NR | NR | 32.6 | 76 | Coronary artery disease - 36 | History of renal insufficiency - 8.2% | Yes |  |
| Gouëffic et al., 2022 | None - 13%  Mild - 31.1%  Moderate - 21.6%  Severe - 30.3%  Unknown - 4% | NR | NR | 31.9 | 78.1 | Coronary artery disease - 31.3 | History of renal insufficiency - 11.6% | Yes |  |
| Gray et al., 2018 | None/Mild - 32% Moderate - 35% Severe - 32% Unknown - 1% | NR | NR | 44 | 85 | Coronary artery disease - 45 | Renal insufficiency - 7% | Yes |  |
| Gray et al., 2018 | None/Mild - 36% Moderate - 23% Severe - 40% Unknown - 1% | NR | NR | 42 | 82 | Coronary artery disease - 51 | Renal insufficiency - 8% | Yes |  |
| Gray et al., 2022 | 0.72 | NR | NR | 44.4 | 88.7 | CAD 59.5 Peripheral vascular disease 78.6 Congestive heart failure 10.1 | Mean serum creatinine mg/dl - 1.0 | No |  |
| Guo et al., 2015 | NR | TASC II D 100% | 51.7 | 60.4 | 67.9 | Coronary artery disease 28.3 | NR | No |  |
| Guzzardi et al., 2021 | NR | TASC A 8.6% B 34.3% C 15.2% D 41.9% | Severe claudication 35.4 | 85.9 | 93.9 | NR | NR | No |  |
| Haine et al., 2019 | None - 12.1% Mild - 29.3% Moderate - 18.5% Severe - 40.1% | A - 3,7% B - 64,1% C - 25,1% D - 6,5% | NR | 49.3 | 88.4 | CAD 38.6 Cerebrovascular disease 16.4 | Chronic kidney disease (eGFR <60ml/min) - 35.4% | No |  |
| Hendriks et al., 2020 | None 17.4% Moderate 56.2% Severe 26.4% | TASC A 52.9% B 36.4% C 10.7% | NR | 26.5 | 70.1 | Coronary artery disease 26.5 | NR | No |  |
| Horie et al., 2024a | PACSS grade 4 75.5% | NR | NR | 68.6 | NR | NR | Renal failure on dialysis 53.4% | No |  |
| Horie et al., 2024a | PACSS grade 4 77.4% | NR | NR | 59.1 | NR | NR | Renal failure on dialysis 43.6% | No |  |
| Horie et al., 2024b | Severe 70.9% | NR | NR | 61.6 | 84.2 | Coronary artery disease 63.3 Chronic heart failure 28.2 | Renal failure on dialysis 29.4% | No |  |
| Hu et al., 2011 | 46.1 | TASCA 0%B 25.45%C 61.59%D 35.51% | NR | 49.28 | 61.59 | Coronary artery disease 35.51 | NR | No |  |
| Ichihashi et al., 2019 | PACSS 0, 35.7% PACSS 1, 19.6% PACSS 2, 9.1% PACSS 3, 7.8% PACSS 4, 27.8% | NR | NR | 66.8 | 85.9 | Coronary artery disease 44.5  Cerebrovascular disease 17.7 | Chronic kidney disease 55% | NR |  |
| Ichihashi et al., 2022 | NR | NR | NR | 64.5 | NR | NR | eGFR ≥60 mL/min/1.73 m^2 30.5% eGFR <60 mL/min/1.73 m^2 42.9% Dialysis dependence 26.7% | No |  |
| Ichihashi et al., 2022 | NR | NR | NR | 60.1 | NR | NR | eGFR ≥60 mL/min/1.73 m^2 48.8% eGFR <60 mL/min/1.73 m^2 34.5% Dialysis dependence 16.7% | No |  |
| Iida et al., 2009 | 0.38 | TASC II A 70% B 30% | 74 | 67 | 99 | Coronary artery disease 57 | Chronic renal failure on dialysis 22% | No |  |
| Iida et al., 2011a | 0.41 | TASC A 31% B 20% C 26% D 23% | 74 | 63 | 88 | Coronary artery disease - 55 | Chronic kidney failure on dialysis 21% | No |  |
| Iida et al., 2011b | 37 | A 30% B 22% C 25% D 23% | NR | 61 | 89 | Coronary artery disease 56 | End stage renal disease (ESRD) 20% | NR |  |
| Iida et al., 2015 | 0.65 | II class C/D 58% | NR | 69 | 85 | Cardiovascular disease 50 Cerebrovascular disease 18 | Renal insufficiency - 37% | NR |  |
| Iida et al., 2019 | None/Mild 75% Moderate 24% Severe 2% | NR | NR | 53 | 88 | Coronary artery disease 80 | Renal disease 25% | Yes |  |
| Iida et al., 2024 | None 32.4% Unilateral 25.7% Bilateral 41.9% | NR | NR | 60.8 | NR | Coronary artery disease - 51.3 | Chronic kidney disease 66.2% End stage renal disease on dialysis 28.4% | No |  |
| Ito et al., 2021a | 0.884 | TASC II A 25.1% B 47.1% C 14.8% D 13.1% | NR | 76.1 | 70.8 | Coronary artery disease 73.5 | NR | No |  |
| Ito et al., 2021a | 0.583 | TASC IIA 22.3%B 33.1%C 11.5%D 33.1% | NR | 58.1 | 69.4 | Coronary artery disease 58.3 | NR | No |  |
| Ito et al., 2021b | Arterial calcification 62.2% | NR | NR | 54.9 | NR | NR | Regular dialysis 22.7% | NR |  |
| Jeon-Slaughter et al., 2018 | Heavily calcified lesion 53.57% | NR | NR | 53.57 | 90.82 | NR | Chronic kidney disease 14.80% | Yes |  |
| Jeon-Slaughter et al., 2018 | Heavily calcified lesion 32.18% | NR | NR | 44.25 | 90.23 | NR | Chronic kidney disease 12.07% | Yes |  |
| Kang et al., 2016 | Moderate to severe calcification 27.0% | TASC A 4.8% TASC B 19.0% TASC C 15.9% TASC D 60.3% | NR | 49.2 | 93.7 | 77.8 (Previous peripheral arterial disease) | Renal disease (GFR <60) 38.1% Blood urea nitrogen (mg/dL) 25.3 Creatinine (mg/dL) 1.51 | No |  |
| Karashima et al., 2021 | PACSS classification 0 28% 1 22% 2 14% 3 12% 4 25% | NR | NR | 61 | NR | NR | Dialysis dependence 27% | No |  |
| Karpenko et al., 2022 | NR | TASC II D 100% | NR | 37 | 89 | Coronary heart disease 80 | Chronic renal failure 37% | NR |  |
| Katsuki et al., 2019 | PACSS  Grade 0: 36.2% Grade 1: 18.2% Grade 2: 16.0% Grade 3: 8.9% Grade 4: 20.7% | A: 40.6% B: 17.8% C: 28.2% D: 13.4% | NR | 53.8 | 85.5 | NR | Chronic renal failure 32.2% | NR |  |
| Katsuki et al., 2019 | PACSS Grade 0: 36.8%Grade 1: 22.1%Grade 2: 13.3%Grade 3: 10.5%Grade 4: 17.2% | A: 35.4%B: 20.7%C: 29.1%D: 14.7% | NR | 57.9 | 80.7 | NR | Chronic renal failure 27.5% | NR |  |
| Katsuki et al., 2020 | PACSS classification 0 35% 1 28% 2 2% 3 2% 4 33% | TASC II classification A 3% B 10% C 17% D 70% | NR | 43 | 85 | Coronary artery disease 38 | Haemodialysis 13% | No |  |
| Kawamura et al., 2009 | NR | TASC classification Type A+B 32% Type C+D 68% | 45 | 81 | 79 | Coronary artery disease 58 | Haemodialysis 100% | No |  |
| Kichikawa et al., 2019 | NR | NR | NR | 58.8 | 85.4 | NR | Chronic kidney disease 43.6% eGFR <60 mL/min/1.73 m^2 and/or dialysis 35.5% | NR |  |
| Kim et al., 2024 | severe calcification 28.8% | TASC classification Type A+B 53.8% Type C+D 46.2% | NR | 67.3 | 64.4 | Coronary artery disease 45.2 | Chronic kidney disease 19.2% End stage renal disease on dialysis 11.5% | No |  |
| Ko et al., 2019 | NR | TASC II B 13.6% C 19.7% D 66.7% | NR | 49.2 | 73.8 | Coronary artery disease 30.8 | Chronic kidney disease 4.6% | No |  |
| Ko et al., 2019 | NR | TASC II B 15.3% C 25.4% D 59.3% | NR | 50.8 | 79.7 | Coronary artery disease 35.6 | Chronic kidney disease 8.5% | No |  |
| Kum et al., 2021 | 60 | 40% TASC A+B 60% TASC C+D | NR | NR | NR | NR | NR | NR |  |
| Labed et al., 2021 | NR | C 45.3%D 54.7% | NR | 39.1 | 71.9 | 25 (Ischemic heart disease)6.2 (Heart failure)12.7 (Cerebrovascular accident) | Chronic renal failure/insufficiency 45.3% | NR |  |
| Laird et al., 2012 | Moderate and severe 35.3% | NR | NR | 38.1 | 83.6 | Coronary artery disease 56.0 | NR | Yes |  |
| Laird et al., 2014 | 0.91 | NR | 98.47 | 45.4 | 90.3 | Coronary artery disease 62.8 | NR | Yes |  |
| Laird et al., 2018 | None 50.3% Mild 2.1% Moderate 25.4% Severe 22.2% | NR | NR | 40.1 | 86.8 | Coronary artery disease 49.7 | NR | Yes |  |
| Laird et al., 2018 | None 42.4% Mild 0.0% Moderate 31.8% Severe 25.8% | NR | NR | 42.9 | 78.6 | Coronary artery disease 45.7 | NR | Yes |  |
| Lammer et al., 2013 | None or Mild 17% Moderate 49% Severe 28% Not applicable 6% | A 3% B >10 cm 42% C 23% D 32% | NR | 36 | 84 | Coronary heart disease 22 | Renal failure 7% | Yes |  |
| Lammer et al., 2013 | None or Mild 23% Moderate 62% Severe 15% | A 0% B >10 cm 28% C 25% D 47% | NR | 35 | 83 | Coronary heart disease 22 | Renal failure 17% | Yes |  |
| Lammer et al., 2015 | NR | TASC II C/D 100% | NR | 36 | 84 | Coronary heart disease 22 | Renal failure 7% | Yes |  |
| Lammer et al., 2015 | NR | TASC II C/D 100% | NR | 35 | 83 | Coronary heart disease 22 | Renal failure 17% | Yes |  |
| Lee et al., 2021 | None to mild 55.9% Moderate 13.7% Severe 30.4% | A/B 29.4% C/D 70.6% | 50 | 60.8 | 73.5 | Congestive heart failure 3.9 Coronary artery disease 40.2 | Chronic kidney disease 30.4% | No |  |
| Leopardi et al., 2014 | NR | C/D 47.8% | 53.6 | 30.4 | 62.3 | Coronary disease 30.4 | Renal insufficiency 14.5% | No |  |
| Lichtenberg et al., 2014 | NR | TASC A 23.6% B 24.8% C 19.4% D 32.2% | NR | 31.4 | 92.4 | NR | Renal insufficiency 12.1 | No |  |
| Lichtenberg et al., 2019 | Moderate 28.8% Severe 16.4% | C 11% D 9.6% | NR | 40 | 76.7 | Coronary artery disease 36.7 Previous cerebrovascular disease 11.7 | GFR < 30 ml/min 3.3% Haemodialysis 1.7% | No |  |
| Liistro et al., 2019 | Severe - 38% | NR | NR | 60 | 80 | Coronary artery disease 32 | Serum creatinine, mg/dl - 1.32 | No |  |
| Loureiro et al., 2024 | NR | NR | 7.6 | 65.8 | 84.8 | Coronary artery disease 22.8 | End-stage renal disease 13.9 | No |  |
| Low et al., 2022 | None 28% Mild 3% Moderate 11% Severe 56% | TASC A 15% B 31% C 26% D 22% | NR | 51 | 88 | Coronary artery disease 31 Chronic heart failure 29 | Dialysis 25% | No |  |
| Ma et al., 2022 | Severe 45% | A 10% B 29.4% C 43.3% D 17.2% | 47.1 | Grade 0 45.2% Grade 1 26.8% Grade 2 17.8% Grade 3 10.2% | Grade 0 28.0 Grade 1 42.0 Grade 2 15.3 Grade 3 14.6 | Cardiac status Grade 0 79.6 Grade 1 17.2 Grade 2 2.5 Grade 3 0.6 | Renal Status Grade 0 17.2% Grade 1 42.0% Grade 2 16.6% Grade 3 14.6% Grade 4 7.0% Grade 5 2.5% | No |  |
| Marples et al., 2022 | NR | NR | NR | 25 | 83 | NR | NR | NR |  |
| Matsumi et al., 2016b | NR | TASC A & B 62.1 TASC C&D 37.9 | NR | 67.3 | 66.4 | Previous coronary artery bypass graft surgery 19.6 | Haemodialysis 100% | No |  |
| Matsumi et al., 2016b | NR | TASC A & B 64.5 TASC C&D 35.5 | NR | 52.6 | 80.6 | Previous coronary artery bypass graft surgery 9.2 | Haemodialysis 0% | No |  |
| Matsumi et al., 2016a | None 51.4% Mild 32.0% Moderate 8.3% Severe 8.3% | TASC D 100% | NR | 51.5 | 76.5 | NR | Haemodialysis 20.6% | No |  |
| Matsumura et al., 2013 | None/mild 30% Mod 26.8% Severe 43.2% | NR | Moderate 39.4 Severe 55.7 | 42.5 | 88.2 | Angina 17.4 Congestive heart failure 9.4 | Renal insufficiency 9.8 | Yes |  |
| McQuade et al., 2010 | NR | A 20%  B 58% C 12% D 10% | NR | 35 | 75 | 0.325 | NR | Yes |  |
| Meng et al., 2018 | NR | TASC-II B - 100% | 16.7 | 70.8 | 54.2 | Coronary artery disease 58.3 | Haemodialysis - 29.2% | No |  |
| Meng et al., 2018 | NR | TASC-II B - 100% | 2.9 | 65.7 | 44.3 | Coronary artery disease 47.1 | Haemodialysis - 25.7% | No |  |
| Miura et al., 2018 | 57.7% | TASC II C/D 38.8% | NR | 56.5 | 85.9 | Coronary artery disease: 40.0 Previous heart failure: 1.2 Left ventricular dysfunction: 3.5 | Haemodialysis 22.4% | No |  |
| Miura et al., 2018 | 57.7% | TASC II C/D 37.7% | NR | 58.8 | 80 | Coronary artery disease 51.8Previous heart failure 7.1Left ventricular dysfunction 5.9 | Haemodialysis 20.0% | No |  |
| Montero-Baker et al., 2016 | NR | A 8.2 / B30.6 / C 27.9 / D 33.3 | 32.7 | 62.6 | 95.2 | Coronary artery disease 62 | Chronic renal insufficiency 28.6 | No |  |
| Mori et al., 2017 | NR | TASC-II C/D - 58% | NR | 55 | 83 | 55 | Haemodialysis - 35% | No |  |
| Mori et al., 2017 | NR | TASC-II C/D - 61% | NR | 67 | NR | NR | Haemodialysis - 37% | No |  |
| Müller-Hülsbeck et al., 2016 | None/Mild - 21% Moderate - 14% Severe - 65% | Class A - 44% Class B - 46% Class C - 10% Class D - 0% | NR | 35 | 74 | 39 | NR | Yes |  |
| Müller-Hülsbeck et al., 2017 | None/Mild - 21% Moderate - 14% Severe - 65% | Class A - 44% Class B - 46% Class C - 10% Class D - 0% | NR | 35 | 74 | 39 | NR | Yes |  |
| Myint et al., 2016 | None 13 / mild 31 / moderate 24 / severe 31 | A 15 / B 32 / C 22 / D 30 | 56 | 42 | 85 | Ischemic heart disease 67 | Renal insufficiency 12% | NR |  |
| Nakamura et al., 2018 | None/mild 47.4% Moderate 23.7% Severe 28.9% | A 48.1% B 49.4% C 2.6% | NR | 58.1 | 90.5 | Cardiac percutaneous transluminal coronary angioplasty and/or stent 35.1 Coronary artery disease 17.6 Angina 37.8 | Renal insufficiency/failure 18.9% | Yes |  |
| Nakao et al., 2024 | PACSS Classification 0 - 17% 1 - 16% 2 - 10% 3 - 17% 4 - 39% | Class A - 10% Class B - 13% Class C - 46% Class D - 31% | NR | 62 | 73 | 58 | Chronic kidney disease - 64% Haemodialysis - 40% | NR |  |
| Nanto et al., 2015 | NR | C&D 34.61% | NR | 59.55 | 83.33 | Coronary artery disease 58 | Haemodialysis 28% | NR |  |
| Oberto et al., 2017 | 0.3 | Class C/D - 39% | NR | 48 | 92.5 | NR | Chronic renal failure (requiring dialysis) - 14.6% | NR |  |
| Okuno et al., 2019 | NR | C 28.5% D 71.5% | 60 | 58.8 | 85.3 | Chronic heart failure 13.4 | End-stage renal disease (ESRD) 22.6% | NR |  |
| Palena et al., 2024 | Mild 13%Moderate 45%Severe 42% | TASC IIC 44%D 56% | NR | 61 | 91 | Coronary artery disease 47 | Dialysis 12%  Chronic renal failure 30% | No |  |
| Park et al., 2022 | Severe calcification 16.7% | TASC II B 10.4% C 75.0% D 14.6% | NR | 64.6 | 77.1 | Coronary artery disease 58.3 | Chronic kidney disease 20.8% Haemodialysis 8.3% | NR |  |
| Park et al., 2022 | Severe calcification 18.2% | TASC II B 3.6% C 76.4% D 20.0% | NR | 58.2 | 74.5 | Coronary artery disease 52.7 | Chronic kidney disease 23.6% Haemodialysis 12.7% | NR |  |
| Phair et al., 2020a | NR | NR | NR | 66.1 | NR | 21.4 | End-stage renal disease 10.7% | NR |  |
| Phair et al., 2020b | NR | Class A - 0 Class B - 59,8% Class C - 30,9% Class D - 9,3% | NR | 60.8 | 67 | NR | End-stage renal disease - 11.3% | NR |  |
| Phillips et al., 2018 | NR | A/B 44% C 56% D 0% | NR | 37 | 78 | Coronary artery disease 56 | NR | NR |  |
| Phillips et al., 2018 | NR | A/B 0% C 0% D 100% | NR | 38 | 85 | Coronary artery disease 52 | NR | NR |  |
| Powell et al., 2017 | None/Mild - 29.5% Moderate - 34.6% Severe - 35.65% | Class A - 40,5% Class B - 42,1% Class C - 13,7% Class D - 3,7% | NR | 35.1 | 79.9 | 43.5 | NR | Yes |  |
| Rammos et al., 2024 | grade 0 12.9 / g1 27.4 / g2 28.2 / g3 21,8 / g4 9.7 | NR | NR | 47.1 | 85.1 | 11.6 | Renal insufficiency 13.2 | NR |  |
| Rammos et al., 2024 | g0 19.1 / g1 30.2 / g2 23.5 / g3 12.4 / g4 14.7 | NR | NR | 33.9 | 85.6 | 9.4 | Renal insufficiency 6.8 | NR |  |
| Rastan et al., 2013 | NR | NR | NR | 36.1 | 82.4 | 42.9 | NR | NR |  |
| Salamaga et al., 2023 | None 5 / Mild 25 / Moderate 43 / Severe 27 | A 4 / B 31 / C 31 / D 34 | NR | 54.1 | 81.8 | 34 | Renal insufficiency 22% | NR |  |
| Saratzis et al., 2019 | 0.062 | TASC II 22% | NR | 52 | 75 | Ischaemic heart disease 44 | Chronic kidney disease > stage 3 45% | No |  |
| Saxon et al., 2013 | None/Mild - 39%Moderate/Severe - 61% | A 14%B 25%C 29%D 31% | NR | 33 | 87 | Coronary artery disease 49 | NR | Yes |  |
| Scheinert et al., 2011 | None 21.5 Minor 24.3 Moderate 24.3 Severe 29.9 | NR | 82.2 | 51.4 | 98.1 | Coronary artery disease 52.3 Carotid artery disease 21.5 | NR | None |  |
| Scheinert et al., 2013 | None 19.8 Mild 28.7 Moderate 20.8 Severe 30.7 | NR | 46.5 | 49.5 | 99 | Coronary artery disease 59.4 | NR | None |  |
| Schulte et al., 2012 | Massive 23.1 Moderate 40.8 Mild 25.9 None 10.3 | A 44.7% B 43.2% C 11.1% D 1.1% | 110 | DM: 35 Insulin-dependent DM: 48.1 | 68.4 | Coronary disease - 29.7 | Renal insufficiency - 8.9 | No |  |
| Shehada et al., 2022 | 76%  PACSS 1 25% PACSS 2 2% PACSS 3 35% PACSS 4 38% | NR | 73 | 35 | 84 | Congestive heart disease 39 Cerebrovascular disease 31 | End-stage renal disease 3% Chronic kidney disease 24% | No |  |
| Shehada et al., 2022 | 81%  PACSS 1 44% PACSS 2 15% PACSS 3 12% PACSS 4 29% | NR | 84 | 30 | 86 | Congestive heart disease 40 Cerebrovascular disease 25 | End-stage renal disease 0  Chronic kidney disease 21% | No |  |
| Shibata et al., 2023 | PACCS score 0 34.6% PACCS score 1 15.4% PACCS score 2 14.4% PACCS score 3 13.5% PACCS score 4 22.1% | TASC II type  A 18.3% B 24.0% C 35.6% D 22.1% | 71.2 | 56.7 | 81.7 | Coronary artery disease 28.2 Cerebrovascular disease 41.3 | Chronic kidney disease 42.3 Dialysis 20.2 | No |  |
| Shibata et al., 2023 | PACCS score 0 29.2%PACCS score 1 18.8%PACCS score 2 19.8% PACCS score 3 14.6%PACCS score 4 17.7% | TASC II type A 13.5%B 24.0%C 32.3%D 30.2% | 67.7 | 60.4 | 84.4 | Coronary artery disease 31.3Cerebrovascular disease 38.5 | Chronic kidney disease 57.3Dialysis 20.8 | No |  |
| Soga et al., 2010 | 0.34 | A 32.2% B 22.4% C 21.8% D 23.6% | 76 | 61 | 88 | Coronary artery disease 56 | Haemodialysis 20 | NR |  |
| Soga et al., 2011 | 41.6 | TASC II C/D - 50.1 | 74.2 | 61.2 | 85.6 | Coronary artery disease 53.5 | Haemodialysis 19.8 | NR |  |
| Stavroulakis et al., 2016 | Rutherford class 3 (IC) 69% Rutherford class 4 (CLI) 64% Rutherford class 5 (CLI) 69% | TASC II A/B lesions Rutherford class 3 (IC) 72% Rutherford class 4 (CLI) 64% Rutherford class 5 (CLI) 44% TASC II C/D lesions  Rutherford class 3 (IC) 28% Rutherford class 4 (CLI) 36% Rutherford class 5 (CLI) 56% | NR | 34 | 83 | Coronary heart disease 34 Cerebrovascular disease 22 | End-stage renal disease 3% Chronic kidney disease 36% | No |  |
| Stavroulakis et al., 2021 | 0.72 | NR | Asymptomatic or mild (Class I or II) 93 Severe (Class III) 5 Persistent tissue loss (Class V) 2 | 35 | 92 | Coronary heart disease 35 Cerebrovascular disease 31 | Chronic kidney disease 19% End stage renal disease 5% | NR |  |
| Steiner et al., 2016 | NR | NR | NR | 51 | 95 | Coronary artery disease 57  Cardiovascular disease 21 | Renal insufficiency (Creatinine >2.5 mg/dL) 26.0% | NR |  |
| Steiner et al., 2016 | NR | NR | NR | 53 | 91 | Coronary artery disease 45 Cardiovascular disease 15 | Renal insufficiency (Creatinine >2.5 mg/dL) 25% | NR |  |
| Stern et al., 2021 | NR | TASC classification A or B 62% C 15.2% D 22.8% | Rutherford class 2/3 70.3 | 45.3 | 93.7 | Coronary artery disease 42.2 Atrial fibrillation 10.9 | Chronic renal insufficiency 25% | No |  |
| Sullivan et al., 2021 | None 54.1% Moderate 31.1% Severe 14.8% | NR | NR | 45.4 | 90 | Previous percutaneous coronary intervention 33.6 Previous coronary artery bypass 20.7 | NR | Yes |  |
| Suzuki et al., 2016 | NR | TASC C/D - 41% | NR | 62 | 81 | Chronic heart failure 18 | Regular dialysis - 30% | NR |  |
| Suzuki et al., 2016 | NR | TASC C/D - 53% | NR | 65 | 88 | Chronic heart failure 11 | Regular dialysis - 24% | NR |  |
| Tan et al., 2022a | PACCS score 0 23.1% PACCS score 1 26.2% PACCS score 2 7.7% PACCS score 3 12.3% PACCS score 4 30.7% | TASC II  A 20%  B 23.1% C 23.1% D 33.8% | NR | 61.5 | 84.6 | Coronary artery disease 60 Cerebrovascular disease 29.2 | Chronic kidney disease 26.2% Haemodialysis 29.2% | No |  |
| Tan et al., 2022b | PACCS Grade 0 - 23% 1 - 18% 2 - 18% 3 - 10% 4 - 30% | TASC A 13% B 25% C 32% D 31% | NR | 59 | 83 | NR | eGFR ≥30 mL/min/1.73m - 62% eGFR <30 mL/min/1.73m - 8% Dialysis dependence - 30% | No |  |
| Teymen et al., 2018 | Severe calcification 49.0% | A 16.3%  B 44.9%  C 20.4%  D 18.4% | NR | 63.3 | 81.6 | Coronary artery disease 65.3  Cerebrovascular disease 6.1 | End-stage renal disease 10.2% | NR |  |
| Torsello et al., 2024 | PACSS class 3 25% PACSS class 4 26% | NR | NR | NR | NR | NR | NR | No |  |
| Treitl et al., 2017 | None 61.2% Mild 20.0% Severe 18.8% | A/ B 100% | NR | 50.6 | 23.5 | Coronary artery disease 72.9 Cerebrovascular disease 88.2 | NR | NR |  |
| Treitl et al., 2017 | None 53.7% Mild 19.4% Severe 26.9% | A/ B 100% | NR | 43.3 | 29.9 | Coronary artery disease 74.6 Cerebrovascular disease 88.1 | NR | NR |  |
| Treitl et al., 2017 | None 27.1% Mild 24.3% Severe 48.6% | A/ B 100% | NR | 55.7 | 22.9 | Coronary artery disease 60.0 Cerebrovascular disease 88.6 | NR | NR |  |
| Tsujimura et al., 2021 | PACSS grade0 41.3%1 17.3%2 8.1%3 11.1%4 22.1% | A 4,1%B 17,0%C 33,9%D 45,0% | NR | 59 | 85.2 | Coronary artery disease 48.7 | Renal failure on dialysis 25.1% | No |  |
| Tsujimura et al., 2021 | PACSS grade 0 31.6% 1 17.8% 2 7.5% 3 10,9% 4 32,2% | A 1.1% B 8,0% C 30,5% D 60,3% | NR | 59.8 | 85.1 | Coronary artery disease 59.2 | Renal failure on dialysis 19.5% | No |  |
| Van Meirvenne et al., 2023 | Mild 33% Moderate 40% Severe 27% | TASC  A 7% B 30% C 43% D 20% | 55 | 31 | 81 | NR | NR | No |  |
| Vartanian et al., 2013 | Heavy calcification 44% | A 13% B 49% C 24% D 14% | 48 | 41 | 76 | Coronary artery disease 41 Cerebrovascular accident 1 Congestive heart failure 7 | End-stage renal disease 17% Chronic renal insufficiency 18% | NR |  |
| Vartanian et al., 2013 | Heavy calcification 41% | A 25% B 19% C 13% D 43% | 67 | 41 | 81 | Coronary artery disease 38 Cerebrovascular accident 6 Congestive heart failure 3 | End-stage renal disease 6% Chronic renal insufficiency 14% | NR |  |
| Vent et al., 2017 | NR | Class C - 62.9% Class D - 37.1% | 40.3 | 43.1 | 82.8 | Coronary artery disease 48.3 Cerebrovascular disease 15.5 | Renal impairment - 22.4% | Yes |  |
| Vent et al., 2017 | NR | Class C - 58.3% Class D - 41.7% | 53.3 | 26.7 | 68.9 | Coronary artery disease 35.6 Cerebrovasuluar disease 15.6 | Renal impairment - 17.8% | Yes |  |
| Watanabe et al., 2018 | 28.3 | TASC II A 2.4 B 9.4 C 31.5 D 56.7 | NR | 44.9 | 82.7 | Previous coronary artery bypass grafting 8.7 | Chronic kidney disease 58.3 Haemodialysis 24.4 | No |  |
| Watanabe et al., 2018 | 32.4 | TASC II A 6.3 B 4.5 C 36.0 D 53.2 | NR | 54.1 | 84.7 | Previous coronary artery bypass grafting 9.0 | Chronic kidney disease 59.5 Haemodialysis 28.8 | No |  |
| Werner et al., 2013 | None - 6% Little - 46% Moderate - 39% Heavy - 9% | NR | 78 | 51 | 86 | Coronary artery disease 39  Cerebrovascular disease 12 | Renal failure - 21% | Yes |  |
| Werner et al., 2014 | Mild 30% Moderate 21.8% Severe 30.6% | NR | 53.9 | 51.3 | 94.9 | Coronary artery disease, 57 | Renal insufficiency, 26% | NR |  |
| Wittig et al., 2024 | Calcification  Grade 0 5% Grade 1 23.3% Grade 2 3.3% Grade 3 33.3% Grade 4 35.0% | TASC II B 25.0% C 46.7% D 28.3% | NR | 36.7 | 93.3 | Conary artery disease 43.3 | Renal insufficiency, 18.3% | Yes |  |
| Yang et al., 2022 | 0.409 | A 0.4% B 35% C 32.3% D 31.5% | NR | 69.1 | 76 | Coronary heart disease, 63.4 | Chronic kidney disease 15.4 | NR |  |
| Ye et al., 2022 | None - 24.2% Little - 34.8% Moderate - 31.5% Severe - 9.6% | NR | NR | 56.2 | 76.4 | NR | Renal disease - 5.6% | Yes |  |
| Ye et al., 2023 | None 10.5% Mild 54.4% Moderate 24.6% Severe 10.5% | C 29.9% D 69.7% | NR | 59.7 | 67.2 | Coronary artery disease: 22.4 | Renal dysfunction: 6.0 | No |  |
| Yokoi et al., 2016 | NR | NR | NR | 58.8 | 85.3 | NR | Chronic Kidney disease - 43.8%  eGFR<60ml/min/1.73m2 and or dialysis - 35.5% | Yes |  |
| Yoshioka et al., 2023 | PACSS grade 0 - 24.6% 1 - 33.8% 2 - 1.5% 3 - 18.5% 4 - 21.5% | NR | NR | 60 | 83.1 | NR | Dialysis - 16.9% CKD - 58.5% | NR |  |
| Zamani et al., 2021 | NR | NR | 33.3 | 59.7 | 93 | Coronary artery disease 54.4 Cerebrovascular accident 21.1 | End-stage renal disease 1.8% | NR |  |
| Zamani et al., 2021 | NR | NR | 68.9 | 44.6 | 94.6 | Coronary artery disease 51.4 Cerebrovascular accident 5.4 | End-stage renal disease 0% | NR |  |
| Zamani et al., 2021 | NR | NR | 61.1 | 59 | 88.4 | Coronary artery disease 42.1 Cerebrovascular accident 7.4 | End-stage renal disease 2.1% | NR |  |
| Zeller et al., 2014 | None 20.6%Slight 48.5%Moderate 21.6%Severe 9.3% | NR | NR | 38.1 | 80.4 | NR | End-stage renal disease 2.1%Renal insufficiency (Creatinine >1.2 mg/dl) 18.6% | NR |  |
| Zeller et al., 2016 | 0.73 | TASC II A 42% B 58% C 0% | NR | 42 | 85 | NR | Renal disease, 0% | Yes |  |
| Zeller et al., 2016 | 0.64 | TASC II A 42% B 56% C 2% | NR | 26 | 88 | NR | Renal disease, 4% | Yes |  |
| Zhao et al., 2021 | Severe calcification 23.8% | B 35.7% C 23.8% D 40.5% | NR | 53.8 | 66.7 | Coronary heart disease 35.9 | Renal insufficiency (GFR<60 mL/min/1.73 m^2) 15.4% | NR |  |

Supplementary Table 4. Downs and Black Quality Appraisal and characteristics of the included studies

| Short ID | Title | Country | Study design | Downs and Black Quality Appraisal | |
| --- | --- | --- | --- | --- | --- |
|  |  |  |  | Score | Rating |
| Abi-Khalil et al., 2022 | Comparison of Interwoven Nitinol and Drug-Eluting Stents for Endovascular Treatment of Femoropopliteal Artery Disease | Belgium | Retrospective | 17 | Fair (15-19) |
| AbuRahma et al., 2022 | Clinical Outcome of Drug-Eluted Stenting (Zilver PTX) in Patients With Femoropopliteal Occlusive Disease a Single Center Experience | USA | Retrospective | 14 | Poor (≤ 14) |
| Armstrong et al., 2014 | Nitinol self-expanding stents vs. balloon angioplasty for very long femoropopliteal lesions. | USA | Prospective | 17 | Fair (15-19) |
| Armstrong et al., 2020 | Comparative Outcomes of Supera Interwoven Nitinol vs Bare Nitinol Stents for the Treatment of Femoropopliteal Disease: Insights From the XLPAD Registry. | USA | Retrospective | 15 | Fair (15-19) |
| Astarcıoglu et al., 2017 | One-year results of primary stenting for TASC II D lesions of the superficial femoral and popliteal arteries. | Turkey | Prospective | 14 | Poor (≤ 14) |
| Bausback et al., 2019 | Drug-Eluting Stent Versus Drug-Coated Balloon Revascularization in Patients With Femoropopliteal Arterial Disease | Belgium, Germany | Prospective | 19 | Fair (15-19) |
| Bertges et al., 2021 | Vascular Quality Initiative Surveillance of Femoropopliteal Artery Paclitaxel Devices | USA | Prospective | 18 | Fair (15-19) |
| Bianchini Massoni et al., 2023 | Real-world outcomes of Cook Zilver PTX in femoro-popliteal district from multicenter experience | Italy | Retrospective | 17 | Fair (15-19) |
| Bisdas et al., 2018 | 1-Year All-Comers Analysis of the Eluvia Drug-Eluting Stent for Long Femoropopliteal Lesions After Suboptimal Angioplasty | Germany | Retrospective | 14 | Poor (≤ 14) |
| Bosiers et al., 2009 | Nitinol stent implantation in long superficial femoral artery lesions: 12-month results of the DURABILITY I study. | Belgium | Prospective | 13 | Poor (≤ 14) |
| Bosiers et al., 2011 | Results of the Protégé EverFlex 200-mm-long nitinol stent (ev3) in TASC C and D femoropopliteal lesions. | Belgium | Prospective | 14 | Poor (≤ 14) |
| Bosiers et al., 2015 | Superiority of stent-grafts for in-stent restenosis in the superficial femoral artery: twelve-month results from a multicenter randomized trial | Belgium, Germany | Prospective | 23 | Good (20-24) |
| Bosiers et al., 2023 | ZILVERPASS Study: ZILVER PTX Stent versus Prosthetic Above-the-Knee Bypass Surgery in Femoropopliteal Lesions, 5-year Results | Brazil, Belgium, Germany, Italy | Prospective | 18 | Fair (15-19) |
| Bunte et al., 2018 | Long-term clinical and quality of life outcomes after stenting of femoropopliteal artery stenosis: 3-year results from the STROLL study. | USA | Prospective | 14 | Poor (≤ 14) |
| Chan et al., 2015 | Predictors of restenosis in the use of helical interwoven nitinol stents to treat femoropopliteal occlusive disease. | China | Prospective | 14 | Poor (≤ 14) |
| Cheban et al., 2023 | One-Year Results of Long femoropopliteal Lesions Stenting with Fasciotomy Lamina Vastoadductoria | Russia | Prospective | 21 | Good (20-24) |
| Dake et al., 2013 | Sustained safety and effectiveness of paclitaxel-eluting stents for femoropopliteal lesions: 2-year follow-up from the Zilver PTX randomized and single-arm clinical studies | Germany, Japan, USA | Prospective | 15 | Fair (15-19) |
| Dake et al., 2016 | Durable Clinical Effectiveness With Paclitaxel-Eluting Stents in the Femoropopliteal Artery: 5-Year Results of the Zilver PTX Randomized Trial | USA, Japan, Germany | Prospective | 20 | Good (20-24) |
| Davaine et al., 2012 | One-year clinical outcome after primary stenting for Trans-Atlantic Inter-Society Consensus (TASC) C and D femoropopliteal lesions (the STELLA "STEnting Long de L'Artère fémorale superficielle" cohort). | France | Prospective | 16 | Fair (15-19) |
| Davaine et al., 2013 | Incidence and the clinical impact of stent fractures after primary stenting for TASC C and D femoropopliteal lesions at 1 year. | France | Prospective | 16 | Fair (15-19) |
| Dearing et al., 2009 | Primary stenting of the superficial femoral and popliteal artery. | USA | Prospective | 13 | Poor (≤ 14) |
| Dick et al., 2009 | Balloon angioplasty versus stenting with nitinol stents in intermediate length superficial femoral artery lesions. | Austria | Prospective | 23 | Good (20-24) |
| Elmahdy et al., 2017 | Long-Term Primary Patency Rate After Nitinol Self-Expandable Stents Implantation in Long, Totally Occluded Femoropopliteal (TASC II C & D) Lesions. | Italy, Egypt | Retrospective | 14 | Poor (≤ 14) |
| Falkowski et al., 2020 | Assessment of Mortality and Factors Affecting Outcome of Use of Paclitaxel-Coated Stents and Bare Metal Stents in Femoropopliteal PAD | Poland | Prospective | 20 | Good (20-24) |
| Fujihara et al., 2016 | Outcomes of Zilver PTX stent implantation for the treatment of complex femoropopliteal artery disease | Japan | Retrospective | 15 | Fair (15-19) |
| Fujihara et al., 2023 | Endovascular Therapy with Interwoven Nitinol Stent Placement after Predilation for Heavily Calcified Femoropopliteal Artery Disease: Results of the BURDOCK Study. | Japan | Prospective | 15 | Fair (15-19) |
| Gabrielli et al., 2015 | First clinical experience with the Innova versus the Protege EverFlex self-expanding bare metal stents in superficial femoral artery occlusions | Italy | Retrospective | 18 | Fair (15-19) |
| Garcia et al., 2015 | Wire-Interwoven Nitinol Stent Outcome in the Superficial Femoral and Proximal Popliteal Arteries: Twelve-Month Results of the SUPERB Trial. | USA | Prospective | 15 | Fair (15-19) |
| Geraghty et al., 2013 | Three-year results of the VIBRANT trial of VIABAHN endoprosthesis versus bare nitinol stent implantation for complex superficial femoral artery occlusive disease. | USA | Prospective | 22 | Good (20-24) |
| Golzar et al., 2020 | Effectiveness and Safety of a Paclitaxel-Eluting Stent for Superficial Femoral Artery Lesions up to 190 mm: One-Year Outcomes of the Single-Arm IMPERIAL Long Lesion Substudy of the Eluvia Drug-Eluting Stent | Austria, Belgium, Japan, New Zealand, USA | Prospective | 14 | Poor (≤ 14) |
| Gostev et al., 2022 | Bypass Versus Interwoven Nitinol Stents for Long Femoro-Popliteal Occlusions: A Propensity Matched Analysis. | Russia | Retrospective | 18 | Fair (15-19) |
| Gostev et al., 2023 | Treatment of Long Femoropopliteal Occlusive Lesions With Self-expanding Interwoven Nitinol Stent: 24 Month Outcomes of the STELLA-SUPERA-SIBERIA Register Trial. | Russia | Prospective | 15 | Fair (15-19) |
| Gouëffic et al., 2020 | A Polymer-Free Paclitaxel-Eluting Stent Versus a Bare-Metal Stent for De Novo Femoropopliteal Lesions: The BATTLE Trial | France | Prospective | 23 | Good (20-24) |
| Gouëffic et al., 2022 | Efficacy of a Drug-Eluting Stent Versus Bare Metal Stents for Symptomatic Femoropopliteal Peripheral Artery Disease: Primary Results of the EMINENT Randomized Trial | Austria, Belgium, France, Germany, Ireland, Italy, Spain, Switzerland, The Netherlands, UK | Prospective | 25 | Excellent (25-27) |
| Gray et al., 2018 | A polymer-coated, paclitaxel-eluting stent (Eluvia) versus a polymer-free, paclitaxel-coated stent (Zilver PTX) for endovascular femoropopliteal intervention (IMPERIAL): a randomised, non-inferiority trial | Austria, Belgium, Canada, Germany, Japan, New Zealand, USA | Prospective | 26 | Excellent (25-27) |
| Gray et al., 2022 | Evaluation of safety and efficacy of the S.M.A.R.T.Â® Flex Vascular Stent System (OPEN study) | USA, Belgium | Prospective | 15 | Fair (15-19) |
| Guo et al., 2015 | Outcomes of endovascular treatment for patients with TASC II D femoropopliteal occlusive disease: a single center study. | China | Retrospective | 15 | Fair (15-19) |
| Guzzardi et al., 2021 | Endovascular treatment of femoro-popliteal disease with the Supera stent: results of a multicenter study. | Italy | Retrospective | 14 | Poor (≤ 14) |
| Haine et al., 2019 | Comparison Between Interwoven Nitinol and Drug Eluting Stents for Endovascular Treatment of Femoropopliteal Artery Disease | Switzerland | Retrospective | 17 | Fair (15-19) |
| Hendriks et al., 2020 | Endovascular treatment of atherosclerotic lesions in the superficial femoral artery and proximal popliteal artery using the sinus-SuperFlex-635 stent: twelve-month results from the HERO Registry. | Belgium | Prospective | 15 | Fair (15-19) |
| Horie et al., 2024a | Retrospective Multicenter Comparison Between Viabahn Covered Stent-Grafts and Supera Interwoven Nitinol Stents for Endovascular Treatment in Severely Calcified Femoropopliteal Artery Disease: The ARMADILLO Study (Adjusted Retrospective coMparison of scAffolDs In caLcified LesiOns). | Japan | Retrospective | 19 | Fair (15-19) |
| Horie et al., 2024b | Multicenter Registry of Common Femoral Artery Disease Treated With Endovascular Revascularization Using Interwoven Nitinol Stents: An Observational Retrospective Study. | Japan | Retrospective | 18 | Fair (15-19) |
| Hu et al., 2011 | Endovascular nitinol stenting for long occlusive disease of the superficial femoral artery in critical limb ischemia: a single-center, mid-term result. | China | Retrospective | 14 | Poor (≤ 14) |
| Ichihashi et al., 2019 | Vessel Calcification as a Risk Factor for In-Stent Restenosis in Complex Femoropopliteal Lesions After Zilver PTX Paclitaxel-Coated Stent Placement | Japan | Retrospective | 13 | Poor (≤ 14) |
| Ichihashi et al., 2022 | Drug Eluting Versus Covered Stent for Femoropopliteal Artery Lesions: Results of the ULTIMATE Study | Japan | Retrospective | 18 | Fair (15-19) |
| Iida et al., 2009 | Long-term results of endovascular therapy with nitinol stent implantation for TASC II A/B femoro-popliteal artery lesions: 4 years' experience. | Japan | Retrospective | 19 | Poor (≤ 14) |
| Iida et al., 2011a | Long-term outcomes and risk stratification of patency following nitinol stenting in the femoropopliteal segment: retrospective multicenter analysis. | Japan | Retrospective | 16 | Fair (15-19) |
| Iida et al., 2011b | Timing of the restenosis following nitinol stenting in the superficial femoral artery and the factors associated with early and late restenoses. | Japan | Retrospective | 11 | Poor (≤ 14) |
| Iida et al., 2015 | 1-Year Results of the ZEPHYR Registry (Zilver PTX for the Femoral Artery and Proximal Popliteal Artery): Predictors of Restenosis | Japan | Prospective | 13 | Poor (≤ 14) |
| Iida et al., 2019 | Self-Expanding Nitinol Stent vs Percutaneous Transluminal Angioplasty in the Treatment of Femoropopliteal Lesions: 3-Year Data From the SM-01 Trial. | Japan | Prospective | 24 | Good (20-24) |
| Iida et al., 2024 | Three-year clinical course after fluoropolymer-based drug-eluting stent implantation for femoropopliteal lesions. | Japan | Prospective | 12 | Poor (≤ 14) |
| Ito et al., 2021a | Long-term clinical outcomes after self-expandable bare nitinol stent implantation for femoropopliteal occlusive disease in hemodialysis patients. | Japan | Retrospective | 18 | Fair (15-19) |
| Ito et al., 2021b | Lumen Loss at 1 Year After Bare Nitinol Stent Implantation in the Superficial Femoral Artery | Japan | Retrospective | 15 | Fair (15-19) |
| Jeon-Slaughter et al., 2018 | Comparative Effectiveness Study of Drug-Eluting and Bare-Metal Peripheral Artery Stents in Endovascular Femoropopliteal Artery Revascularization | USA | Retrospective | 18 | Fair (15-19) |
| Kang et al., 2016 | A single center experience of Zilver PTX for femoro-popliteal lesions | USA | Retrospective | 13 | Poor (≤ 14) |
| Karashima et al., 2021 | Three-Year Clinical Outcomes of the Innova™ Self-Expanding Nitinol Stent for the Treatment of Femoropopliteal Lesions. | Japan | Retrospective | 14 | Poor (≤ 14) |
| Karpenko et al., 2022 | Influence of lamina vastoadductoria dissection on the outcomes of femoral artery extensive lesion stenting: A pilot randomised investigation | Russia | Prospective | 21 | Good (20-24) |
| Katsuki et al., 2019 | Mortality Risk Following Application of a Paclitaxel-Coated Stent in Femoropopliteal Lesions | Japan | Retrospective | 17 | Fair (15-19) |
| Katsuki et al., 2020 | Combination therapy of heparin-bonded covered stent and bare-nitinol stent assessed by intravascular ultrasound. | Japan | Retrospective | 14 | Poor (≤ 14) |
| Kawamura et al., 2009 | Nitinol stenting improves primary patency of the superficial femoral artery after percutaneous transluminal angioplasty in hemodialysis patients: a propensity-matched analysis. | Japan | Retrospective | 17 | Fair (15-19) |
| Kichikawa et al., 2019 | Zilver PTX Post-market Surveillance Study of Paclitaxel-Eluting Stents for Treating Femoropopliteal Artery Disease in Japan: 2-Year Results | Japan | Prospective | 16 | Fair (15-19) |
| Kim et al., 2024 | Korean Multicenter Registry of ELUVIA Stent for Femoropopliteal Artery Disease: K-ELUVIA Registry. | South Korea | Prospective | 15 | Fair (15-19) |
| Ko et al., 2019 | Comparison of Spot versus Long Stenting for Femoropopliteal Artery Disease. | South Korea | Prospective | 21 | Good (20-24) |
| Kum et al., 2021 | Outcomes of the paclitaxel-eluting Eluvia stent for long femoropopliteal lesions in Asian patients with predominantly chronic limb-threatening ischemia | Singapore | Retrospective | 13 | Poor (≤ 14) |
| Labed et al., 2021 | Endovascular Treatment of Long Femoropopliteal Lesions with Contiguous Bare Metal Stents | France | Retrospective | 13 | Poor (≤ 14) |
| Laird et al., 2012 | Nitinol stent implantation vs. balloon angioplasty for lesions in the superficial femoral and proximal popliteal arteries of patients with claudication: three-year follow-up from the RESILIENT randomized trial. | USA, Europe | Prospective | 20 | Good (20-24) |
| Laird et al., 2014 | Nitinol stent implantation in the superficial femoral artery and proximal popliteal artery: twelve-month results from the complete SE multicenter trial. | USA, Belgium, Germany | Prospective | 14 | Poor (≤ 14) |
| Laird et al., 2018 | Novel Nitinol Stent for Lesions up to 24 cm in the Superficial Femoral and Proximal Popliteal Arteries: 24-Month Results From the TIGRIS Randomized Trial. | USA, Europe | Prospective | 20 | Good (20-24) |
| Lammer et al., 2013 | Heparin-bonded covered stents versus bare-metal stents for complex femoropopliteal artery lesions: the randomized VIASTAR trial (Viabahn endoprosthesis with PROPATEN bioactive surface VIA versus bare nitinol stent in the treatment of long lesions in superficial femoral artery occlusive disease) | Austria, Germany | Prospective | 23 | Good (20-24) |
| Lammer et al., 2015 | Sustained benefit at 2 years for covered stents versus bare-metal stents in long SFA lesions: the VIASTAR trial | Austria, Germany | Prospective | 23 | Good (20-24) |
| Lee et al., 2021 | Drug Eluting Stent vs. Drug Coated Balloon for Native Femoropopliteal Artery Disease: A Two Centre Experience | Korea | Retrospective | 18 | Fair (15-19) |
| Leopardi et al., 2014 | Effectiveness of Zilver PTX eluting stent in TASC C/D lesions and restenosis | France | Prospective | 14 | Poor (≤ 14) |
| Lichtenberg et al., 2014 | PEACE I all-comers registry: patency evaluation after implantation of the 4-French Pulsar-18 self-expanding nitinol stent in femoropopliteal lesions. | Germany | Prospective | 16 | Fair (15-19) |
| Lichtenberg et al., 2019 | Effectiveness of the Pulsar-18 self-expanding stent with optional drug-coated balloon angioplasty in the treatment of femoropopliteal lesions - the BIOFLEX PEACE All-Comers Registry | Germany | Prospective | 13 | Poor (≤ 14) |
| Liistro et al., 2019 | Drug-Eluting Balloon Versus Drug-Eluting Stent for Complex Femoropopliteal Arterial Lesions: The DRASTICO Study | Italy | Prospective | 21 | Good (20-24) |
| Loureiro et al., 2024 | Navigating complexity: The Supera's triumph in femoropopliteal lesions. | Portugal | Retrospective | 14 | Poor (≤ 14) |
| Low et al., 2022 | Midterm Results of the Supera Stent for the Treatment of Femoropopliteal Occlusive Disease. | USA | Retrospective | 10 | Poor (≤ 14) |
| Ma et al., 2022 | Mid-term Efficacy and Safety of Drug-coated Balloon versus Nitinol Bare Metal Stent for Primary Lesions in Femoropopliteal Artery Disease | China | Retrospective | 17 | Fair (15-19) |
| Marples et al., 2022 | Prophylactic paclitaxel-eluting stent placement does not improve covered femoropopliteal stent patency | Australia | Retrospective | 15 | Fair (15-19) |
| Matsumi et al., 2016b | Long-term risks for patency loss in patients with hemodialysis after bare self-expandable nitinol stent implantation to femoropopliteal artery occlusive lesions. | Japan | Retrospective | 16 | Fair (15-19) |
| Matsumi et al., 2016a | Long-Term Outcomes of Self-Expandable Nitinol Stent Implantation With Intraluminal Angioplasty to Treat Chronic Total Occlusion in the Superficial Femoral Artery (TransAtlantic Inter-Society Consensus Type D Lesions). | Japan | Retrospective | 14 | Fair (15-19) |
| Matsumura et al., 2013 | The United States StuDy for EvalUating EndovasculaR TreAtments of Lesions in the Superficial Femoral Artery and Proximal Popliteal By usIng the Protégé EverfLex NitInol STent SYstem II (DURABILITY II). | USA and Europe | Prospective | 17 | Fair (15-19) |
| McQuade et al., 2010 | Four-year randomized prospective comparison of percutaneous ePTFE/nitinol self-expanding stent graft versus prosthetic femoral-popliteal bypass in the treatment of superficial femoral artery occlusive disease. | USA | Prospective | 16 | Fair (15-19) |
| Meng et al., 2018 | Real-World Comparison of Drug-Eluting and Bare-Metal Stents in Superficial Femoral Artery Occlusive Disease with Trans-Atlantic Intersociety Consensus B Lesions: A 2-Year, Single-Institute Study | Taiwan | Retrospective | 14 | Poor (≤ 14) |
| Miura et al., 2018 | Drug-Eluting Versus Bare-Metal Stent Implantation With or Without Cilostazol in the Treatment of the Superficial Femoral Artery | Japan | Prospective | 24 | Good (20-24) |
| Montero-Baker et al., 2016 | Analysis of endovascular therapy for femoropopliteal disease with the Supera stent. | USA | Prospective | 11 | Poor (≤ 14) |
| Mori et al., 2017 | Penetration rate of the placement of a drug-eluting stent for the treatment of superficial femoral artery lesions in Japan | Japan | Prospective | 16 | Fair (15-19) |
| Müller-Hülsbeck et al., 2016 | Twelve-Month Results From the MAJESTIC Trial of the Eluvia Paclitaxel-Eluting Stent for Treatment of Obstructive Femoropopliteal Disease | Europe, Australia, New Zealand | Prospective | 15 | Fair (15-19) |
| Müller-Hülsbeck et al., 2017 | Long-Term Results from the MAJESTIC Trial of the Eluvia Paclitaxel-Eluting Stent for Femoropopliteal Treatment: 3-Year Follow-up | Europe, Australia, New Zealand | Prospective | 16 | Fair (15-19) |
| Myint et al., 2016 | A Real-World Experience With the Supera Interwoven Nitinol Stent in Femoropopliteal Arteries: Midterm Patency Results and Failure Analysis. | Australia, Netherlands | Retrospective | 13 | Poor (≤ 14) |
| Nakamura et al., 2018 | Nitinol Self-Expanding Stents for the Treatment of Obstructive Superficial Femoral Artery Disease: Three-Year Results of the RELIABLE Japanese Multicenter Study. | Japan | Prospective | 16 | Fair (15-19) |
| Nakao et al., 2024 | Impact of Procedural Techniques on Midterm Patency of Fluoropolymer-Based Drug-Eluting Stent Placed in the Femoropopliteal Artery | Japan | Retrospective | 14 | Poor (≤ 14) |
| Nanto et al., 2015 | Effect of Cilostazol Following Endovascular Intervention for Peripheral Artery Disease. | Japan | Retrospective | 17 | Fair (15-19) |
| Oberto et al., 2017 | Comparison of SFA lesion treatment with Zilver PTX in diabetics vs. non-diabetics: 2-year clinical and functional results | Italy | Prospective | 13 | Poor (≤ 14) |
| Okuno et al., 2019 | Comparison of Clinical Outcomes between Endovascular Therapy with Self-Expandable Nitinol Stent and Femoral-Popliteal Bypass for Trans-Atlantic Inter-Society Consensus II C and D Femoropopliteal Lesions. | Japan | Retrospective | 15 | Fair (15-19) |
| Palena et al., 2024 | A multicenter prospective observational study appraising the effectiveness of the Supera stent after subintimal recanalization of femoro-popliteal artery occlusion: The SUPERSUB II study. | Italy | Prospective | 14 | Poor (≤ 14) |
| Park et al., 2022 | Long coverage with drug-eluting stents is superior to spot coverage for long femoropopliteal artery disease: PARADE II study | Korea | Prospective | 19 | Fair (15-19) |
| Phair et al., 2020a | Amputation-free Survival in Patients with Critical Limb Ischemia Treated with Paclitaxel-eluting Stents and Paclitaxel-coated Balloons | USA | Retrospective | 14 | Poor (≤ 14) |
| Phair et al., 2020b | Primary Patency of Long-Segment Femoropopliteal Artery Lesions in Patients with Peripheral Arterial Occlusive Disease Treated with Paclitaxel-Eluting Technology | USA | Retrospective | 14 | Poor (≤ 14) |
| Phillips et al., 2018 | Full Drug-Eluting Stent Jacket: Two-Year Results of a Single-Center Experience With Zilver PTX Stenting for Long Lesions in the Femoropopliteal Arteries | USA | Retrospective | 15 | Fair (15-19) |
| Powell et al., 2017 | Stent placement in the superficial femoral and proximal popliteal arteries with the innova self-expanding bare metal stent system | Europe, USA, Canada, Japan | Prospective | 18 | Fair (15-19) |
| Rammos et al., 2024 | The BioMimics 3D Helical Centreline Nitinol Stent in Chronic Limb Threatening Ischaemia and Complex Lesions: Three Year Outcomes of the MIMICS-3D Registry. | Europe | Prospective | 16 | Fair (15-19) |
| Rastan et al., 2013 | Stent placement versus balloon angioplasty for the treatment of obstructive lesions of the popliteal artery: a prospective, multicenter, randomized trial. | Germany, Austria, Switzerland | Prospective | 23 | Good (20-24) |
| Salamaga et al., 2023 | Three-Year Real-World Outcomes of Interwoven Nitinol Supera Stent Implantation in Long and Complex Femoropopliteal Lesions. | Poland | Retrospective | 13 | Poor (≤ 14) |
| Saratzis et al., 2019 | Interwoven Nitinol Stents versus Drug Eluting Stents in the Femoro-Popliteal Segment: A Propensity Matched Analysis | United Kingdom | Prospective | 18 | Fair (15-19) |
| Saxon et al., 2013 | Heparin-bonded, expanded polytetrafluoroethylene-lined stent graft in the treatment of femoropopliteal artery disease: 1-year results of the VIPER (Viabahn Endoprosthesis with Heparin Bioactive Surface in the Treatment of Superficial Femoral Artery Obstructive Disease) trial | USA | Prospective | 14 | Poor (≤ 14) |
| Scheinert et al., 2011 | A novel self-expanding interwoven nitinol stent for complex femoropopliteal lesions: 24-month results of the SUPERA SFA registry. | Germany | Retrospective | 14 | Poor (≤ 14) |
| Scheinert et al., 2013 | Treatment of complex atherosclerotic popliteal artery disease with a new self-expanding interwoven nitinol stent: 12-month results of the Leipzig SUPERA popliteal artery stent registry. | Germany | Retrospective | 15 | Fair (15-19) |
| Schulte et al., 2012 | MISAGO 2: one-year outcomes after implantation of the Misago self-expanding nitinol stent in the superficial femoral and popliteal arteries of 744 patients. | USA | Prospective | 16 | Fair (15-19) |
| Shehada et al., 2022 | Efficacy analysis following polymer coated drug eluting stent and bare metal stent deployment for femoropopliteal arterial disease | Germany | Retrospective | 16 | Fair (15-19) |
| Shibata et al., 2023 | One Year Outcomes of Zilver PTX Versus Eluvia for Femoropopliteal Disease in Real-World Practice: REALDES Study | Japan | Prospective | 16 | Fair (15-19) |
| Soga et al., 2010 | Mid-term clinical outcome and predictors of vessel patency after femoropopliteal stenting with self-expandable nitinol stent. | Japan | Retrospective | 18 | Fair (15-19) |
| Soga et al., 2011 | Utility of new classification based on clinical and lesional factors after self-expandable nitinol stenting in the superficial femoral artery. | Japan | Retrospective | 14 | Poor (≤ 14) |
| Stavroulakis et al., 2016 | Results of primary stent therapy for femoropopliteal peripheral arterial disease at 7 years | Germany | Prospective | 12 | Poor (≤ 14) |
| Stavroulakis et al., 2021 | 2-Year Outcomes of the Eluvia Drug-Eluting Stent for the Treatment of Complex Femoropopliteal Lesions | Greece | Retrospective | 14 | Poor (≤ 14) |
| Steiner et al., 2016 | Midterm Patency After Femoropopliteal Interventions: A Comparison of Standard and Interwoven Nitinol Stents and Drug-Coated Balloons in a Single-Center, Propensity Score-Matched Analysis | Germany | Retrospective | 16 | Fair (15-19) |
| Stern et al., 2021 | Paclitaxel exposure and long-term mortality of patients treated with the Zilver PTX drug-eluting stent | USA | Retrospective | 12 | Poor (≤ 14) |
| Sullivan et al., 2021 | Treatment of Femoropopliteal Lesions With the BioMimics 3D Vascular Stent System: Two-Year Results From the MIMICS-2 Trial | USA, Germany, Japan | Prospective | 16 | Poor (≤ 14) |
| Suzuki et al., 2016 | Retrospective Multicenter Comparison of S.M.A.R.T. CONTROL and MISAGO Stents in Treatment of Femoropopliteal Lesions. | Japan | Retrospective | 16 | Fair (15-19) |
| Tan et al., 2022a | Mortality risk after use of a paclitaxel-coated stent in femoropopliteal peripheral artery disease | Japan | Retrospective | 17 | Fair (15-19) |
| Tan et al., 2022b | Three-Year Clinical Outcomes Following Implantation of LifeStent Self-Expanding Nitinol Stents in Patients With Femoropopliteal Artery Lesions. | Japan | Retrospective | 16 | Fair (15-19) |
| Teymen et al., 2018 | Comparison of drug-eluting balloon angioplasty with self-expanding interwoven nitinol stent deployment in patients with complex femoropopliteal lesions | Turkey | Retrospective | 16 | Fair (15-19) |
| Torsello et al., 2024 | Treatment of Femoropopliteal Artery Disease with Polymer-Coated Drug-Eluting Stent: 5-Year Results of a Prospective, Non-Randomized Study Including the Halo Phenomenon | Germany | Prospective | 14 | Poor (≤ 14) |
| Treitl et al., 2017 | Evolution of patency rates of self-expandable bare metal stents for endovascular treatment of femoro-popliteal arterial occlusive disease: Does stent design matter? | Germany | Retrospective | 17 | Fair (15-19) |
| Tsujimura et al., 2021 | Clinical outcomes of polymer-free, paclitaxel-coated stents vs stent grafts in peripheral arterial disease patients with femoropopliteal artery lesions | Japan | Retrospective | 17 | Fair (15-19) |
| Van Meirvenne et al., 2023 | Self-expanding interwoven nitinol stent in severe femoropopliteal arterial disease. Real life results of the Supera Peripheral Stent System(®). | Belgium | Retrospective | 14 | Poor (≤ 14) |
| Vartanian et al., 2013 | Clinical consequence of bare metal stent and stent graft failure in femoropopliteal occlusive disease | USA | Retrospective | 16 | Fair (15-19) |
| Vent et al., 2017 | Bare Metal Versus Paclitaxel-Eluting Stents for Long Femoropopliteal Lesions: Prospective Cohorts Comparison Using a Propensity Score-Matched Analysis | France | Prospective | 18 | Fair (15-19) |
| Watanabe et al., 2018 | The Importance of Patency of Tibial Run Off Arteries on Clinical Outcomes After Stenting for Chronic Total Occlusions in the Superficial Femoro-popliteal Artery. | Japan | Retrospective | 18 | Fair (15-19) |
| Werner et al., 2013 | SUMMIT registry: one-year outcomes after implantation of the EPIC self-expanding nitinol stent in the femoropopliteal segment | Germany | Prospective | 15 | Fair (15-19) |
| Werner et al., 2014 | Treatment of complex atherosclerotic femoropopliteal artery disease with a self-expanding interwoven nitinol stent: midterm results from the Leipzig SUPERA 500 registry. | Germany | Prospective | 14 | Poor (≤ 14) |
| Wittig et al., 2024 | Randomized Trial Comparing a Stent-Avoiding With a Stent-Preferred Strategy in Complex Femoropopliteal Lesions. | Germany | Prospective | 20 | Good (20-24) |
| Yang et al., 2022 | Treatment of Atherosclerotic Femoropopliteal Artery Disease with Supera Interwoven Nitinol Stent: A Real-World Study in China. | China | Retrospective | 13 | Poor (≤ 14) |
| Ye et al., 2022 | First peripheral drug-eluting stent clinical results from China: 1-year outcomes of the Zilver PTX China study | China | Prospective | 17 | Fair (15-19) |
| Ye et al., 2023 | Stent Graft vs Drug-Coated Balloon in Endovascular Treatment of Complex Femoropopliteal Artery Lesions: A 2-Center Experience | China | Retrospective | 18 | Fair (15-19) |
| Yokoi et al., 2016 | Zilver PTX Post-Market Surveillance Study of Paclitaxel-Eluting Stents for Treating Femoropopliteal Artery Disease in Japan: 12-Month Results | Japan | Prospective | 18 | Fair (15-19) |
| Yoshioka et al., 2023 | Two-year clinical outcomes and predictors of restenosis following the use of polymer-coated paclitaxel-eluting stents or drug-coated balloons in patients with femoropopliteal artery disease | Japan | Retrospective | 16 | Fair (15-19) |
| Zamani et al., 2021 | Outcomes after Endovascular Stent Placement for Long-Segment Superficial Femoral Artery Lesions | USA | Retrospective | 17 | Fair (15-19) |
| Zeller et al., 2014 | Drug-coated balloons vs. drug-eluting stents for treatment of long femoropopliteal lesions | Germany | Retrospective | 18 | Fair (15-19) |
| Zeller et al., 2016 | Helical Centerline Stent Improves Patency: Two-Year Results From the Randomized Mimics Trial. | Germany | Prospective | 18 | Fair (15-19) |
| Zhao et al., 2021 | Drug-Coated Balloon versus Bare Nitinol Stent in Femoropopliteal Artery: 12 Months Outcome from a Single Center in China | China | Retrospective | 18 | Fair (15-19) |

Supplementary Table 5. Primary patency at 12 and 24 months post stenting based on the use of core laboratory adjudication*

| Stent type | 12 months | | | | 24 months | | | |
| --- | --- | --- | --- | --- | --- | --- | --- | --- |
|  | Data points (n) | Popula-tion (n) | Propor-tion (%) | 95% CI | Data points (n) | Popula-tion (n) | Propor-tion (%) | 95% CI |
| Eluvia™ | 5 | 838 | 85.39 | 79.37;89.88 | 1 | 53 | 83.02 | 70.47;90.92 |
| Zilver® PTX® | 8 | 1732 | 76.87 | 65.49;85.33 | 3 | 397 | 71.23 | 44.79;88.31 |
| Viabahn | 6 | 403 | 75.19 | 69.26;80.3 | 3 | 184 | 64.13 | 47.99;77.6 |
| BMS | 24 | 3306 | 74.54 | 70.24;78.42 | 12 | 1650 | 67.21 | 58.53;74.86 |

*Only studies that reported using core laboratory adjudication were included in this analysis. Abbreviations: CI, confidence interval.

Supplementary Table 6. Primary patency at 12 and 24 months post stenting based on the Downs and Black Quality Appraisal score*

| Stent type | 12 months | | | | 24 months | | | |
| --- | --- | --- | --- | --- | --- | --- | --- | --- |
|  | Data points (n) | Popula-tion (n) | Propor-tion (%) | 95% CI | Data points (n) | Popula-tion (n) | Propor-tion (%) | 95% CI |
| Eluvia™ | 10 | 1372 | 85.86 | 82.91; 88.37 | 5 | 508 | 76.97 | 71.38; 81.74 |
| Zilver® PTX® | 24 | 3774 | 76.76 | 72.16; 80.8 | 13 | 1929 | 65.55 | 59.65; 71 |
| Viabahn | 11 | 969 | 76.28 | 71.64; 80.38 | 9 | 860 | 66.75 | 59.56; 73.23 |
| BMS | 65 | 13239 | 74.07 | 70.84; 77.06 | 38 | 8014 | 63.43 | 57.46; 69.02 |

* Only studies with a rating of fair, good, or excellent were included in this analysis. Abbreviations: CI, confidence interval.

Supplementary Table 7. Target lesions revascularisation at 12 and 24 months post stenting based on the use of core laboratory adjudication*

| Stent type | 12 months | | | | 24 months | | | |
| --- | --- | --- | --- | --- | --- | --- | --- | --- |
|  | Data points (n) | Popula-tion (n) | Propor-tion (%) | 95% CI | Data points (n) | Popula-tion (n) | Propor-tion (%) | 95% CI |
| Eluvia™ | 5 | 920 | 6.19 | 3.5; 10.71 | 1 | 52 | 7.69 | 2.92; 18.77 |
| Zilver® PTX® | 10 | 2696 | 11.00 | 6.98; 16.92 | 4 | 1184 | 16.72 | 11.55; 23.6 |
| Viabahn | 2 | 96 | 16.67 | 0.61; 86.65 | 1 | 48 | 20.83 | 11.59; 34.56 |
| BMS | 24 | 4135 | 12.73 | 10.99; 14.69 | 14 | 1916 | 19.56 | 15.43; 24.46 |

*Only studies that reported using core laboratory adjudication were included in this analysis. Abbreviations: CI, confidence interval.

Supplementary Table 8. Target lesion revascularisation at 12 and 24 months post stenting based on the Downs and Black Quality Appraisal score*

| Stent type | 12 months | | | | 24 months | | | |
| --- | --- | --- | --- | --- | --- | --- | --- | --- |
|  | Data points (n) | Popula-tion (n) | Propor-tion (%) | 95% CI | Data points (n) | Popula-tion (n) | Propor-tion (%) | 95% CI |
| Eluvia™ | 9 | 1427 | 7.27 | 5.23; 10.02 | 5 | 507 | 13.45 | 9.06; 19.51 |
| Zilver® PTX® | 22 | 4454 | 13.08 | 9.82; 17.23 | 10 | 2394 | 22.53 | 16.35; 30.19 |
| Viabahn | 6 | 619 | 18.9 | 15.18; 23.28 | 5 | 571 | 26.62 | 19.14; 35.72 |
| BMS | 46 | 8331 | 14.07 | 12.05; 16.37 | 30 | 4545 | 21.1 | 17.57; 25.12 |

* Only studies with a rating of fair, good, or excellent were included in this analysis. Abbreviations: CI, confidence interval.

Supplementary Table 9. Mortality at 12 and 24 months post stenting based on the use of core laboratory adjudication*

| Stent type | 12 months | | | | 24 months | | | |
| --- | --- | --- | --- | --- | --- | --- | --- | --- |
|  | Data points (n) | Population (n) | Proportion (%) | 95% CI | Data points (n) | Population (n) | Proportion (%) | 95% CI |
| Eluvia™ | 4 | 872 | 2.29 | 1.13; 4.60 |  |  |  |  |
| Zilver® PTX® | 6 | 1540 | 3.54 | 1.66; 7.41 | 2 | 873 | 4.81 | 0.67; 27.38 |
| Viabahn | 2 | 145 | 5.52 | 0.06; 85.58 |  |  |  |  |
| BMS | 15 | 2725 | 2.77 | 1.86; 4. 12 | 7 | 1201 | 5.89 | 2.74; 12.19 |

*Only studies that reported using core laboratory adjudication were included in this analysis. Abbreviations: CI, confidence interval.

Supplementary Table 10. Mortality at 12 and 24 months post stenting based on the Downs and Black Quality Appraisal score*

| Stent type | 12 months | | | | 24 months | | | |
| --- | --- | --- | --- | --- | --- | --- | --- | --- |
|  | Data points (n) | Popula-tion (n) | Propor-tion (%) | 95% CI | Data points (n) | Popula-tion (n) | Propor-tion (%) | 95% CI |
| Eluvia™ | 4 | 894 | 2.35 | 1.18;4.63 | 2 | 145 | 10.42 | 0.02;98.37 |
| Zilver® PTX® | 15 | 2685 | 4.63 | 3.04;7 | 11 | 2754 | 9.26 | 6.55;12.95 |
| Viabahn | 4 | 376 | 9.31 | 5.51;15.29 | 2 | 207 | 16.91 | 1.89;68.22 |
| BMS | 42 | 7956 | 3.63 | 2.65;4.95 | 20 | 6337 | 6.9 | 4.16;11.23 |

* Only studies with a rating of fair, good, or excellent were included in this analysis. Abbreviations: CI, confidence interval.

Supplementary Table 11. Major amputations at 12 and 24 months post stenting

| Stent type | 12 months | | | | 24 months | | | |
| --- | --- | --- | --- | --- | --- | --- | --- | --- |
|  | Data points (n) | Popula-tion (n) | Propor-tion(%) | 95% CI | Data points (n) | Popula-tion (n) | Propor-tion (%) | 95% CI |
| Eluvia™ | 9 | 1323 | 0.45 | 0.18; 1.16 | 5 | 675 | 1.33 | 0.21; 7.96 |
| Zilver® PTX® | 10 | 1939 | 0.73 | 0.18; 2.93 | 8 | 1489 | 1.21 | 0.69; 2.1 |
| Viabahn | 3 | 489 | 1.42 | 0.07; 23.68 | 4 | 563 | 2.31 | 0.96; 5.46 |
| BMS | 27 | 4255 | 0.97 | 0.47; 1.99 | 15 | 2716 | 2.06 | 0.84; 4.97 |

Abbreviations: CI, confidence interval.

Supplementary Table 12. Stent fractures at 12 and 24 months post stenting

| Stent type | 12 months | | | | 24 months | | | |
| --- | --- | --- | --- | --- | --- | --- | --- | --- |
|  | Data points (n) | Popula-tion (n) | Propor-tion (%) | 95% CI | Data points (n) | Popula-tion (n) | Propor-tion (%) | 95% CI |
| Eluvia™ | 3 | 413 | 0.18 | 0; 79.28 | 1 | 57 | 0 | 0; 6.27 |
| Zilver® PTX® | 5 | 1419 | 4.78 | 0.30; 45.29 | 2 | 110 | 0.91 | 0; 99.97 |
| Viabahn | 2 | 87 | 1.15 | 0; 99.98 | 1 | 45 | 4.44 | 0.54; 15.15 |
| BMS | 29 | 4574 | 1.14 | 0.43; 2.99 | 16 | 1995 | 0.40 | 0.03; 4.62 |

*Because different papers reported the 12- and 24-month outcomes, the proportion of stent fractures is lower at 24 months versus 12 months. Abbreviations: CI, confidence interval.

Supplementary Table 13. Clinical improvement at 12 months post stenting*

| Stent type | 12 months | | | |
| --- | --- | --- | --- | --- |
|  | Data points (n) | Population (n) | Proportion (%) | 95% CI |
| Eluvia™ | 3 | 767 | 86.49 | 74.81; 93.24 |
| Zilver® PTX® | 6 | 1267 | 80.65 | 71.62; 87.31 |
| Viabahn | 1 | 89 | 91.01 | 83.05; 96.04 |
| BMS | 19 | 2021 | 79.7 | 75.24; 83.54 |

* It was not possible to repeat the analyses for the 24-month timepoint due to the low number of studies and the heterogeneity in reporting this outcome. Abbreviations: CI, confidence interval.

Supplementary figures


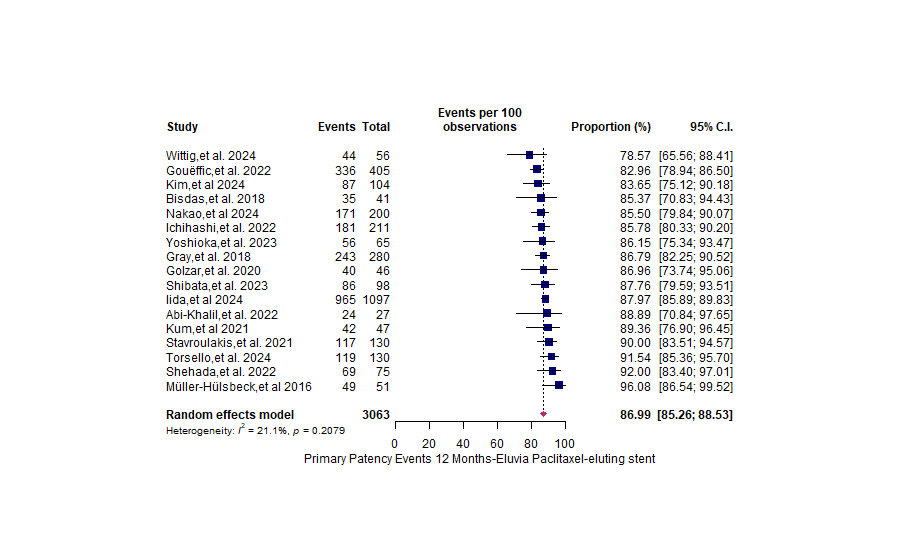


Supplementary Figure 1. Primary patency at 12 months for Eluvia™.


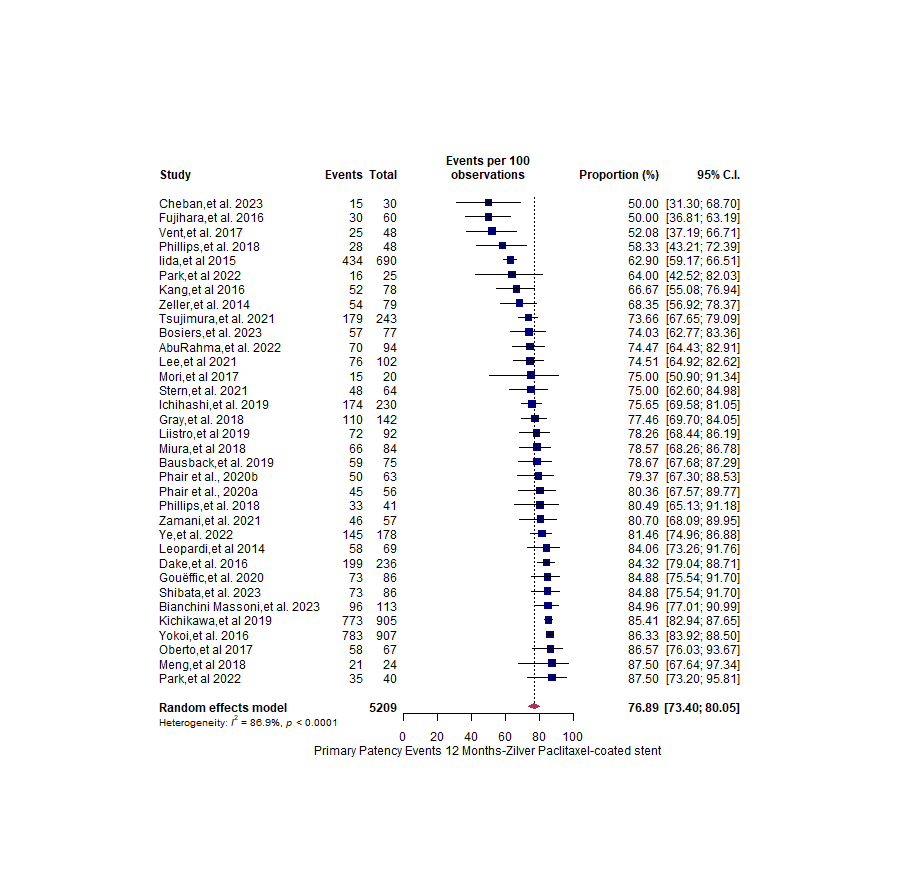


Supplementary Figure 2. Primary patency at 12 months for Zilver® PTX®.


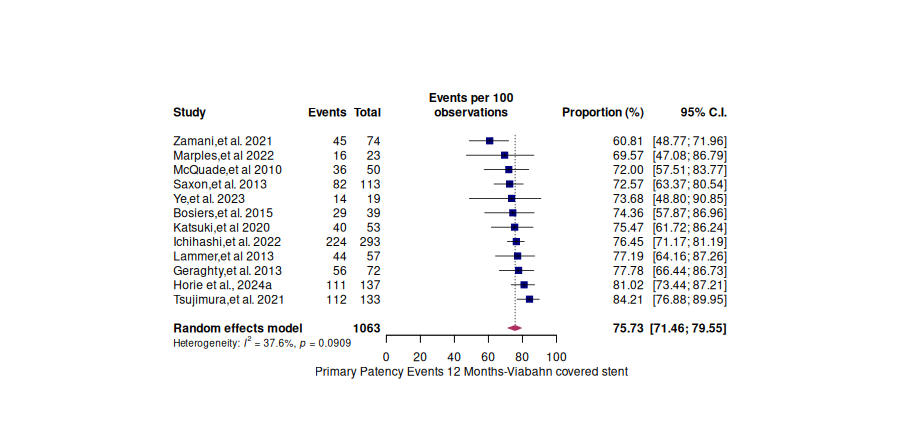


Supplementary Figure 3. Primary patency at 12 months for Viabahn.


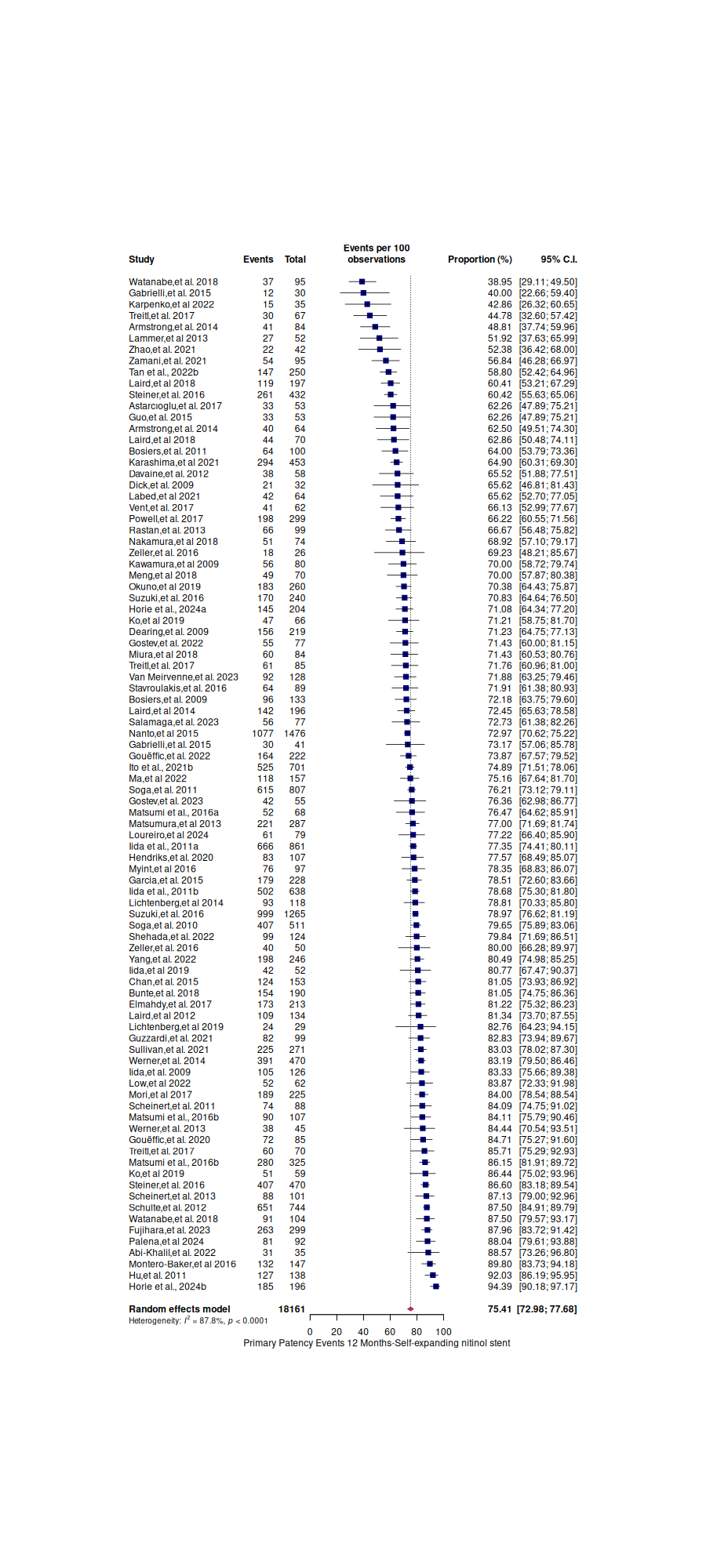


Supplementary Figure 4. Primary patency at 12 months for BMS.


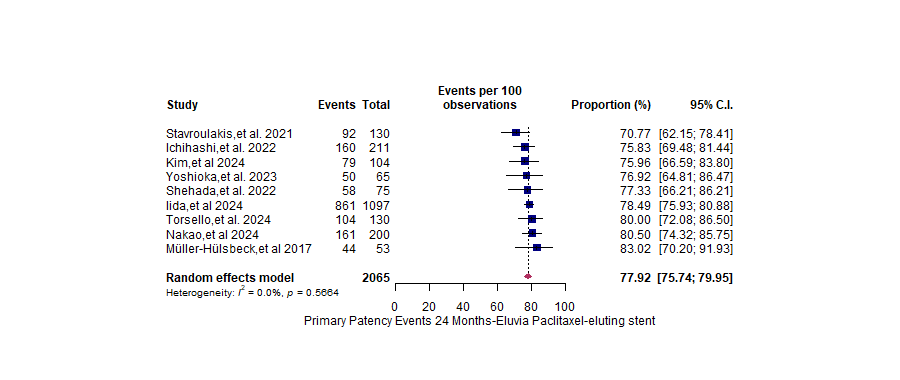


Supplementary Figure 5. Primary patency at 24 months for Eluvia™.


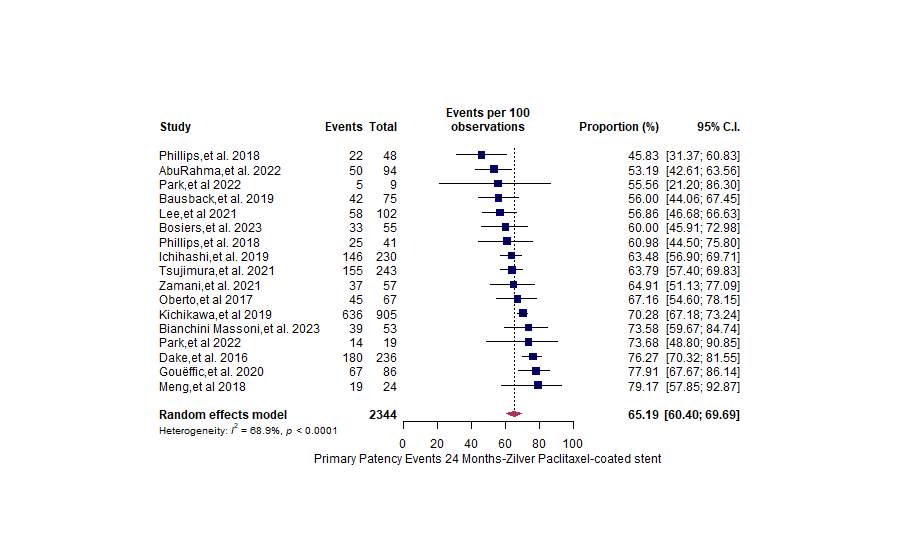


Supplementary Figure 6. Primary patency at 24 months for Zilver® PTX®.


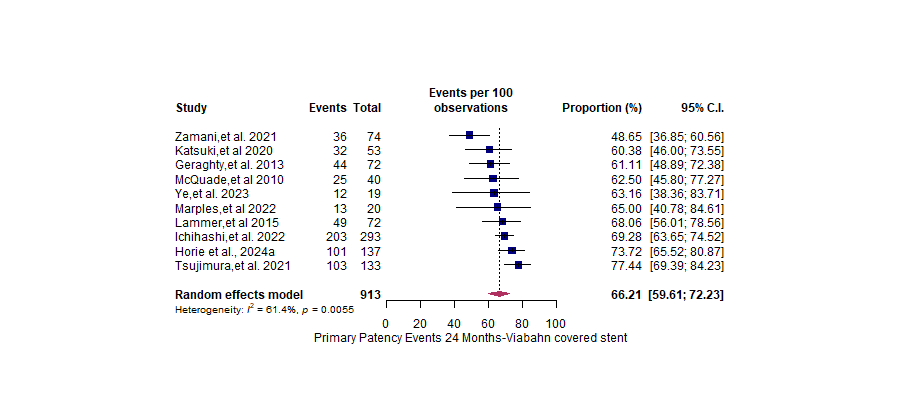


Supplementary Figure 7. Primary patency at 24 months for Viabahn.


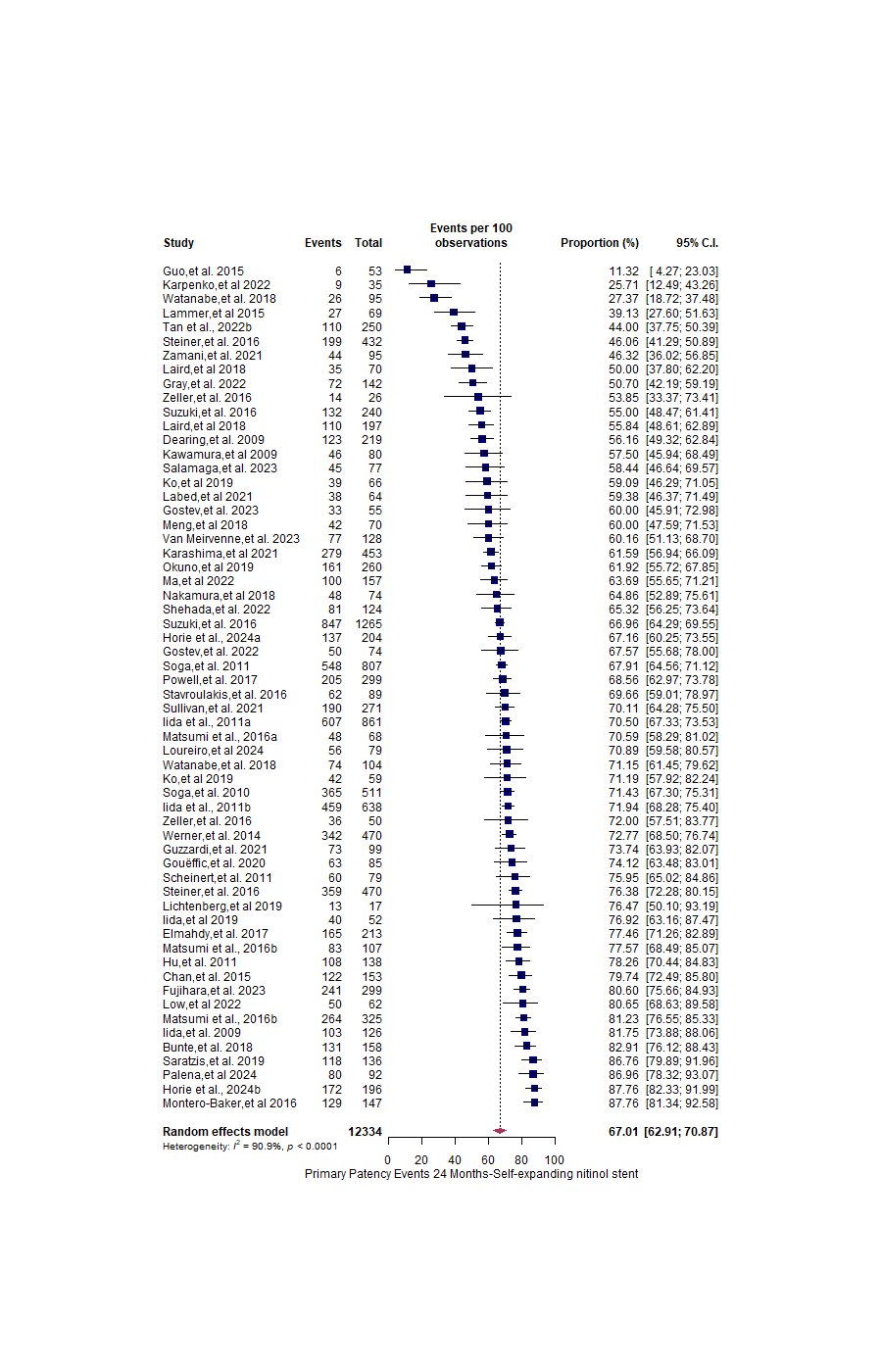


Supplementary Figure 8. Primary patency at 24 months for BMS.


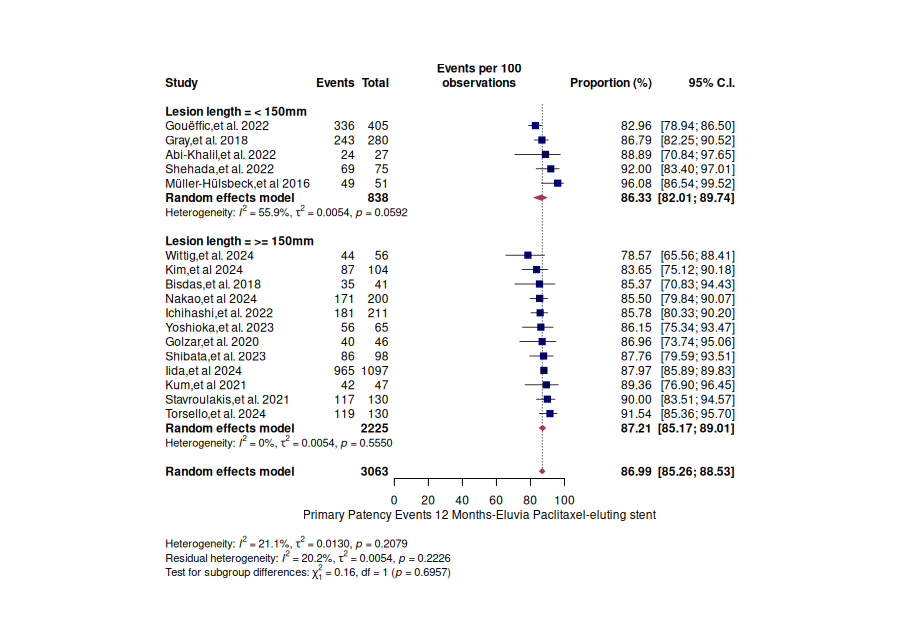


Supplementary Figure 9. Primary patency in short and long lesions at 12 months for Eluvia™.


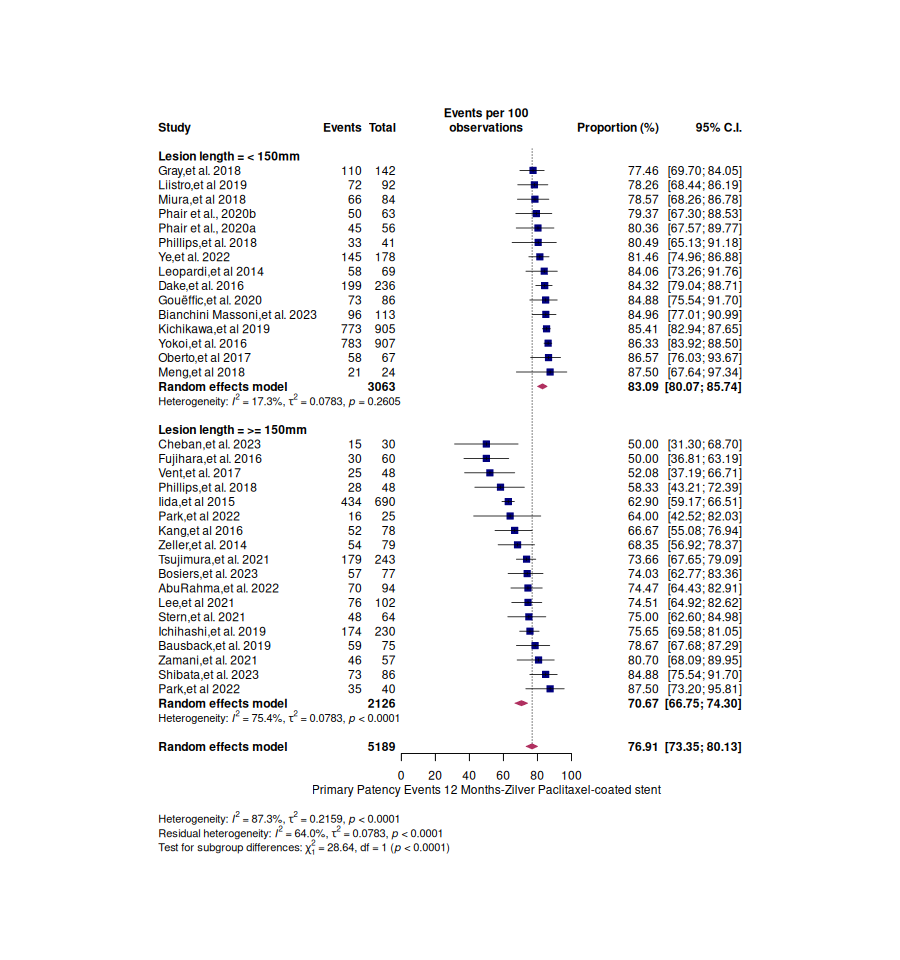


Supplementary Figure 10. Primary patency in short and long lesions at 12 months for Zilver® PTX®.


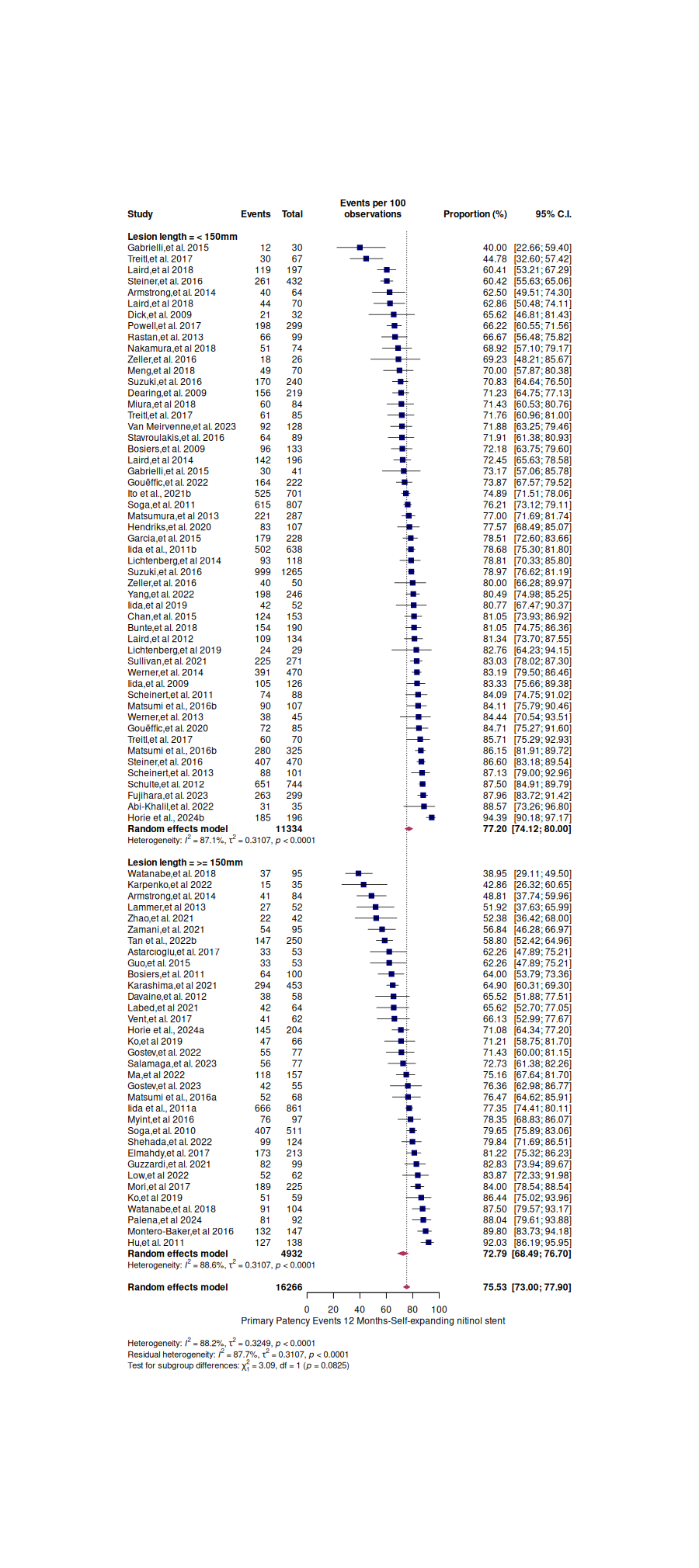


Supplementary Figure 11. Primary patency in short and long lesions at 12 months for BMS.


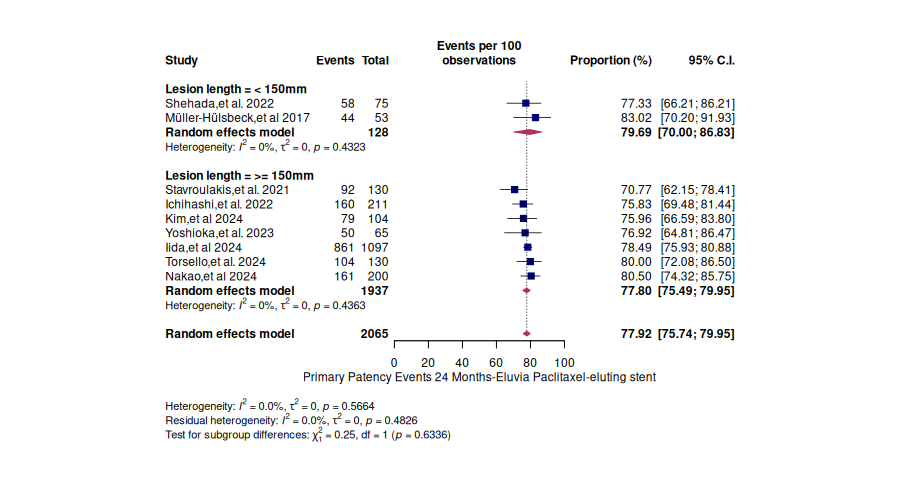


Supplementary Figure 12. Primary patency in short and long lesions at 24 months for Eluvia™.


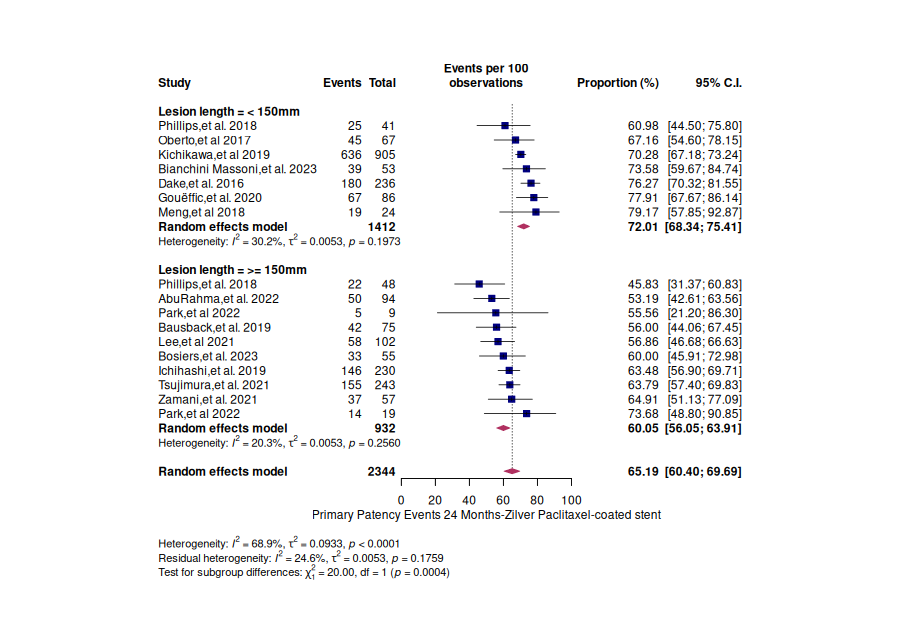


Supplementary Figure 13. Primary patency in short and long lesions at 24 months for Zilver® PTX®.


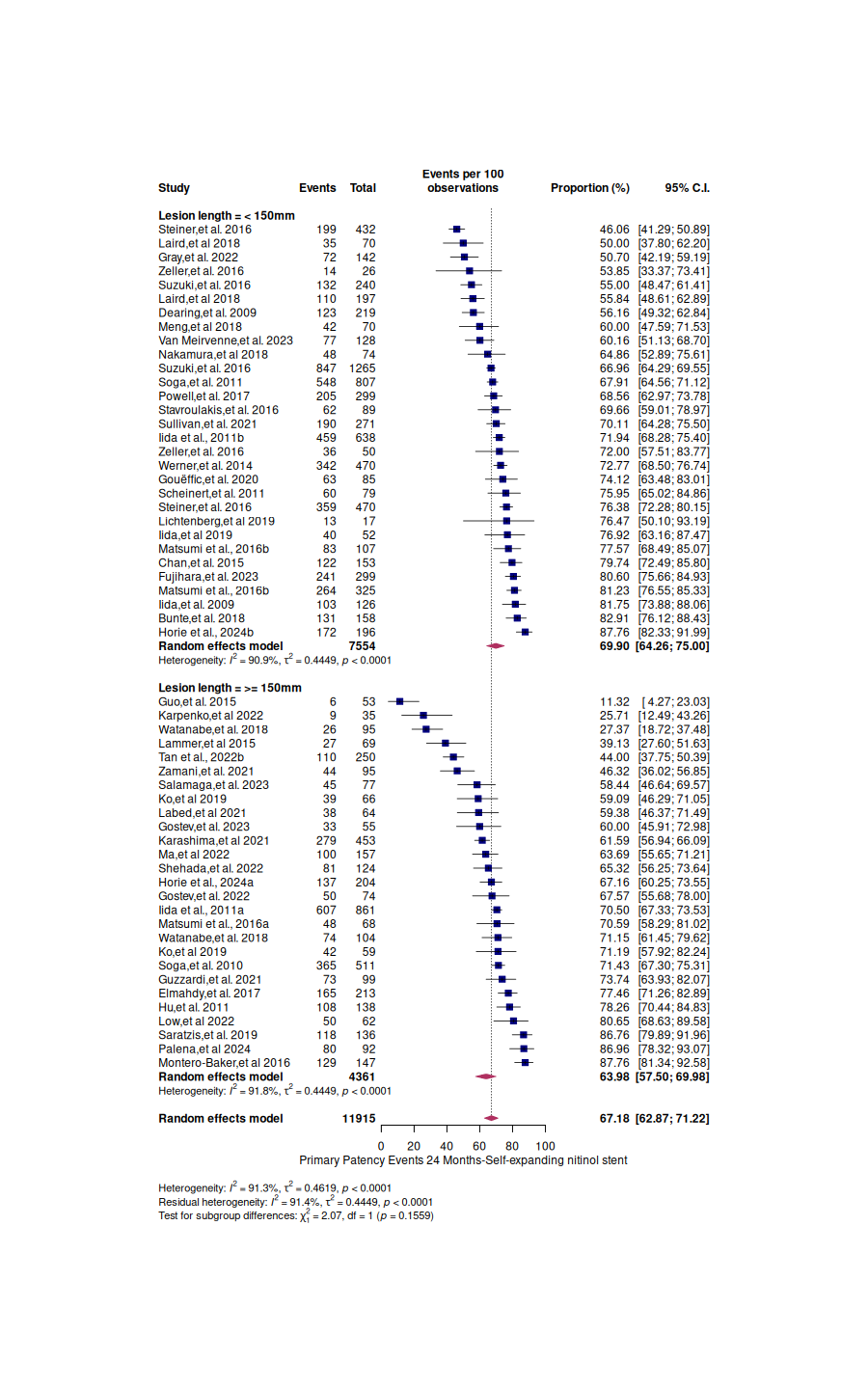


Supplementary Figure 14. Primary patency in short and long lesions at 24 months for BMS.


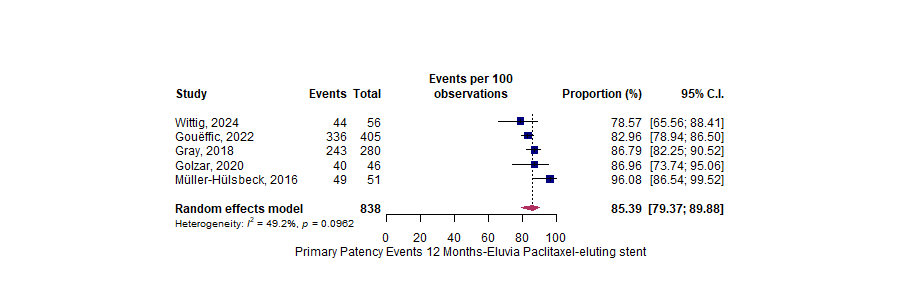


Supplementary Figure 15. Primary patency at 12 months for Eluvia™ according to studies that reported using core laboratory adjudication.


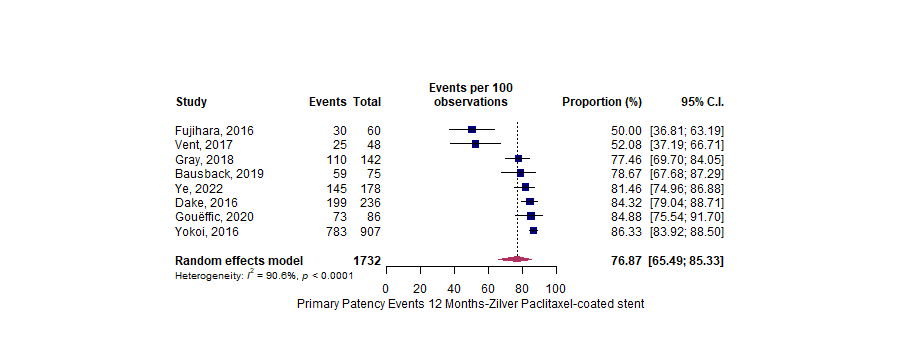


Supplementary Figure 16. Primary patency at 12 months for Zilver® PTX® according to studies that reported using core laboratory adjudication.


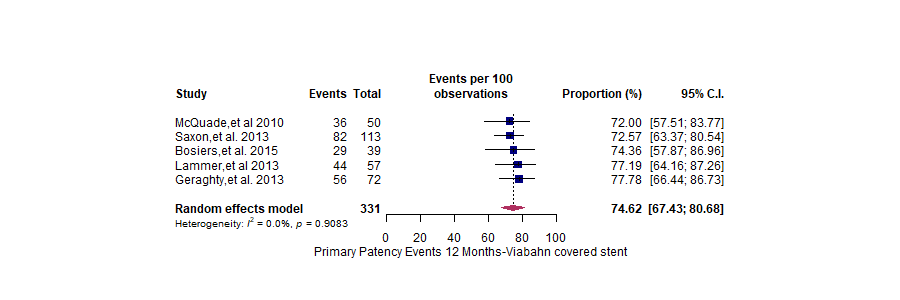


Supplementary Figure 17. Primary patency at 12 months for Viabahn according to studies that reported using core laboratory adjudication.


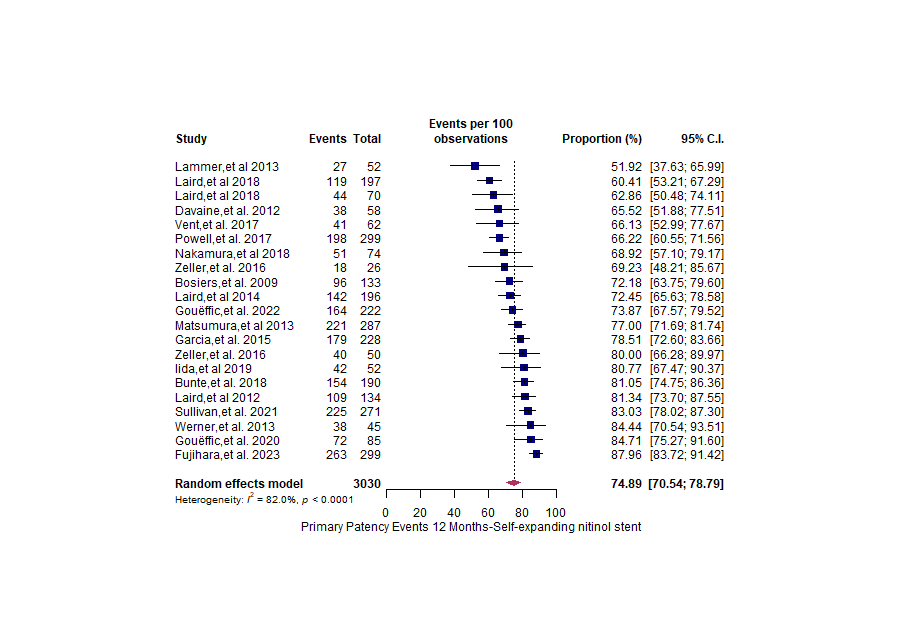


Supplementary Figure 18. Primary patency at 12 months for BMS according to studies that reported using core laboratory adjudication.


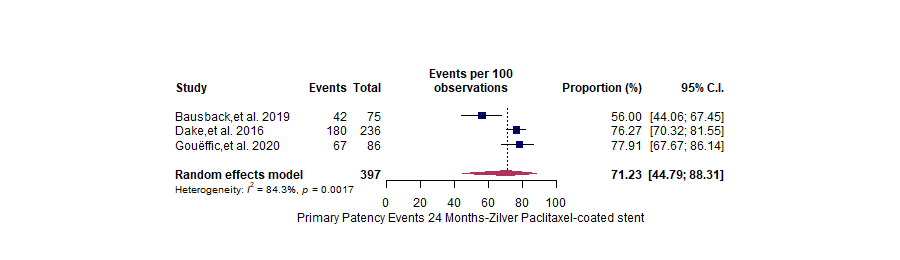


Supplementary Figure 19. Primary patency at 24 months for Zilver® PTX® according to studies that reported using core laboratory adjudication.


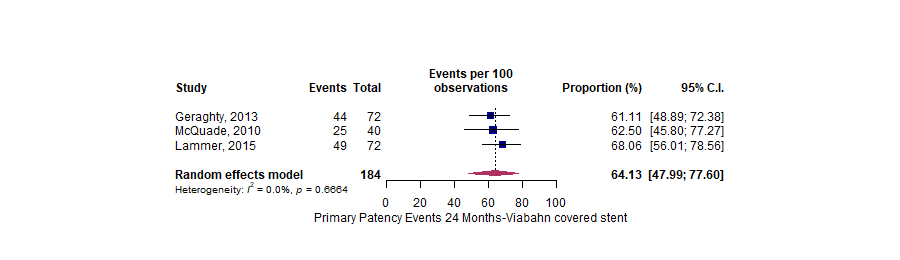


Supplementary Figure 20. Primary patency at 24 months for Viabahn according to studies that reported using core laboratory adjudication.


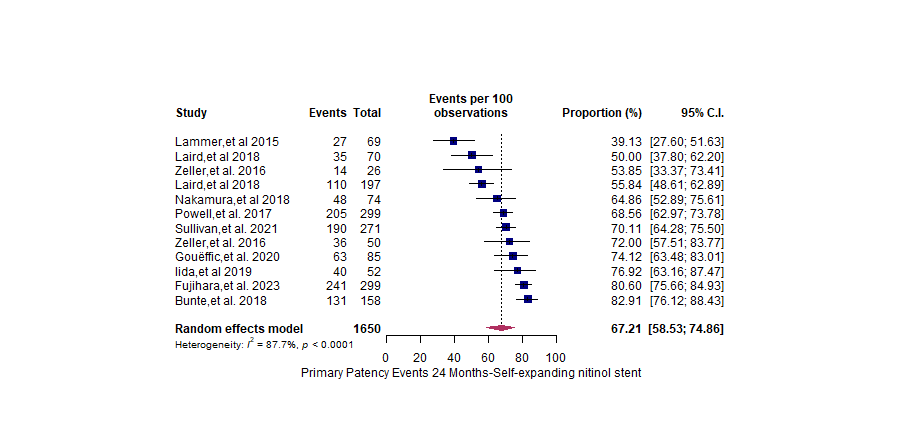


Supplementary Figure 21. Primary patency at 24 months for BMS according to studies that reported using core laboratory adjudication.


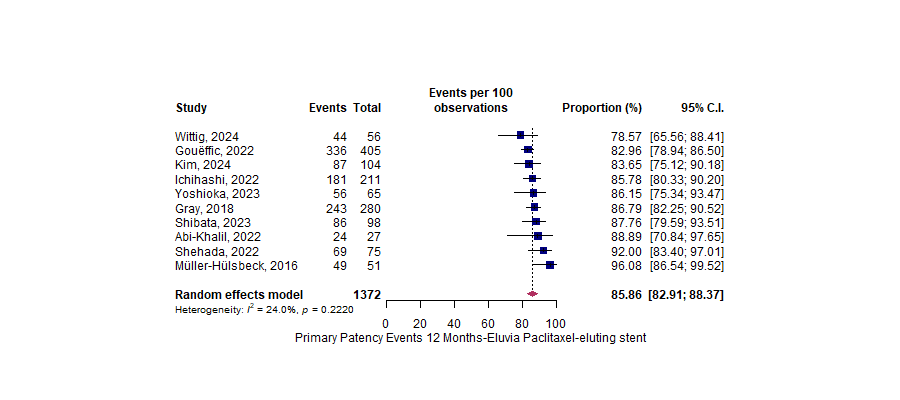


Supplementary Figure 22. Primary patency at 12 months for Eluvia™ according to studies with a Downs and Black Quality Appraisal rating of fair or above.


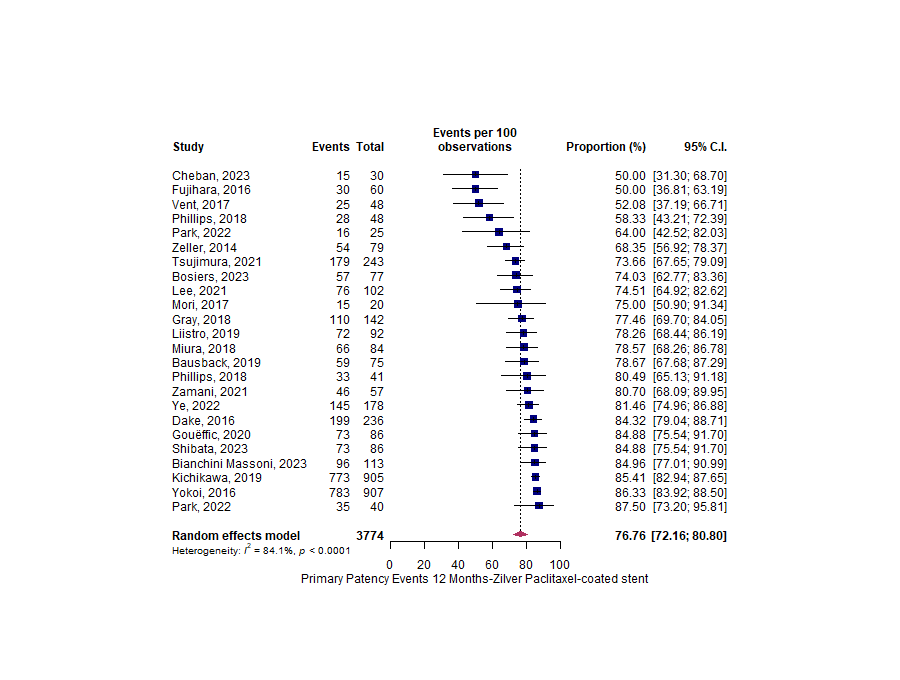


Supplementary Figure 23. Primary patency at 12 months for Zilver® PTX® according to studies with a Downs and Black Quality Appraisal rating of fair or above.


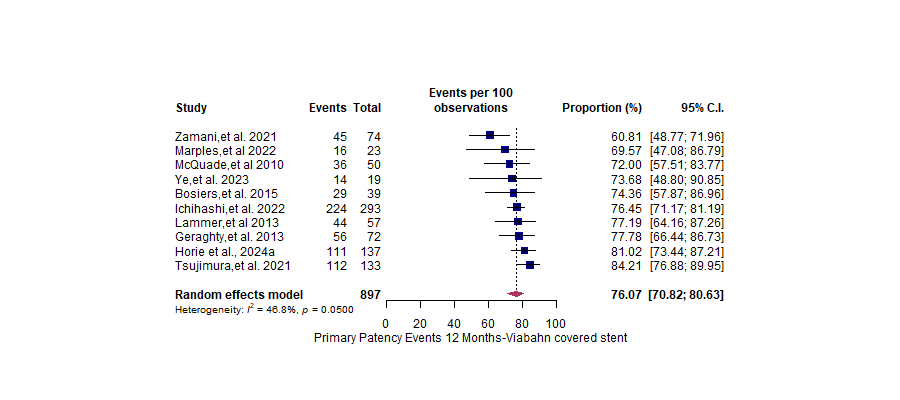


Supplementary Figure 24. Primary patency at 12 months for Viabahn according to studies with a Downs and Black Quality Appraisal rating of fair or above.


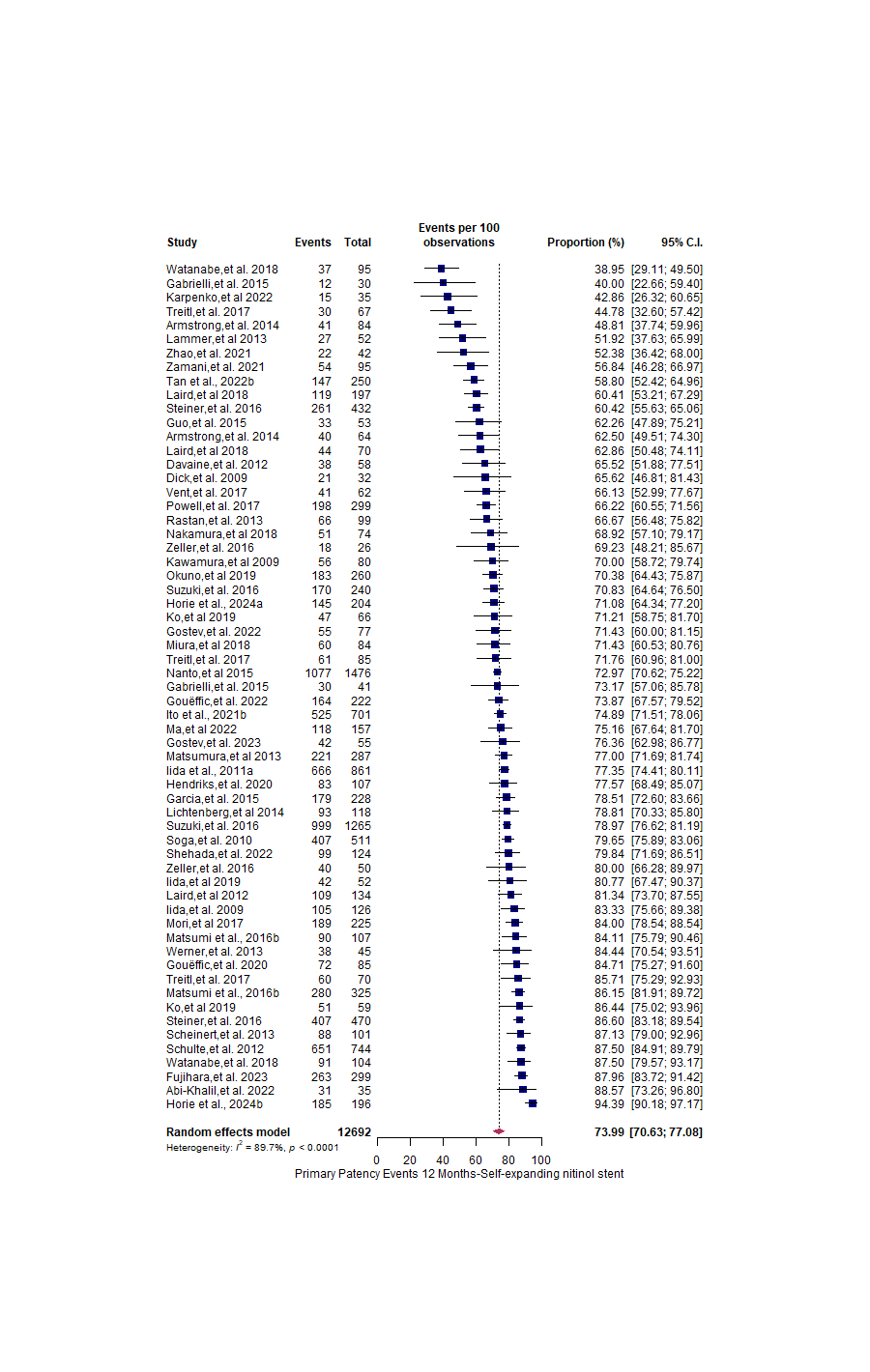


Supplementary Figure 25. Primary patency at 12 months for BMS according to studies with a Downs and Black Quality Appraisal rating of fair or above.


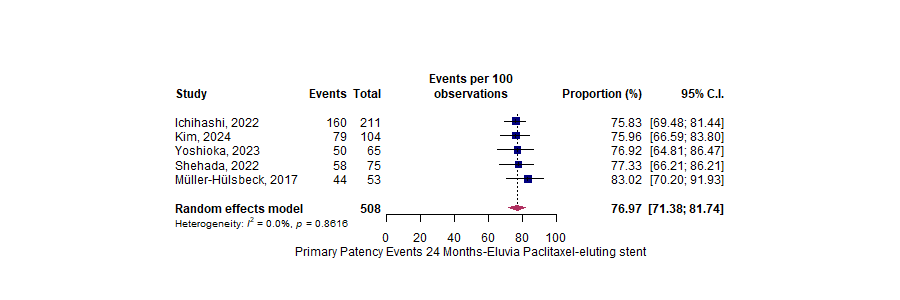
Supplementary Figure 26. Primary patency at 24 months for Eluvia™ according to studies with a Downs and Black Quality Appraisal rating of fair or above.


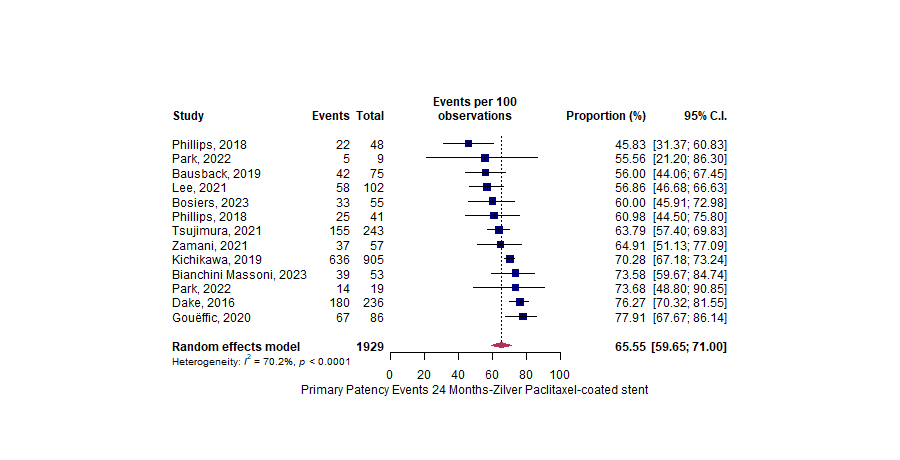


Supplementary Figure 27. Primary patency at 24 months for Zilver® PTX® according to studies with a Downs and Black Quality Appraisal rating of fair or above.


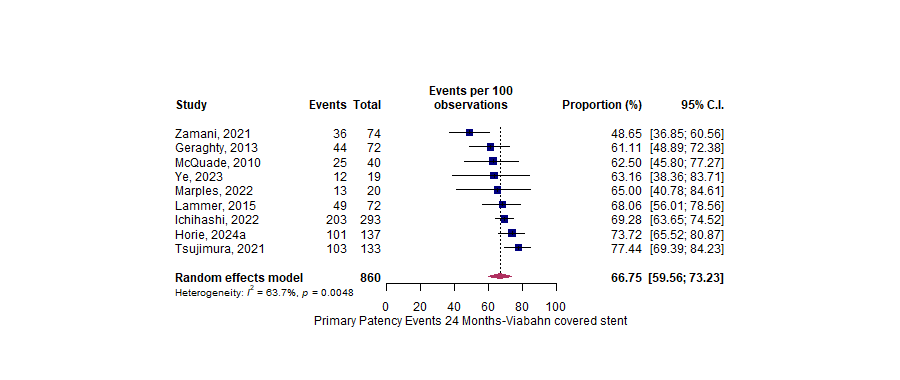


Supplementary Figure 28. Primary patency at 24 months for Viabahn according to studies with a Downs and Black Quality Appraisal rating of fair or above.


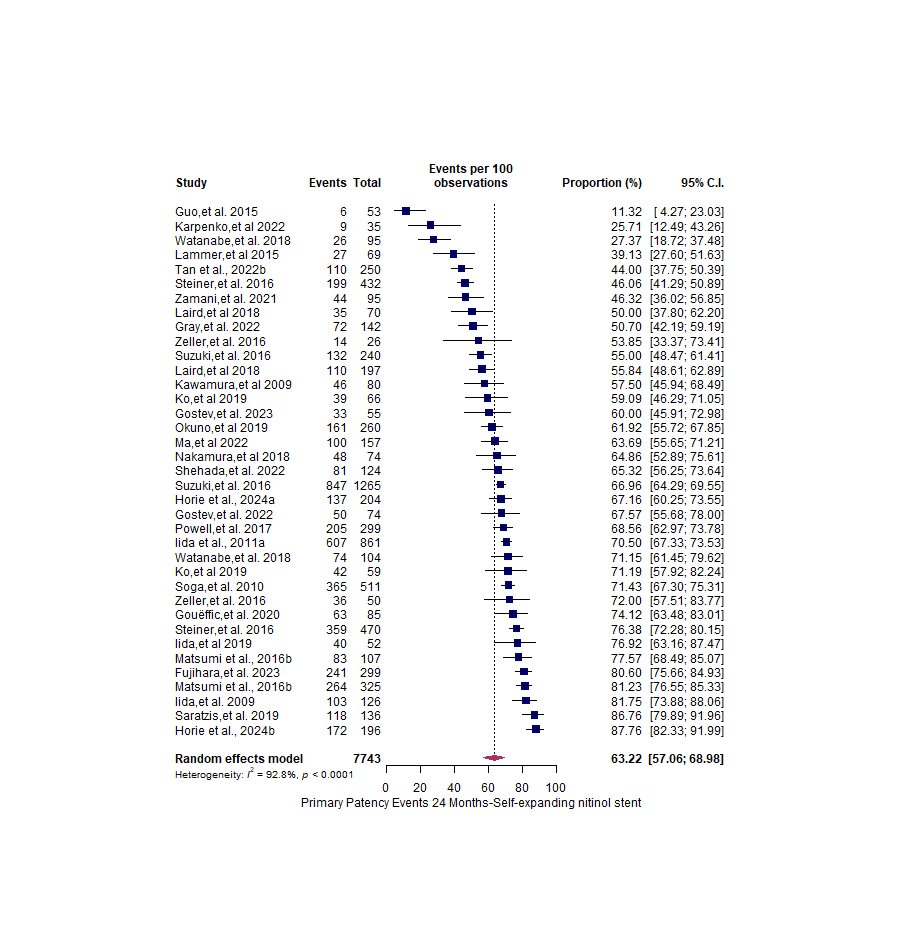


Supplementary Figure 29. Primary patency at 24 months for BMS according to studies with a Downs and Black Quality Appraisal rating of fair or above.


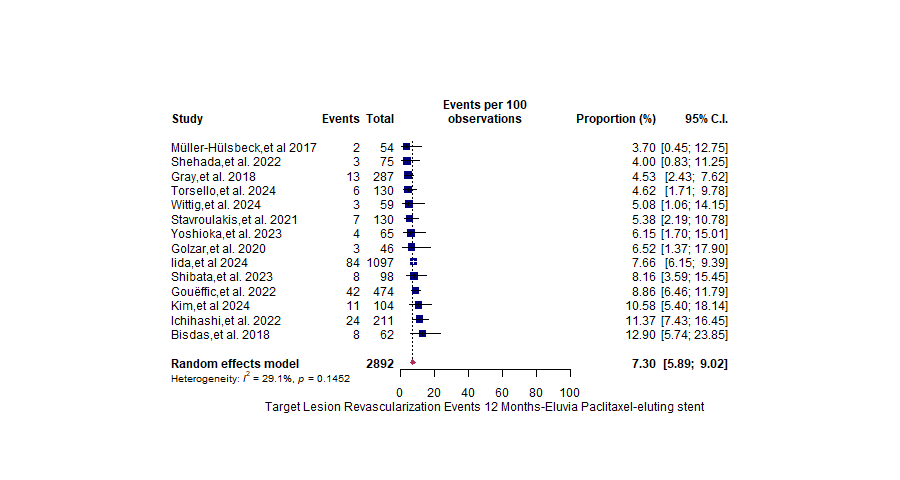


Supplementary Figure 30. Target lesion revascularisation at 12 months for Eluvia™.


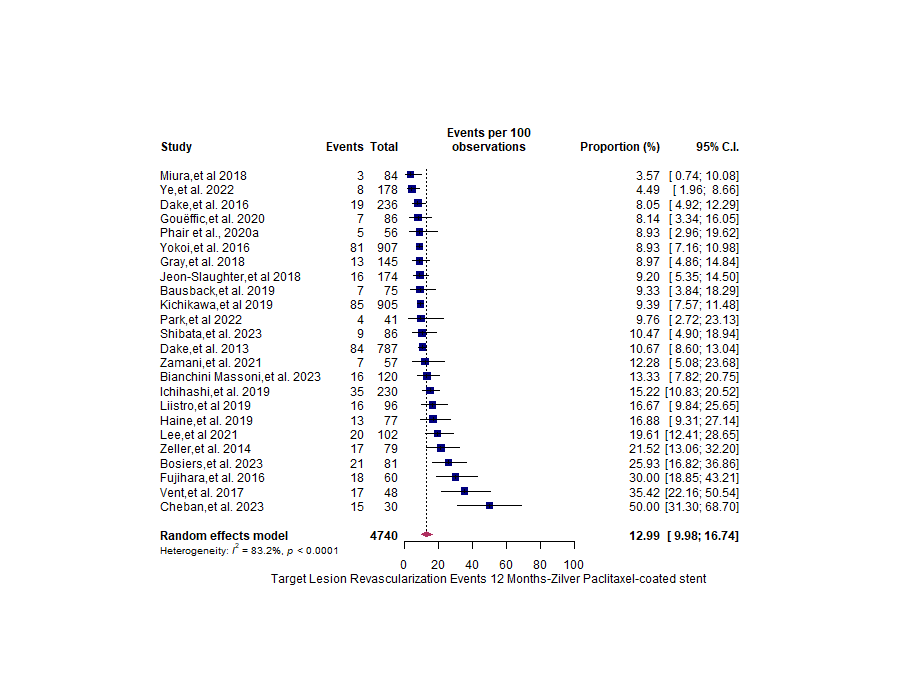


Supplementary Figure 31. Target lesion revascularisation at 12 months for Zilver® PTX®.


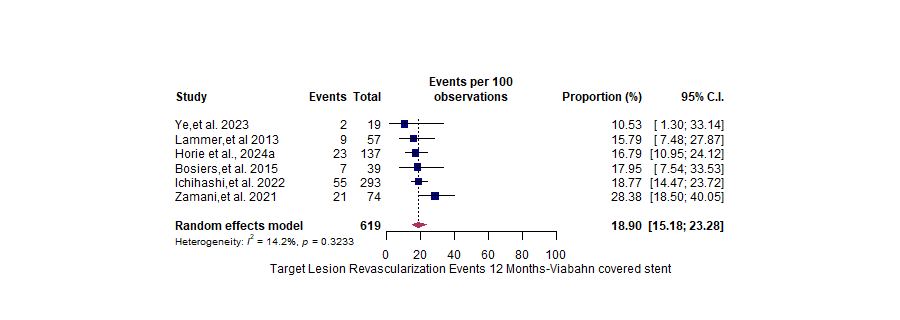


Supplementary Figure 32. Target lesion revascularisation at 12 months for Viabahn.


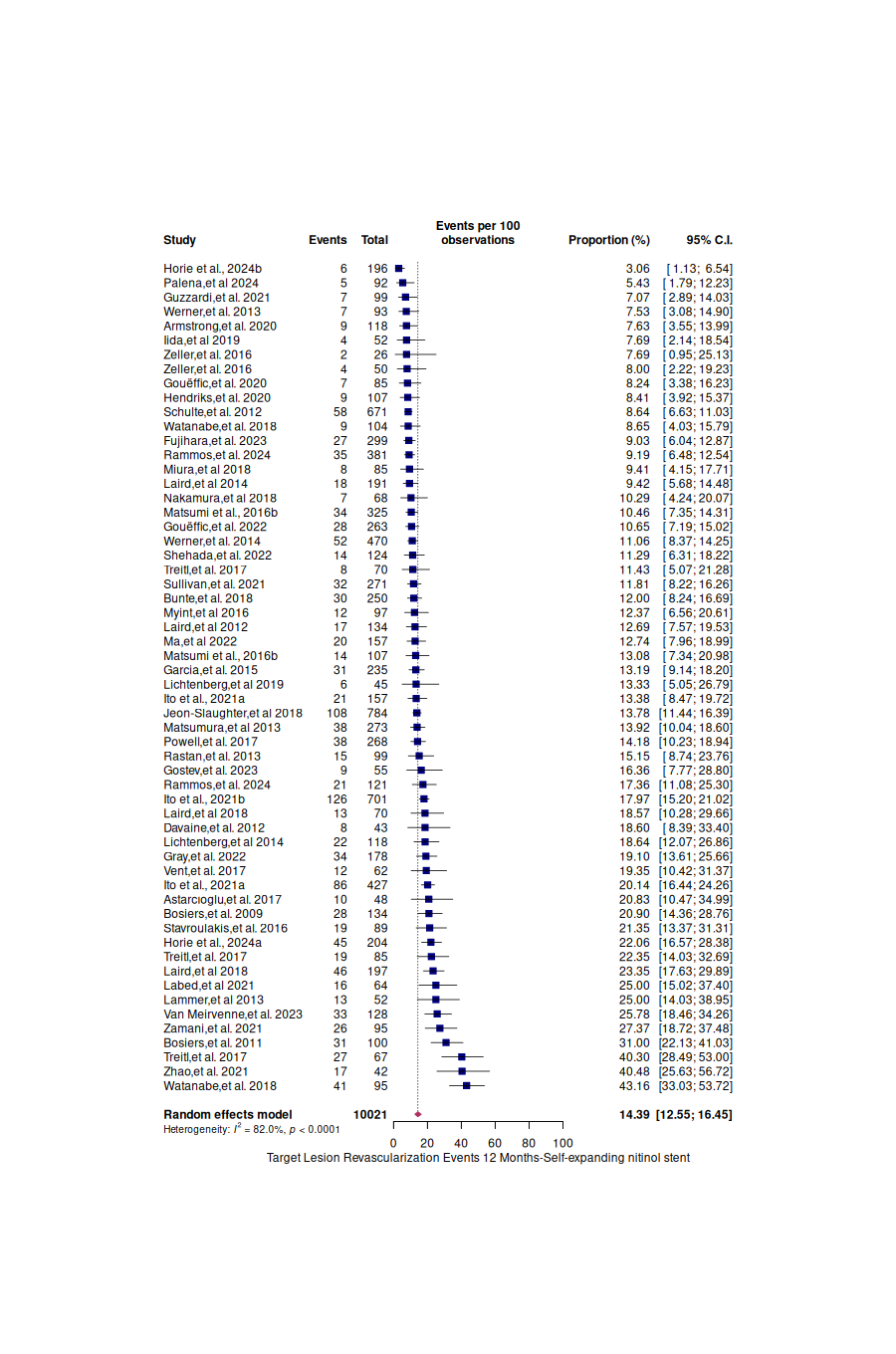


Supplementary Figure 33. Target lesion revascularisation at 12 months for BMS.


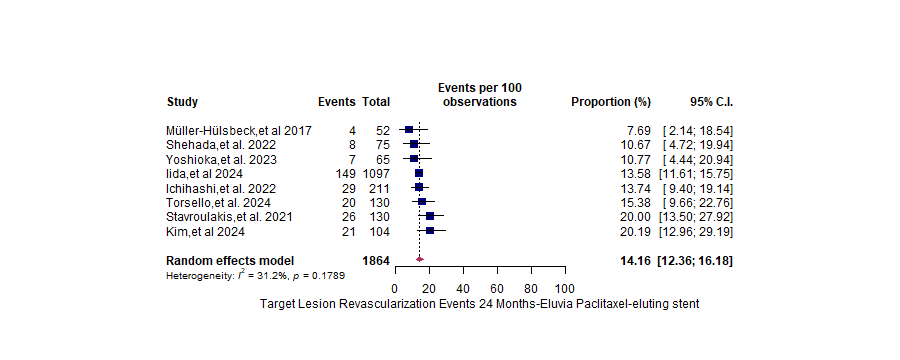


Supplementary Figure 34. Target lesion revascularisation at 24 months for Eluvia™.


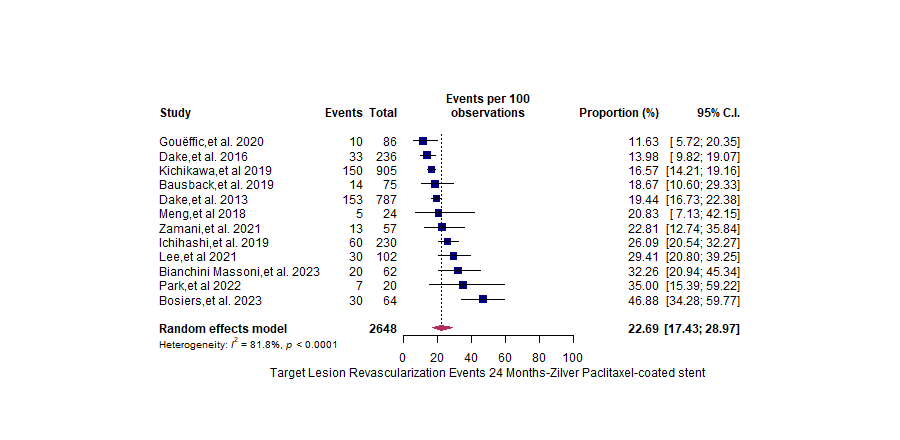


Supplementary Figure 35. Target lesion revascularisation at 24 months for Zilver® PTX®.


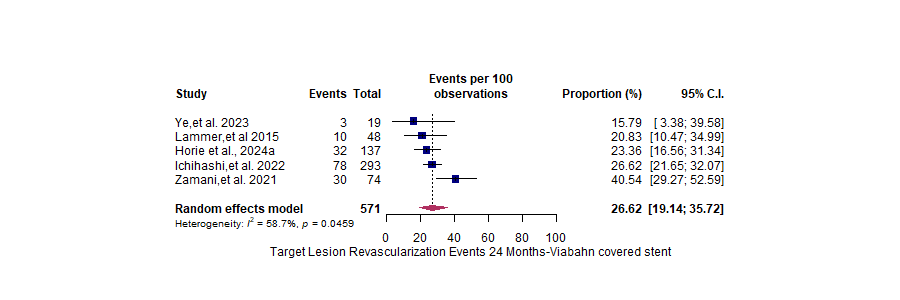


Supplementary Figure 36. Target lesion revascularisation at 24 months for Viabahn.


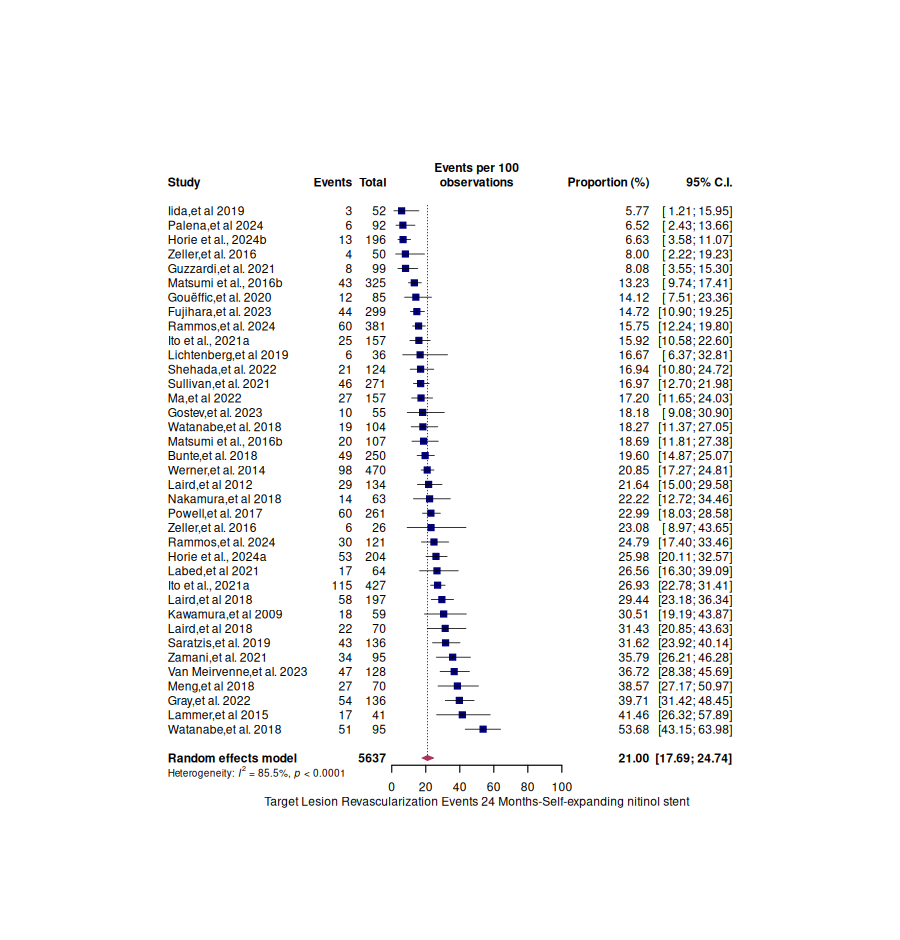


Supplementary Figure 37. Target lesion revascularisation at 24 months for BMS.


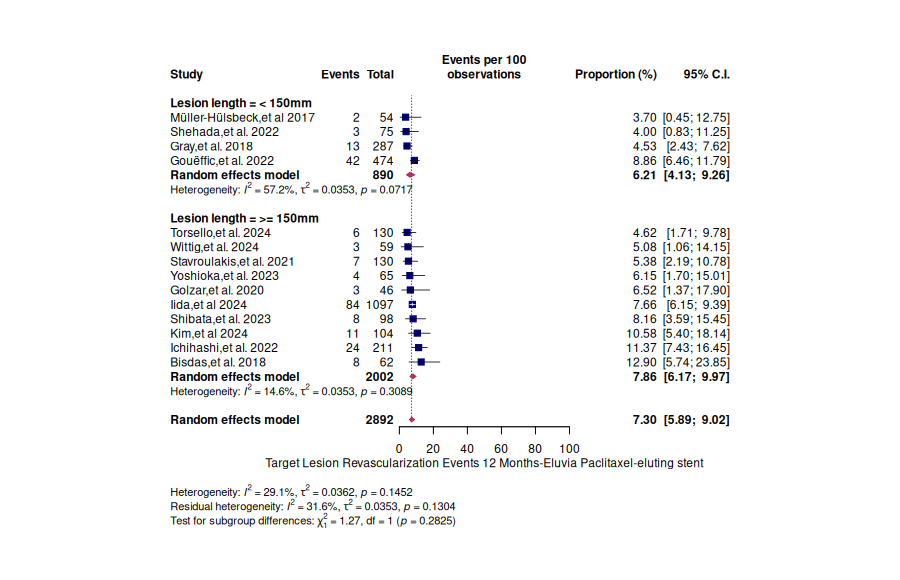


Supplementary Figure 38. Target lesion revascularisation in short and long lesions at 12 months for Eluvia™.


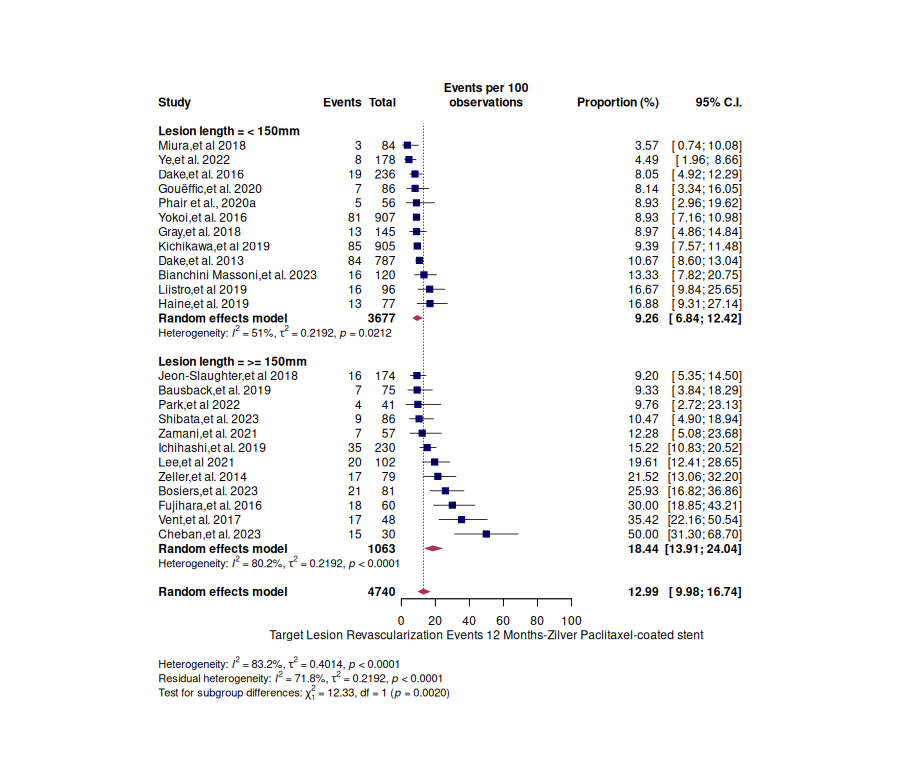


Supplementary Figure 39. Target lesion revascularisation in short and long lesions at 12 months for Zilver® PTX®.


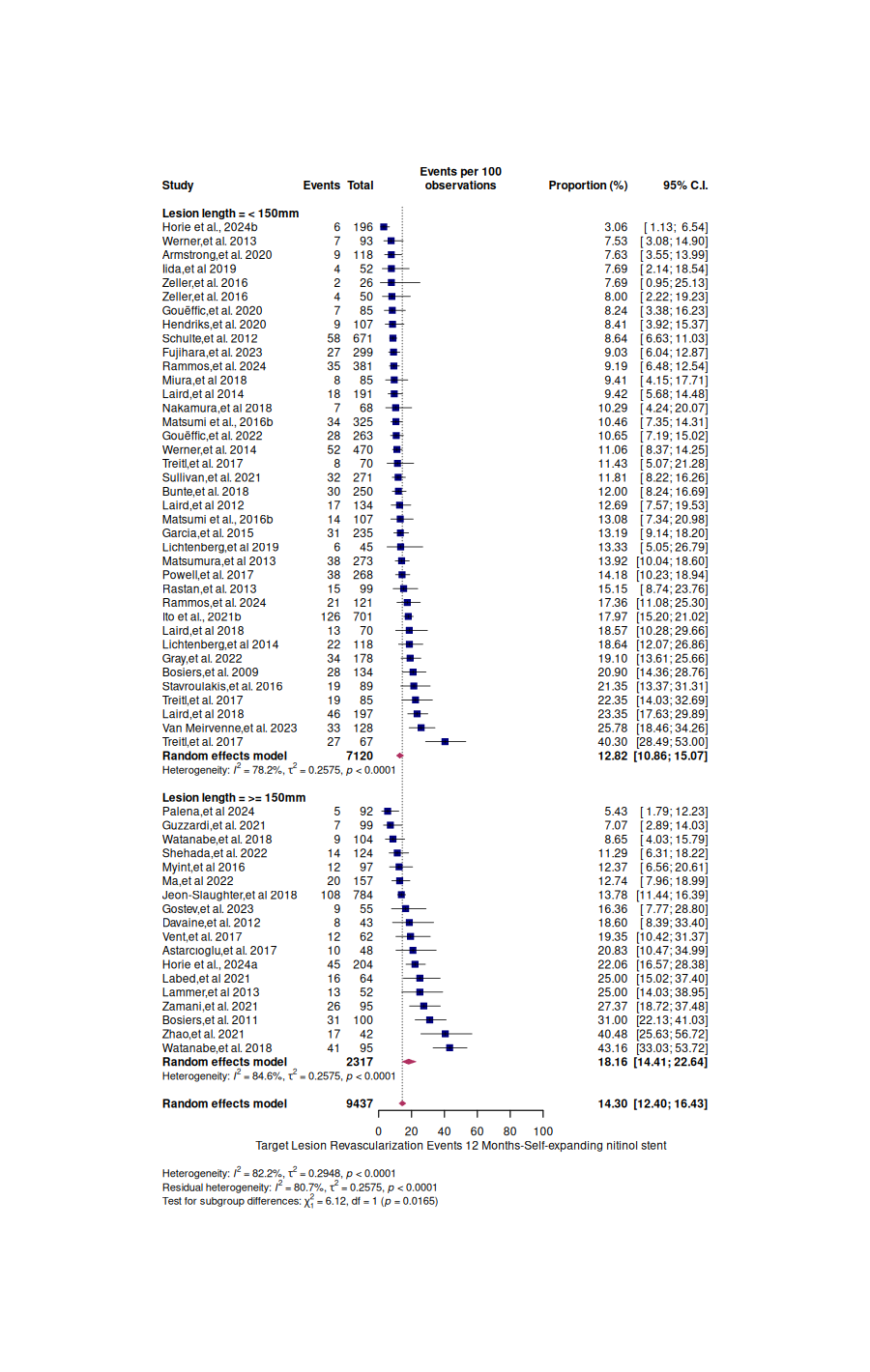


Supplementary Figure 40. Target lesion revascularisation in short and long lesions at 12 months for BMS.


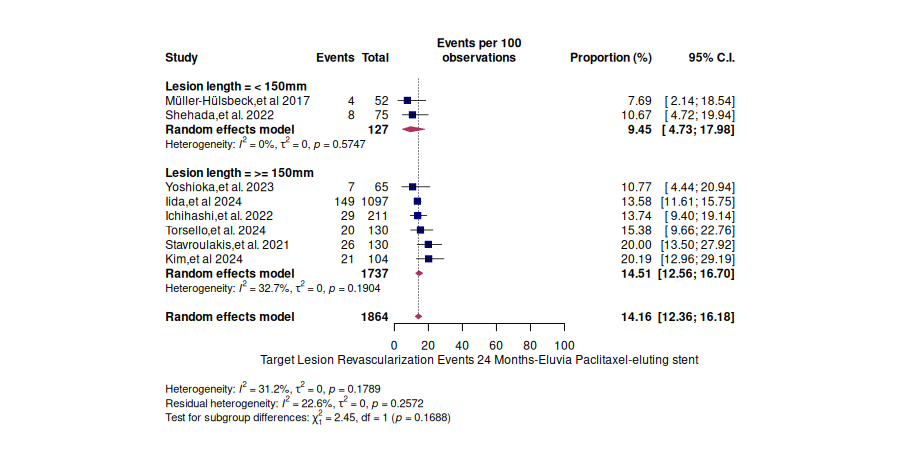


Supplementary Figure 41. Target lesion revascularisation in short and long lesions at 24 months for Eluvia™.


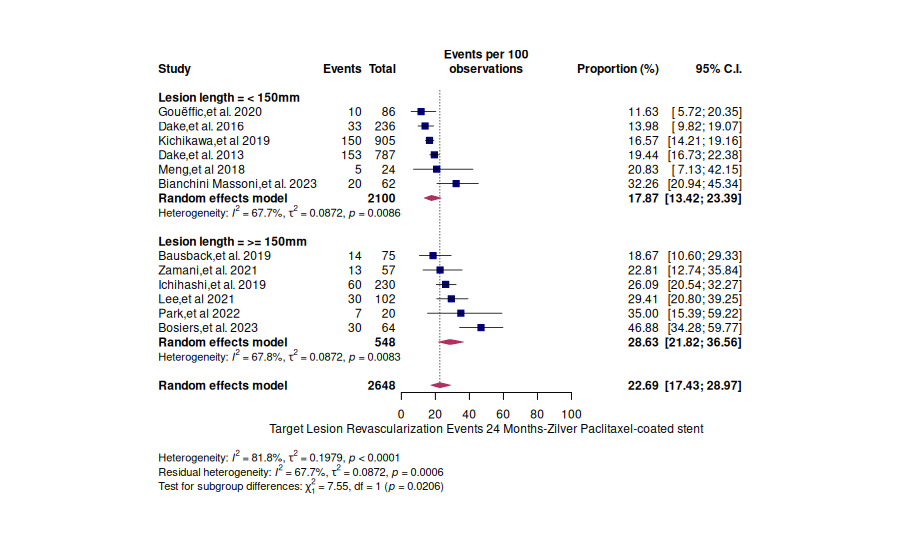


Supplementary Figure 42. Target lesion revascularisation in short and long lesions at 24 months for Zilver® PTX®.


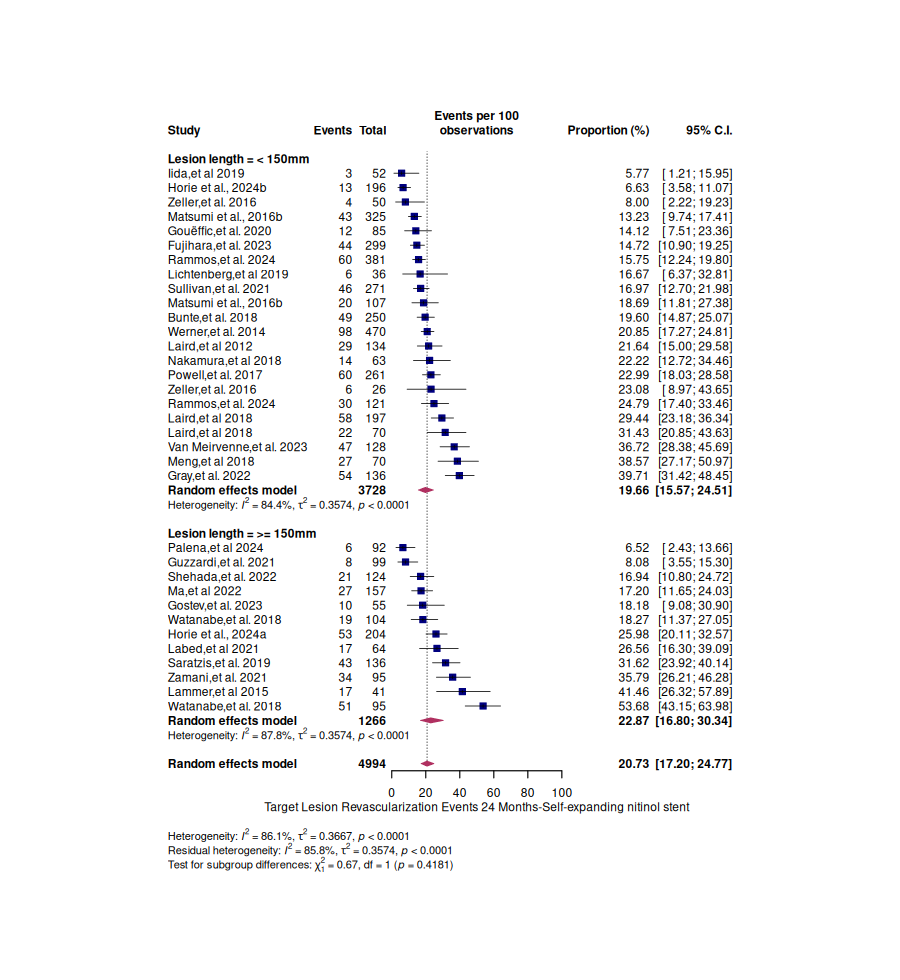


Supplementary Figure 43. Target lesion revascularisation in short and long lesions at 24 months for BMS.


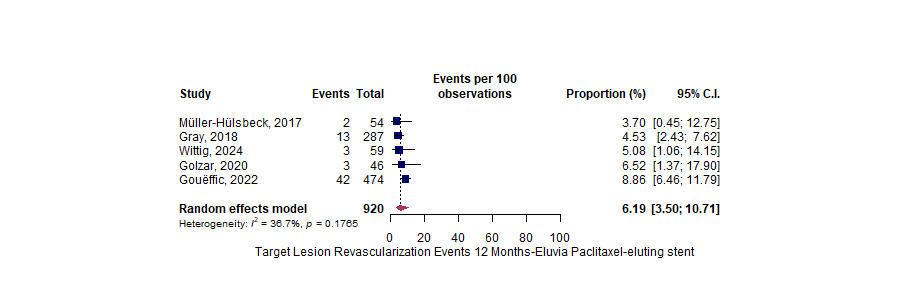


Supplementary Figure 44. Target lesion revascularisation at 12 months for Eluvia™ according to studies that reported using core laboratory adjudication.


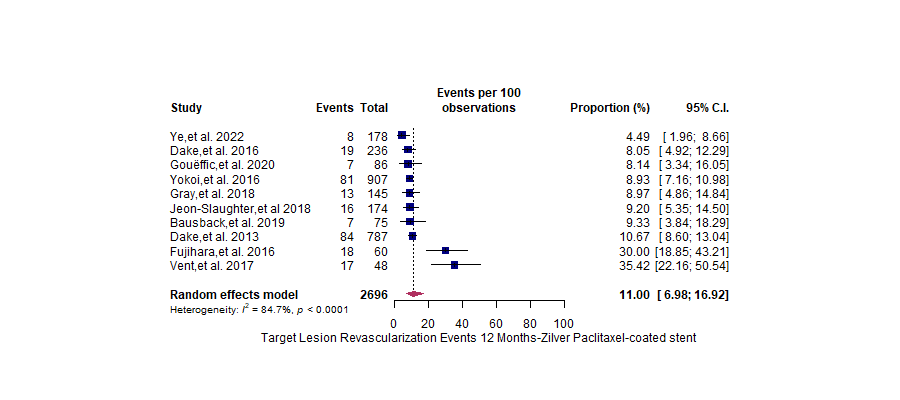


Supplementary Figure 45. Target lesion revascularisation at 12 months for Zilver® PTX® according to studies that reported using core laboratory adjudication.


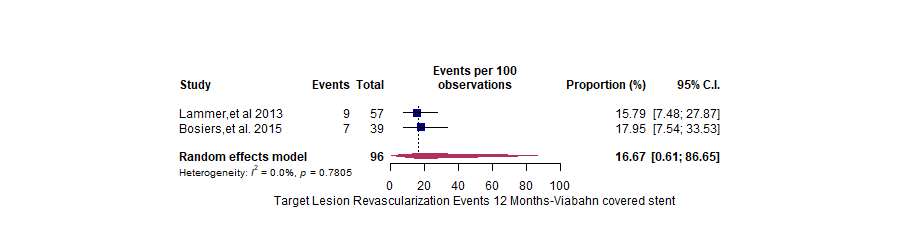


Supplementary Figure 46. Target lesion revascularisation at 12 months for Viabahn according to studies that reported using core laboratory adjudication.


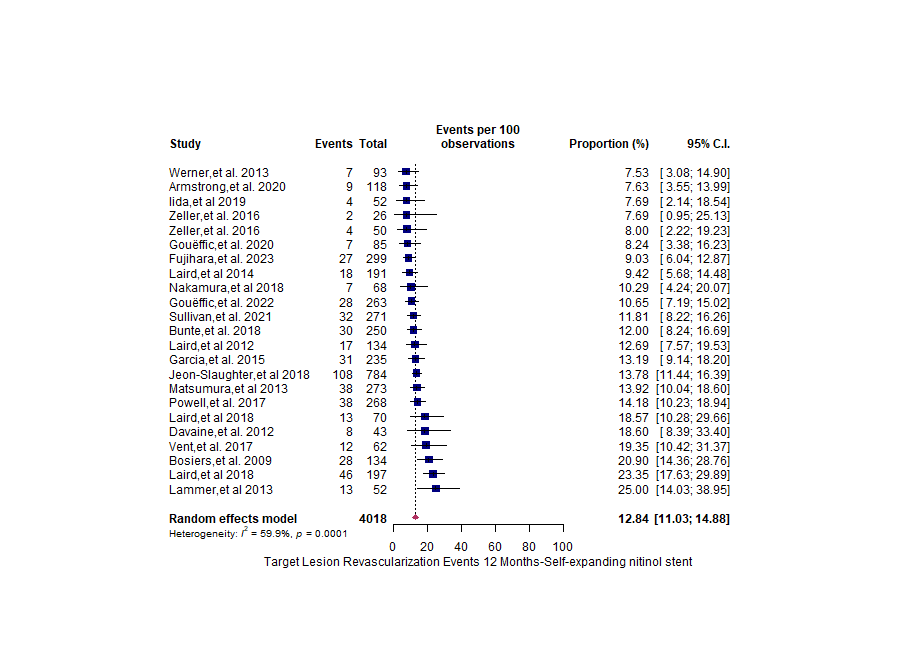


Supplementary Figure 47. Target lesion revascularisation at 12 months for BMS according to studies that reported using core laboratory adjudication.


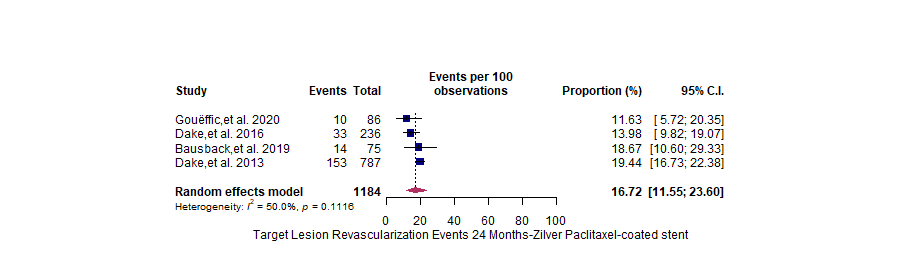


Supplementary Figure 48. Target lesion revascularisation at 24 months for Zilver® PTX® according to studies that reported using core laboratory adjudication.


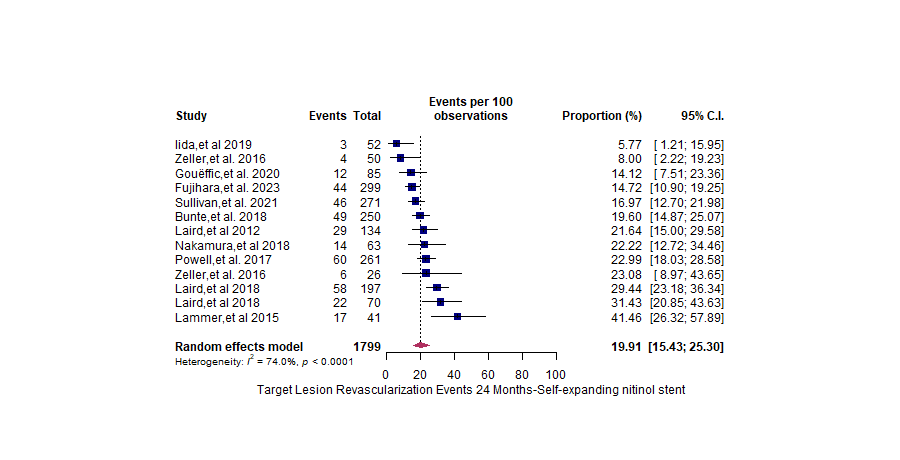


Supplementary Figure 49. Target lesion revascularisation at 24 months for BMS according to studies that reported using core laboratory adjudication.


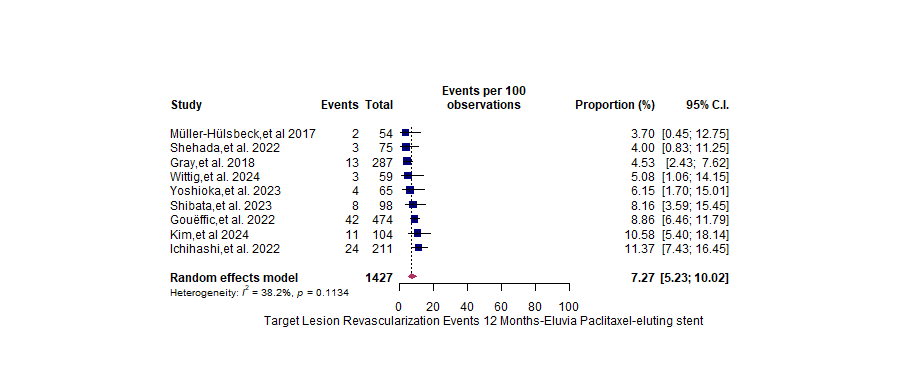


Supplementary Figure 50. Target lesion revascularisation at 12 months for Eluvia™ according to studies with a Downs and Black Quality Appraisal rating of fair or above.


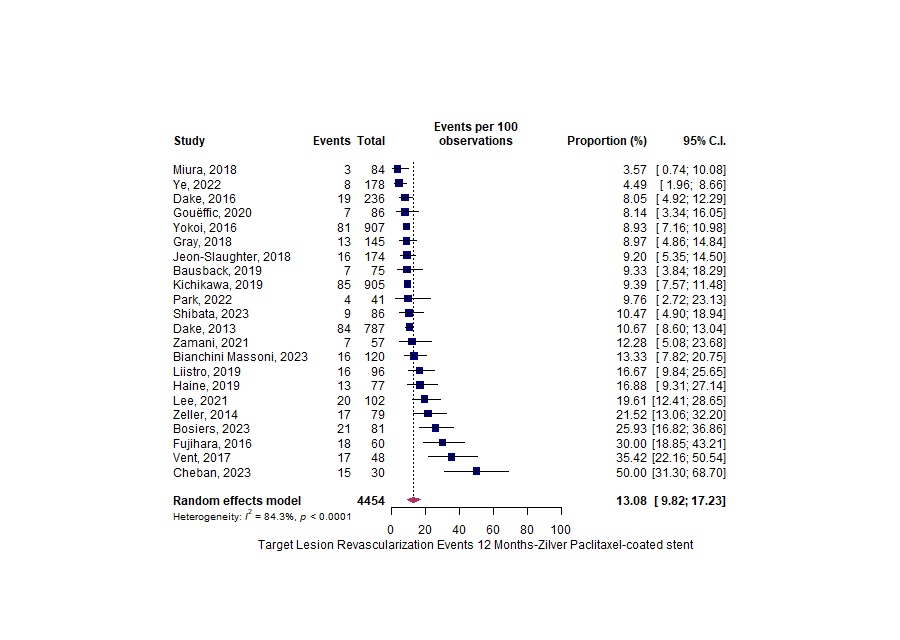


Supplementary Figure 51. Target lesion revascularisation at 12 months for Zilver® PTX® according to studies with a Downs and Black Quality Appraisal rating of fair or above.


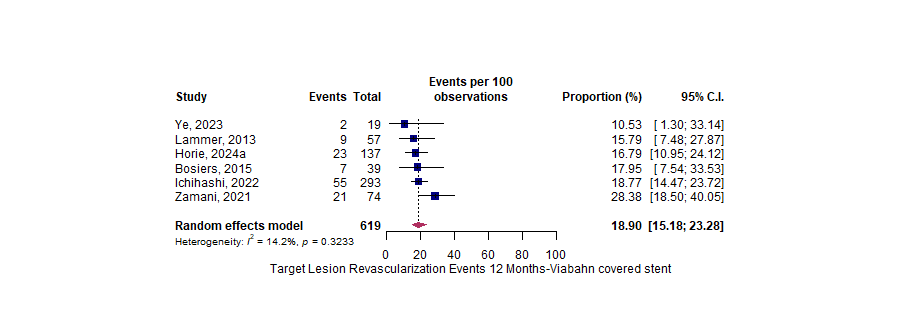


Supplementary Figure 52. Target lesion revascularisation at 12 months for Viabahn according to studies with a Downs and Black Quality Appraisal rating of fair or above.


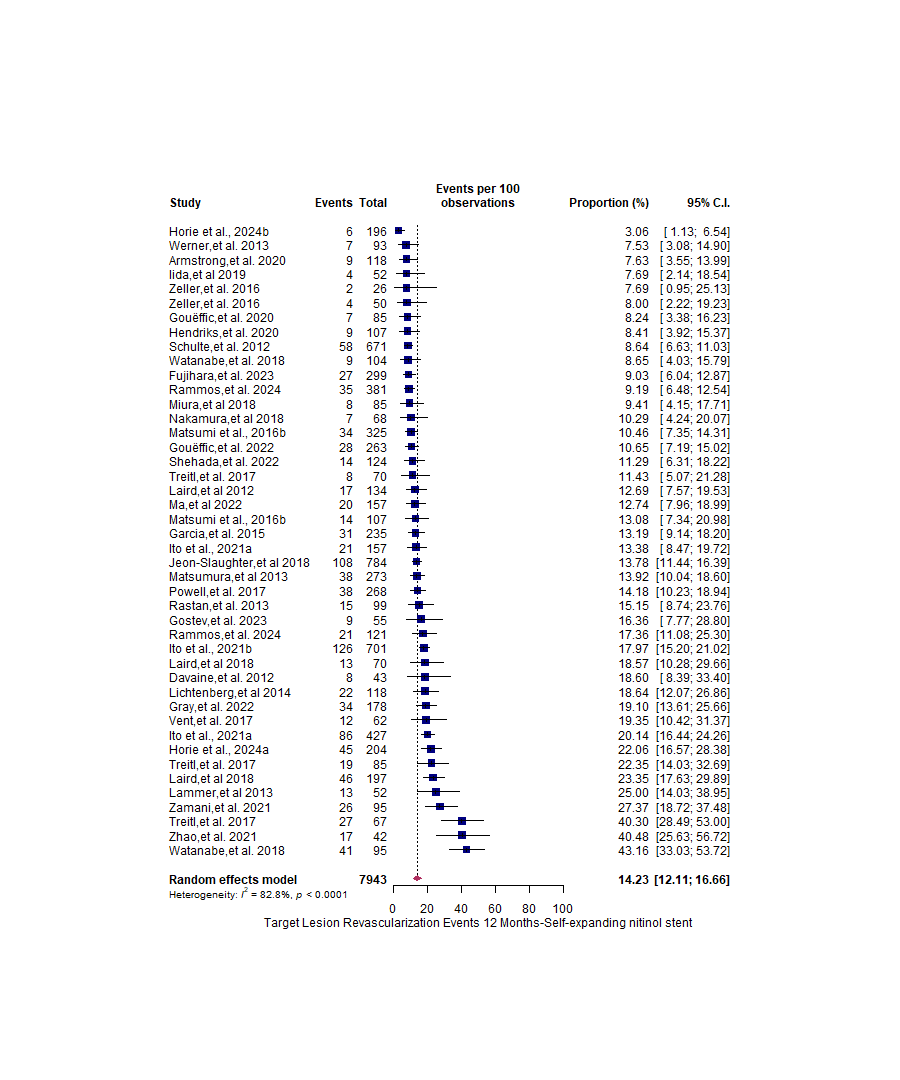


Supplementary Figure 53. Target lesion revascularisation at 12 months for BMS according to studies with a Downs and Black Quality Appraisal rating of fair or above.


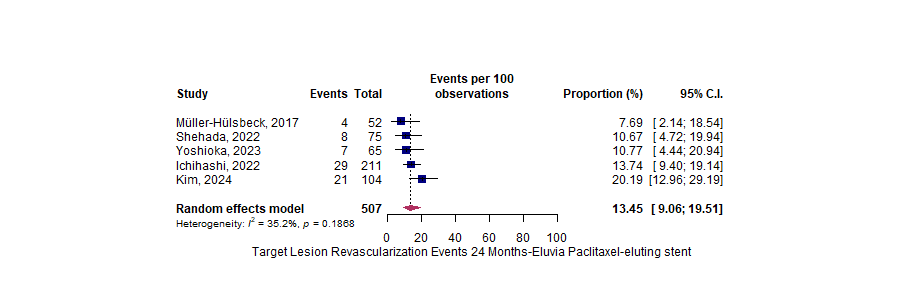


Supplementary Figure 54. Target lesion revascularisation at 24 months for Eluvia™ according to studies with a Downs and Black Quality Appraisal rating of fair or above.


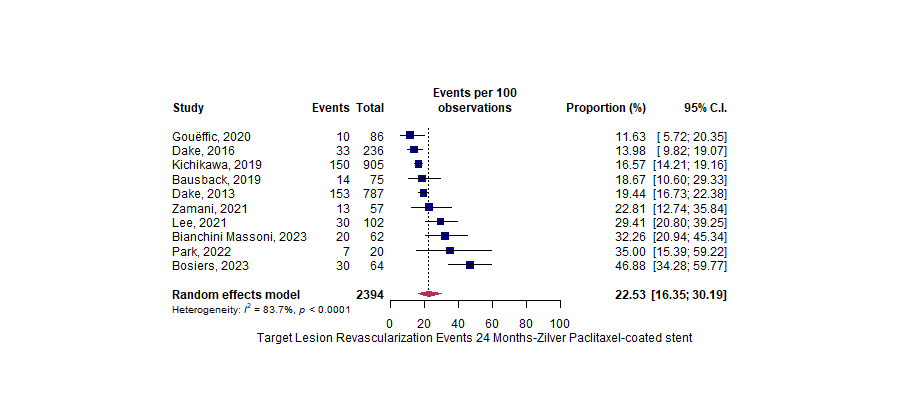


Supplementary Figure 55. Target lesion revascularisation at 24 months for Zilver® PTX® according to studies with a Downs and Black Quality Appraisal rating of fair or above.


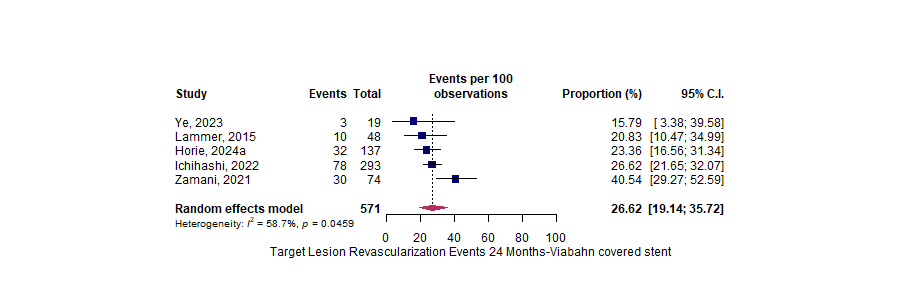


Supplementary Figure 56. Target lesion revascularisation at 24 months for Viabahn according to studies with a Downs and Black Quality Appraisal rating of fair or above.


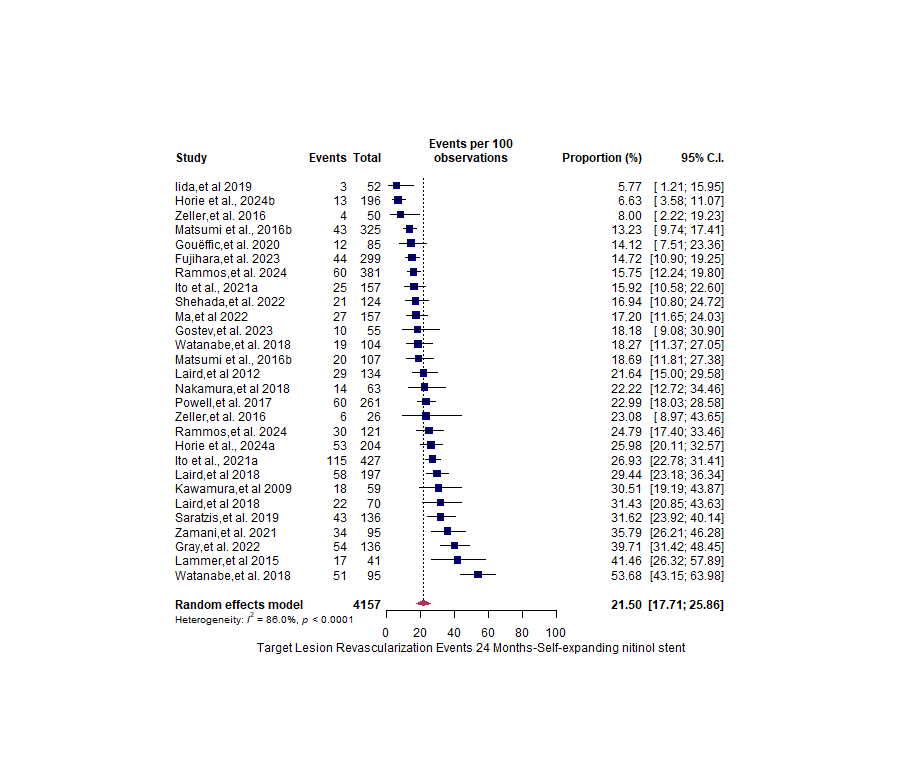


Supplementary Figure 57. Target lesion revascularisation at 24 months for BMS according to studies with a Downs and Black Quality Appraisal rating of fair or above.


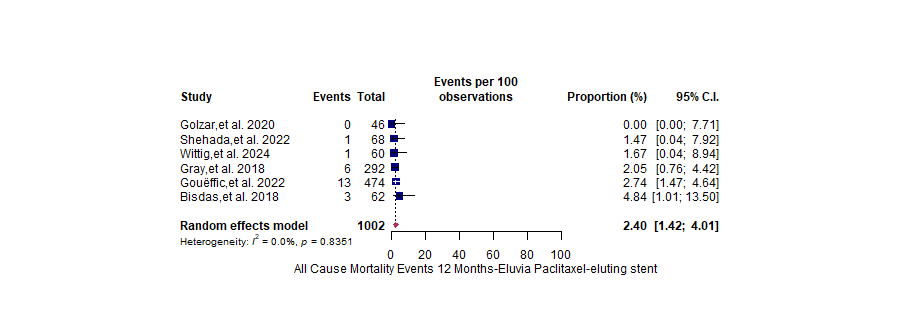


Supplementary Figure 58. Mortality at 12 months for Eluvia™.


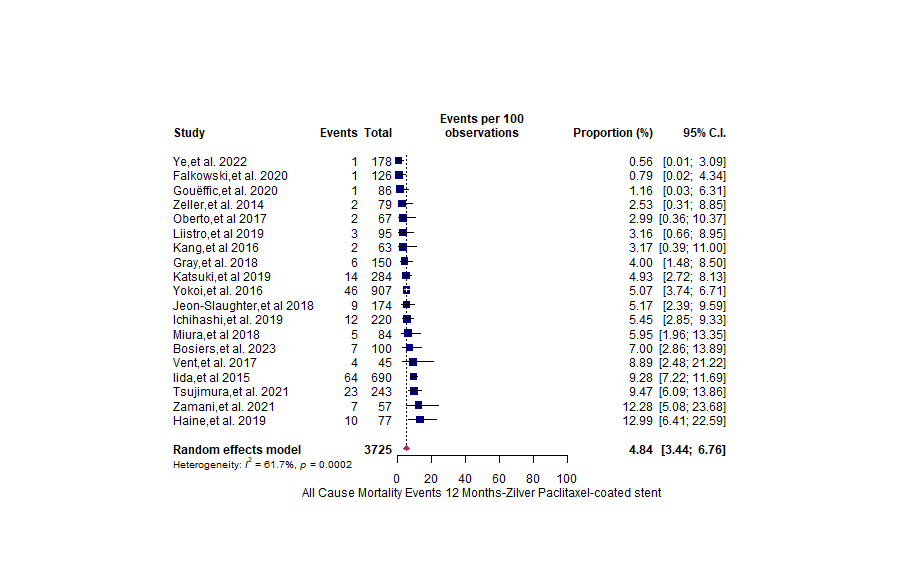


Supplementary Figure 59. Mortality at 12 months for Zilver® PTX®.


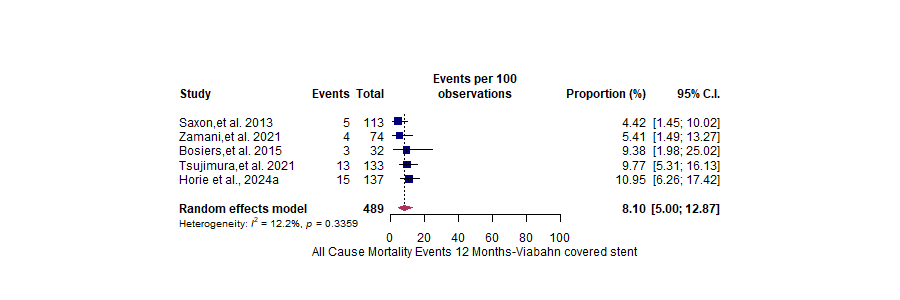


Supplementary Figure 60. Mortality at 12 months for Viabahn.


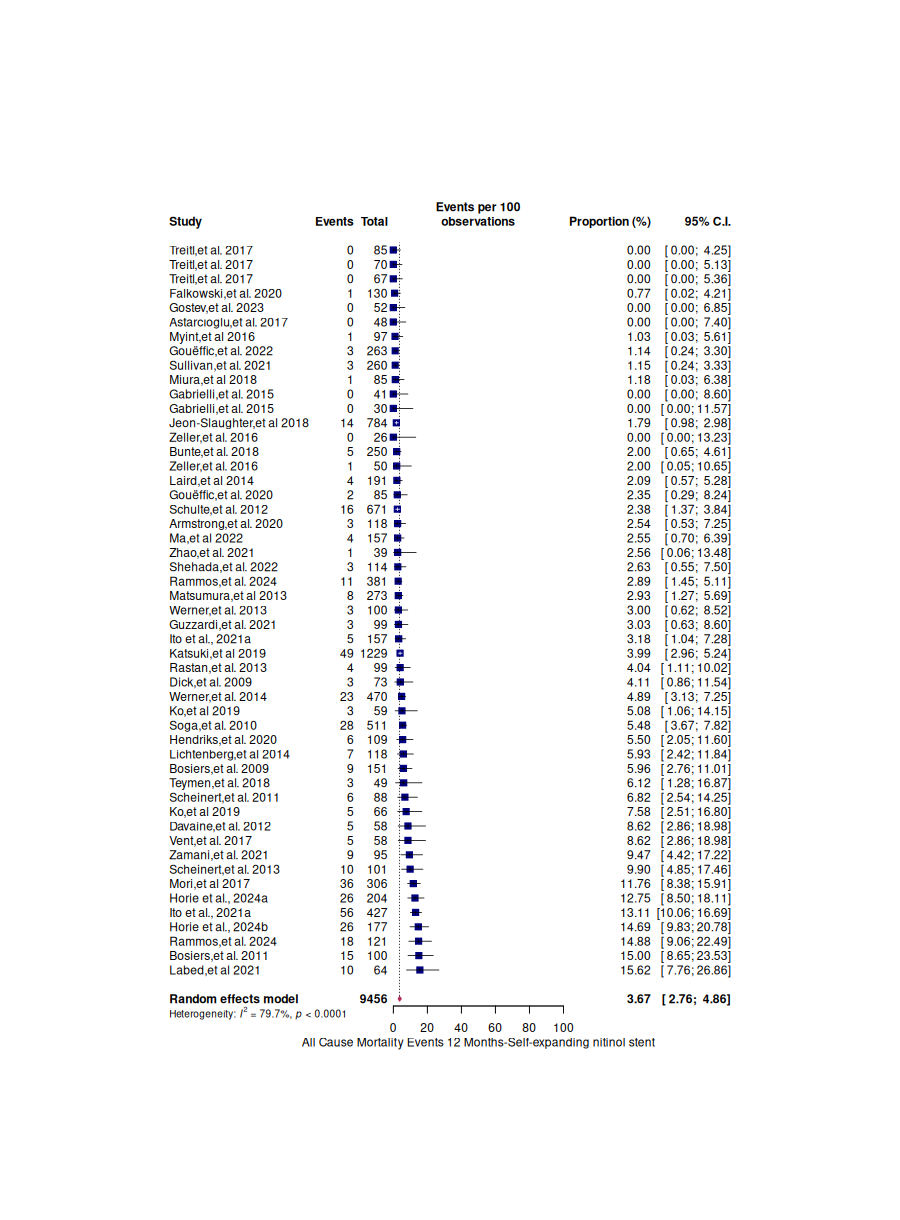


Supplementary Figure 61. Mortality at 12 months for BMS.


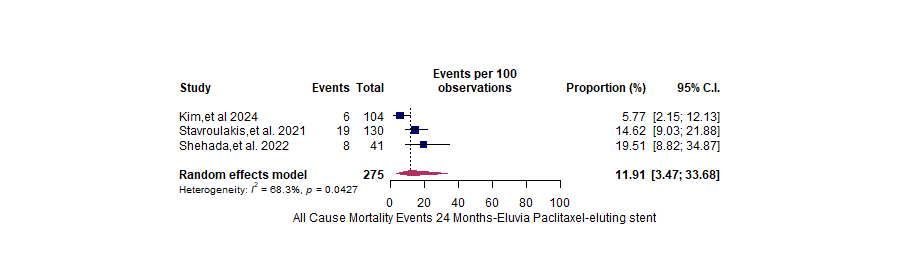


Supplementary Figure 62. Mortality at 24 months for Eluvia™.


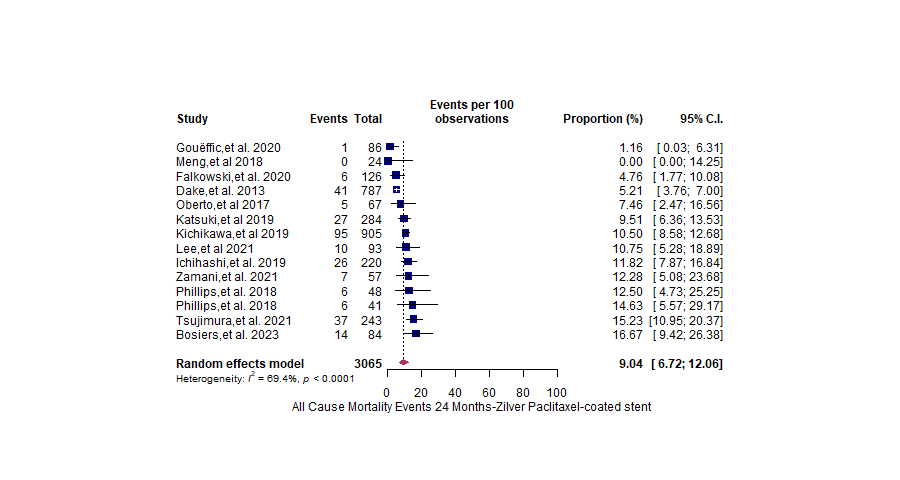


Supplementary Figure 63. Mortality at 24 months for Zilver® PTX®.


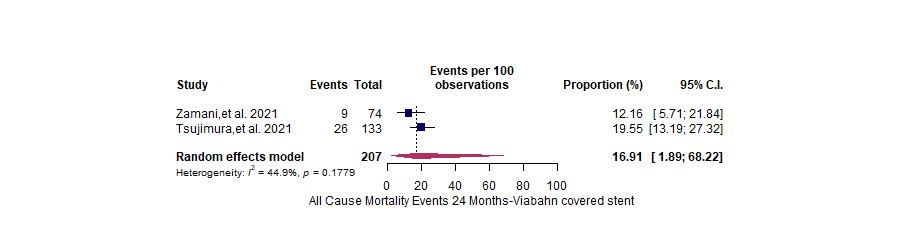


Supplementary Figure 64. Mortality at 24 months for Viabahn.


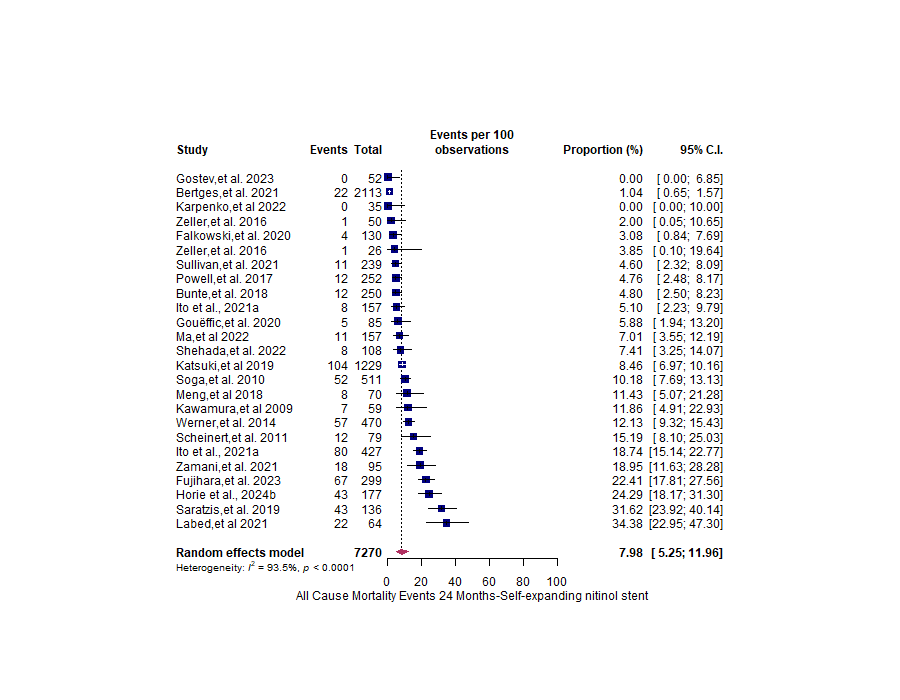


Supplementary Figure 65. Mortality at 24 months for BMS.


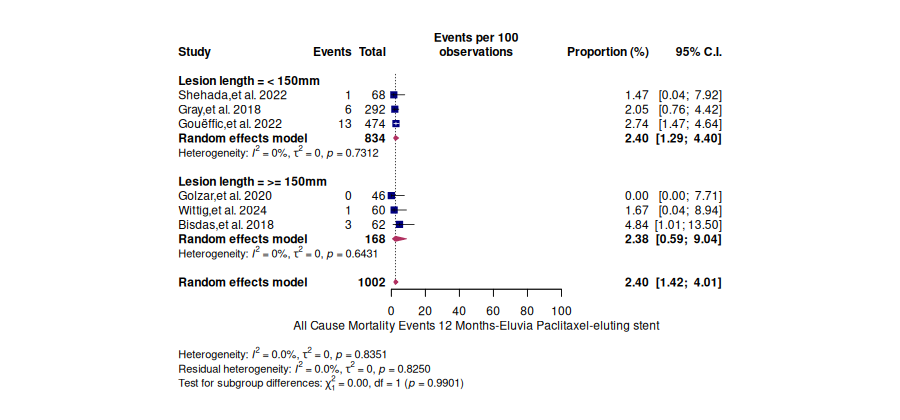


Supplementary Figure 66. Mortality for short and long lesions at 12 months for Eluvia™.


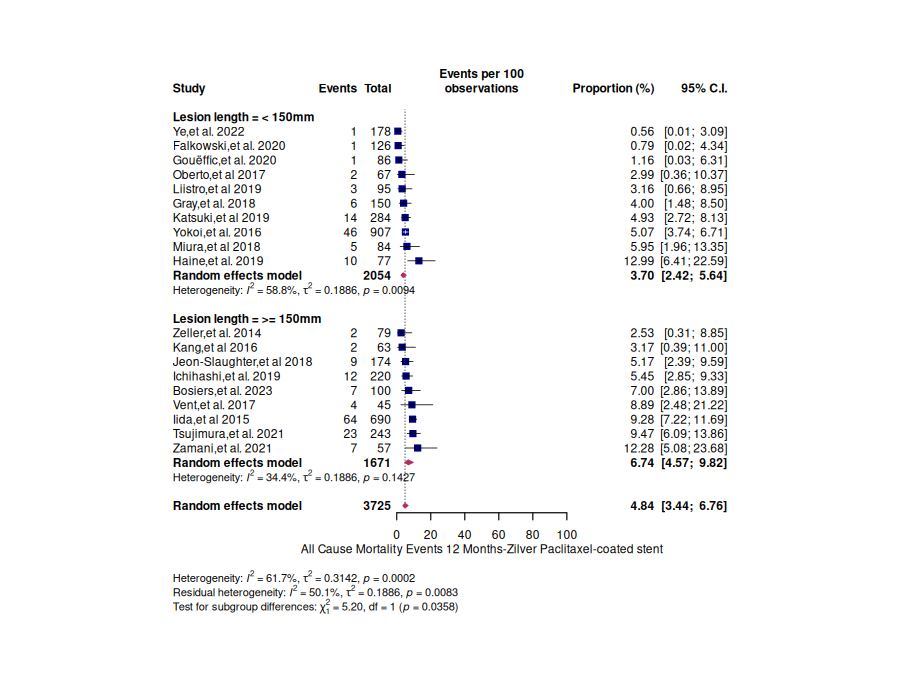


Supplementary Figure 67. Mortality for short and long lesions at 12 months for Zilver® PTX®.


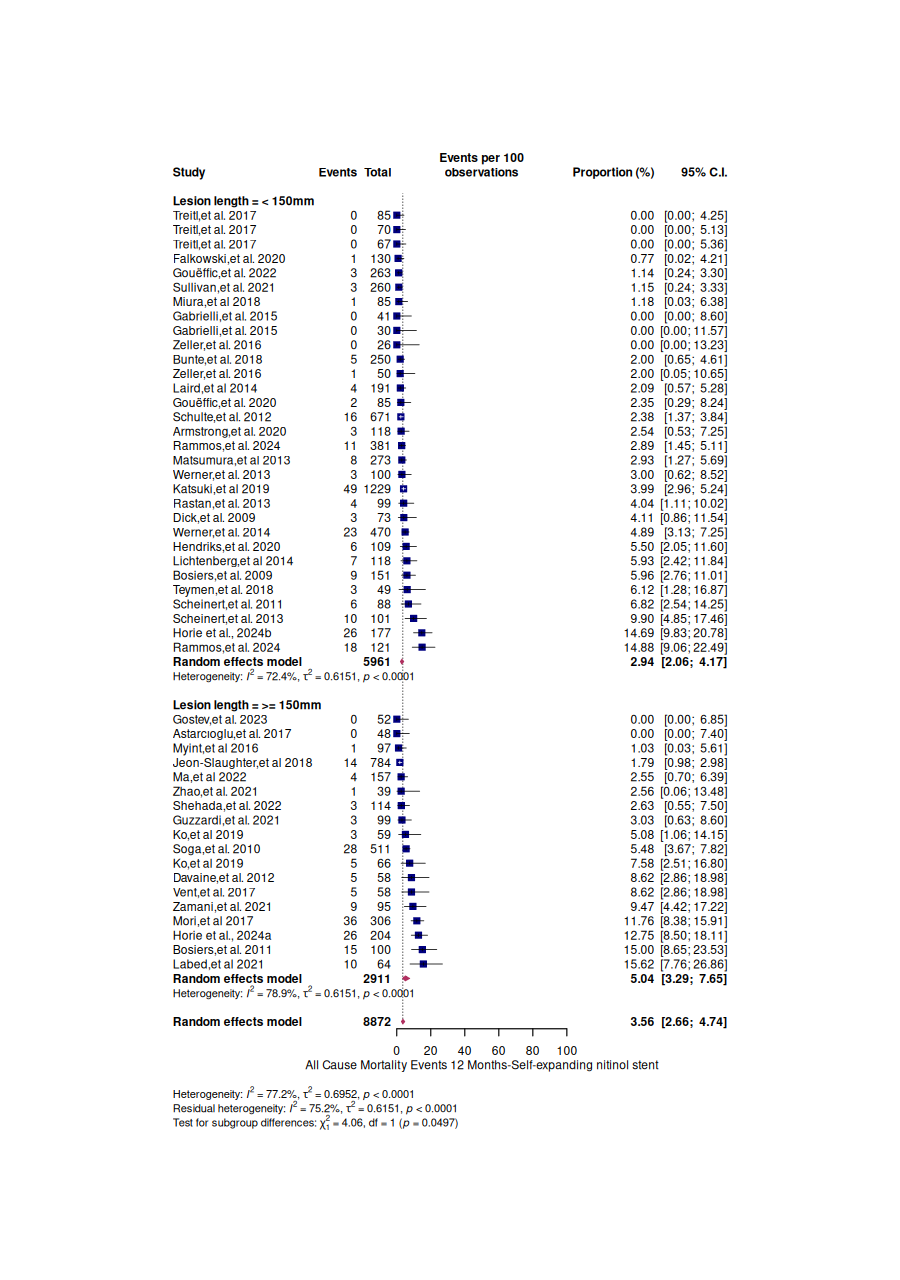


Supplementary Figure 68. Mortality for short and long lesions at 12 months for BMS.


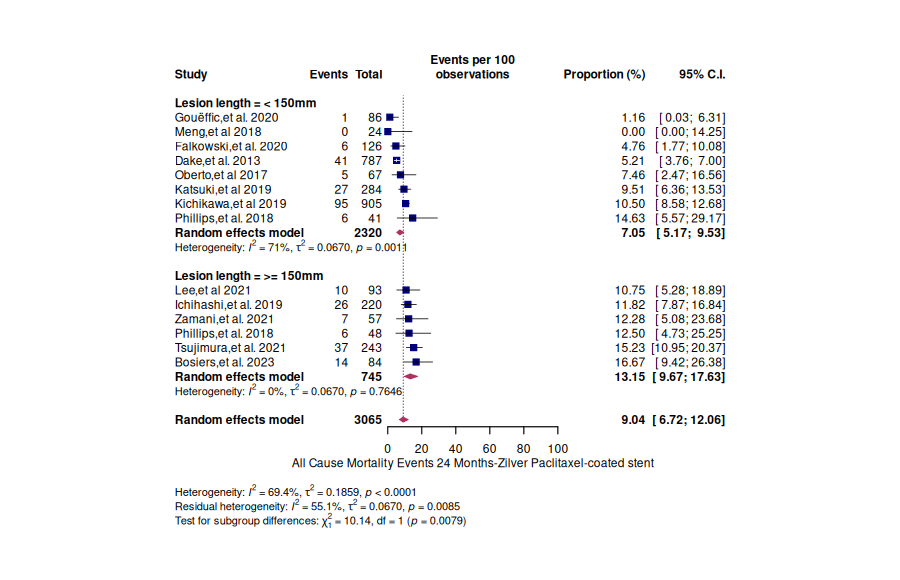


Supplementary Figure 69. Mortality for short and long lesions at 24 months for Zilver® PTX®.


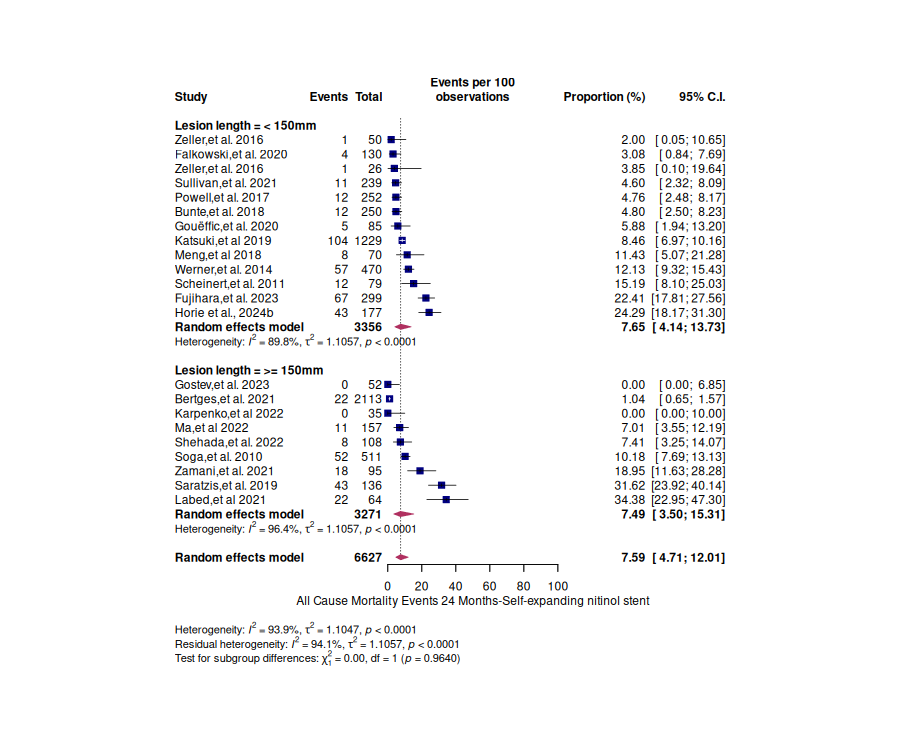


Supplementary Figure 70. Mortality for short and long lesions at 24 months for BMS


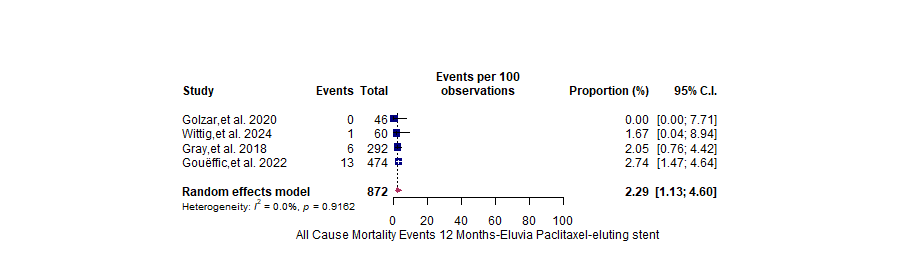


Supplementary Figure 71. Mortality at 12 months for Eluvia™ according to studies that reported using core laboratory adjudication.


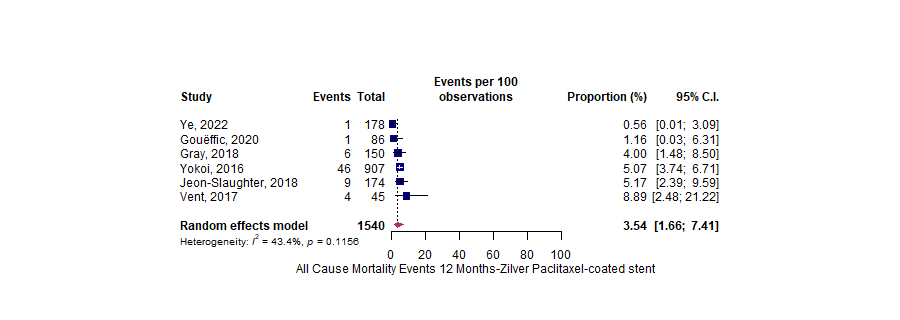


Supplementary Figure 72. Mortality at 12 months for Zilver® PTX® according to studies that reported using core laboratory adjudication.


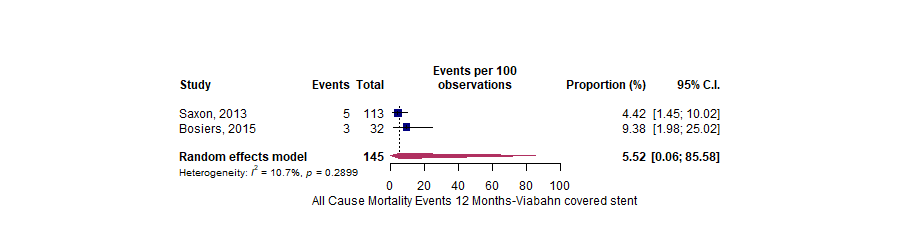


Supplementary Figure 73. Mortality at 12 months for Viabahn according to studies that reported using core laboratory adjudication.


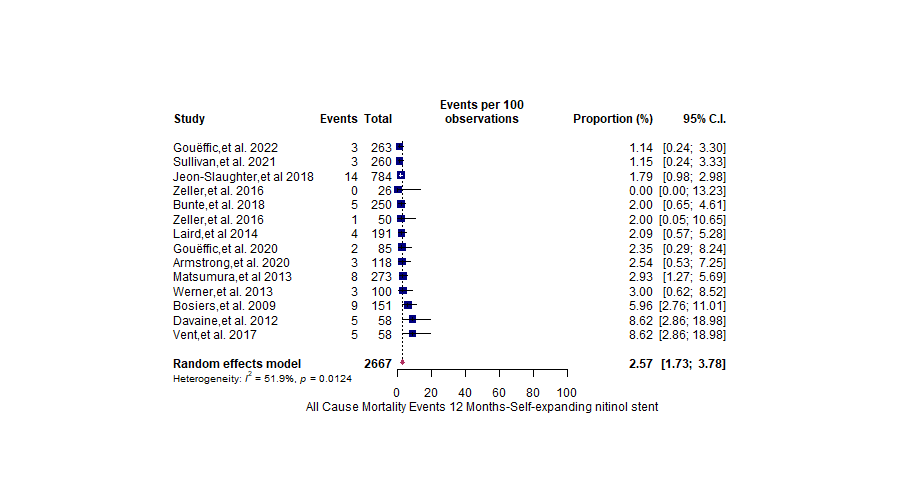


Supplementary Figure 74. Mortality at 12 months for BMS according to studies that reported using core laboratory adjudication.


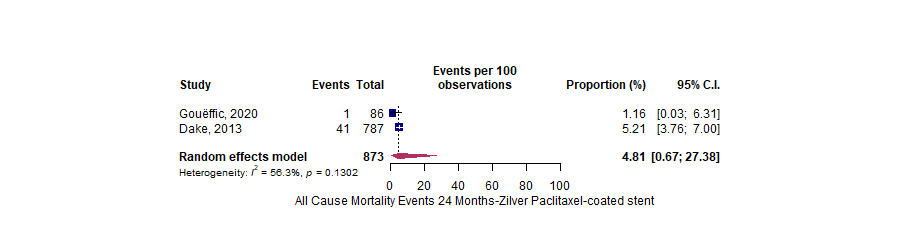


Supplementary Figure 75. Mortality at 24 months for Zilver® PTX® according to studies that reported using core laboratory adjudication.


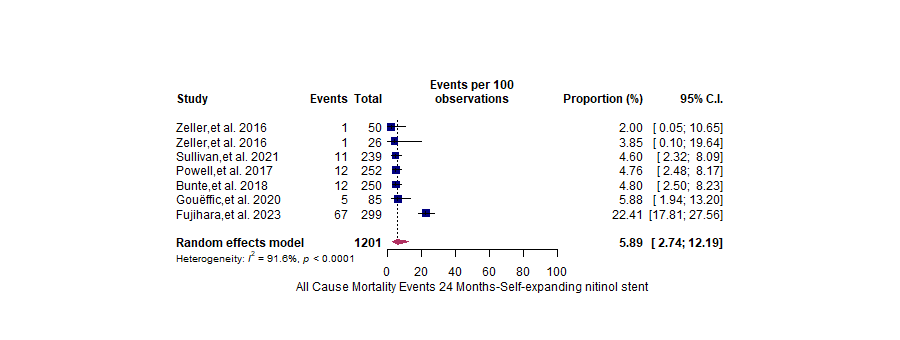


Supplementary Figure 76. Mortality at 24 months for BMS according to studies that reported using core laboratory adjudication.


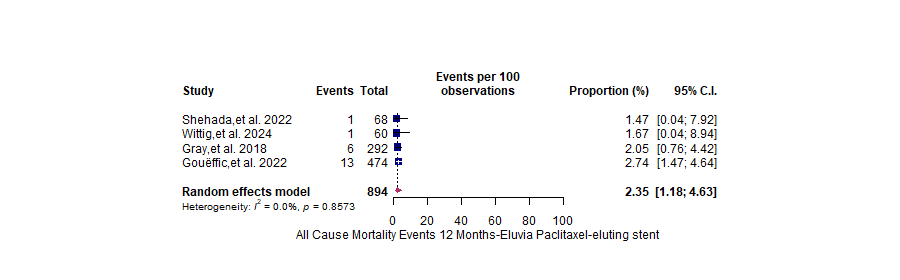


Supplementary Figure 77. Mortality at 12 months for Eluvia™ according to studies with a Downs and Black Quality Appraisal rating of fair or above.


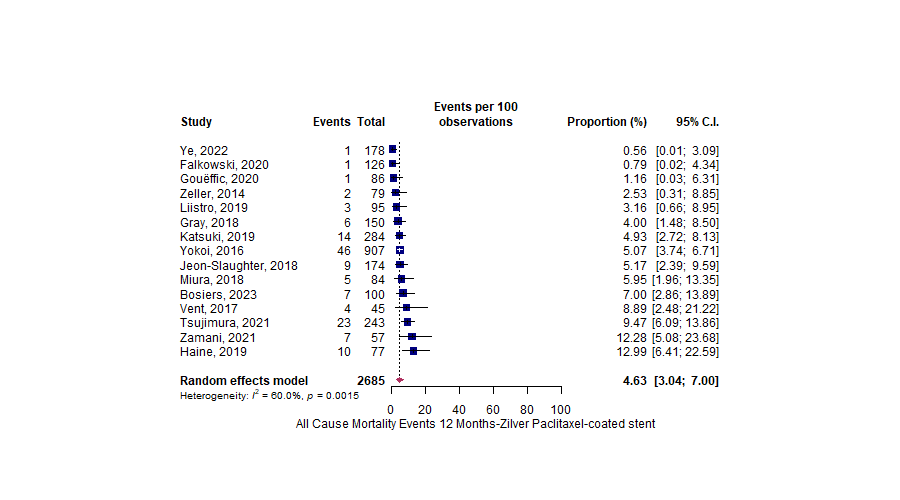


Supplementary Figure 78. Mortality at 12 months for Zilver® PTX® according to studies with a Downs and Black Quality Appraisal rating of fair or above.


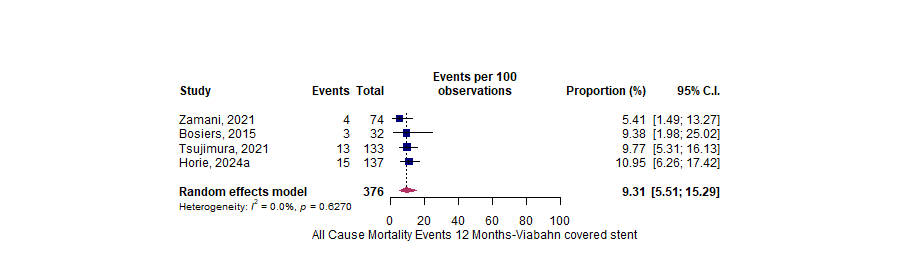


Supplementary Figure 79. Mortality at 12 months for Viabahn according to studies with a Downs and Black Quality Appraisal rating of fair or above.


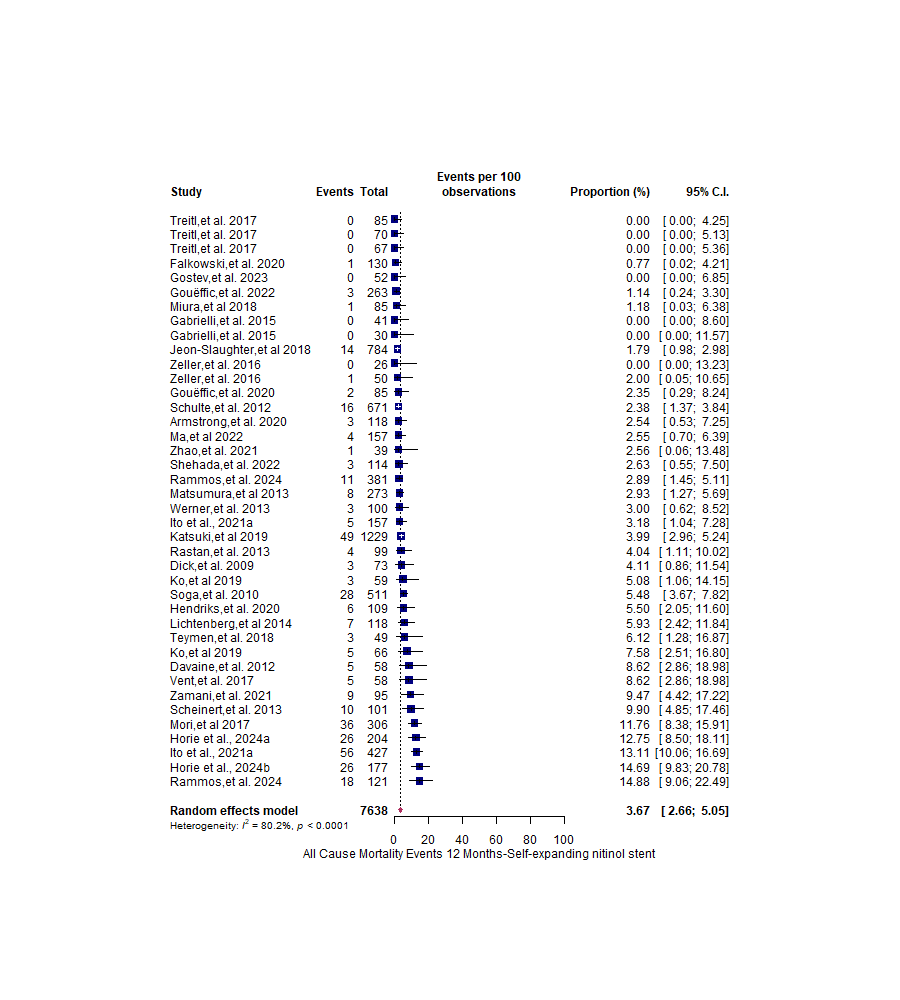


Supplementary Figure 80. Mortality at 12 months for BMS according to studies with a Downs and Black Quality Appraisal rating of fair or above.


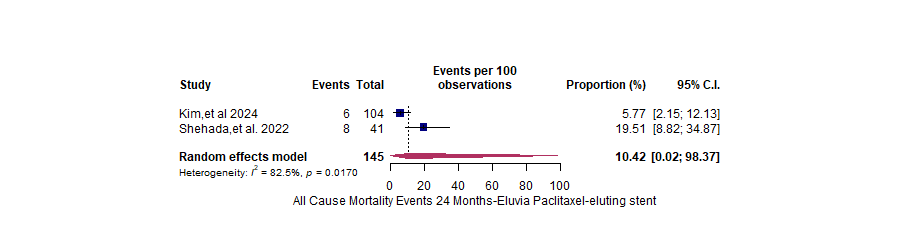


Supplementary Figure 81. Mortality at 24 months for Eluvia™ according to studies with a Downs and Black Quality Appraisal rating of fair or above.


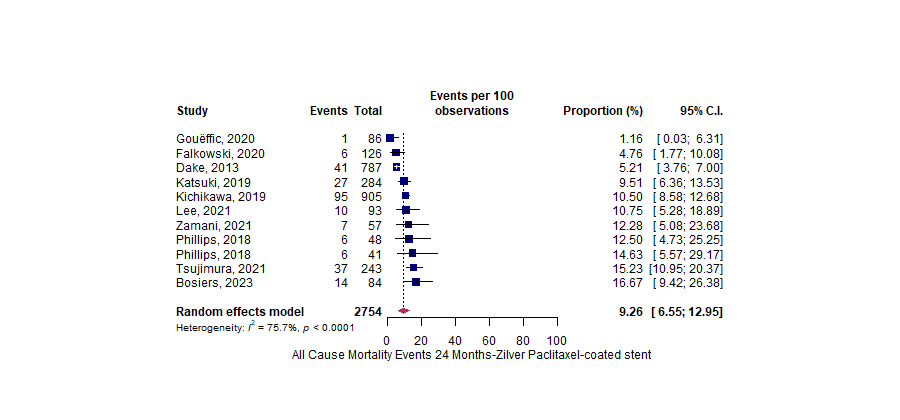


Supplementary Figure 82. Mortality at 24 months for Zilver® PTX® according to studies with a Downs and Black Quality Appraisal rating of fair or above.


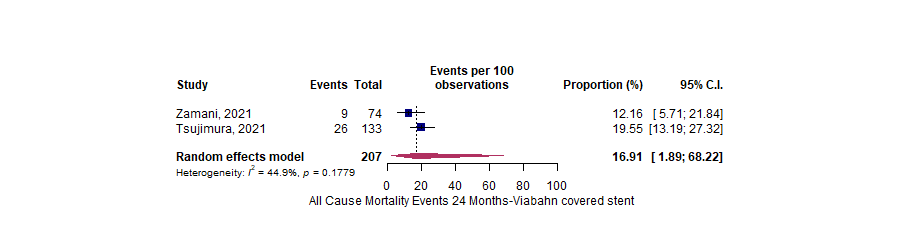


Supplementary Figure 83. Mortality at 24 months for Viabahn according to studies with a Downs and Black Quality Appraisal rating of fair or above.


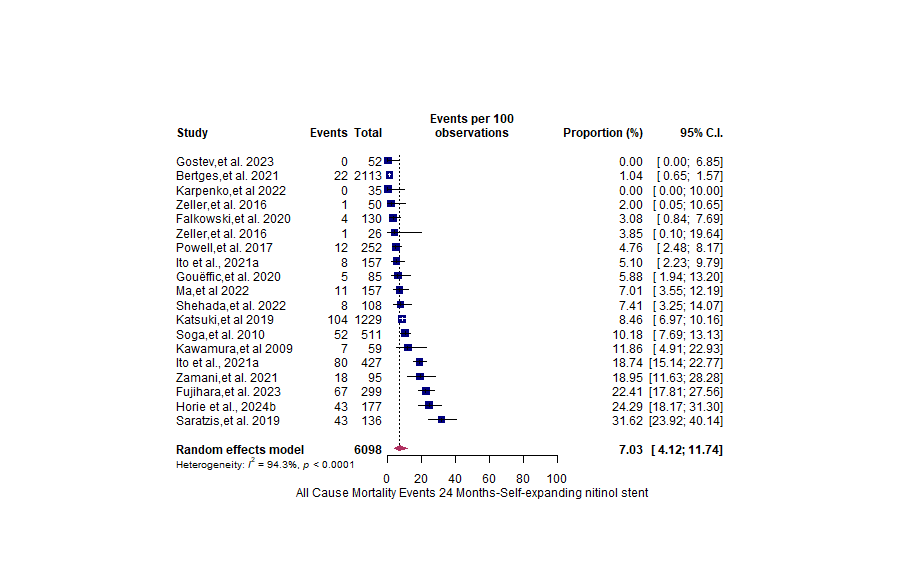


Supplementary Figure 84. Mortality at 24 months for BMS according to studies with a Downs and Black Quality Appraisal rating of fair or above.


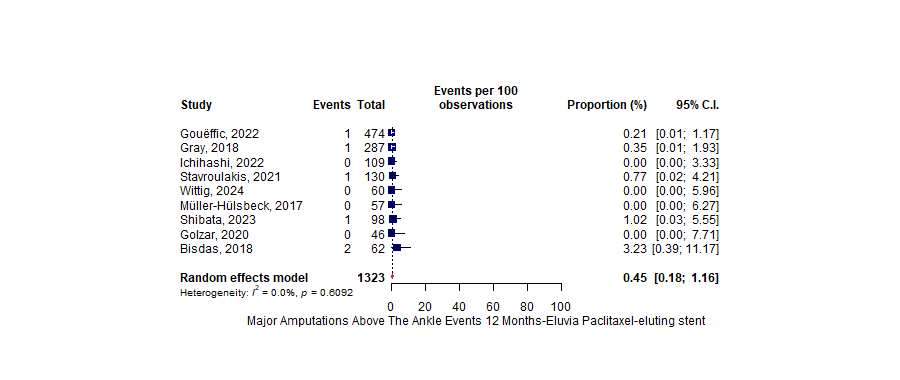


Supplementary Figure 85. Major amputations at 12 months for Eluvia™.


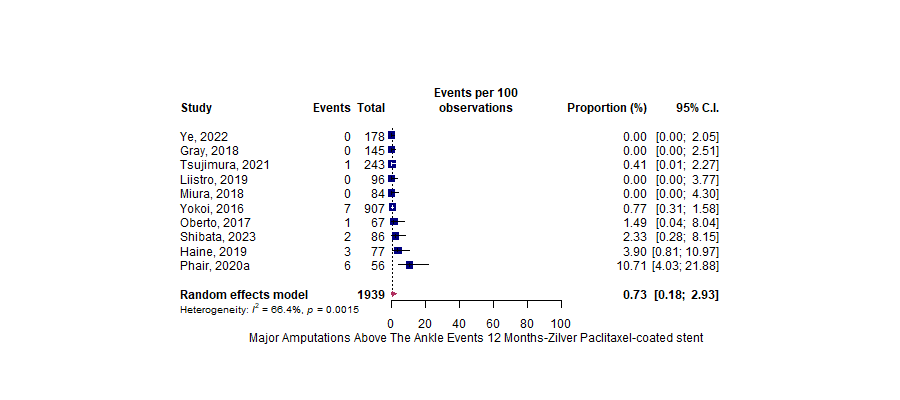


Supplementary Figure 86. Major amputations at 12 months for Zilver® PTX®.


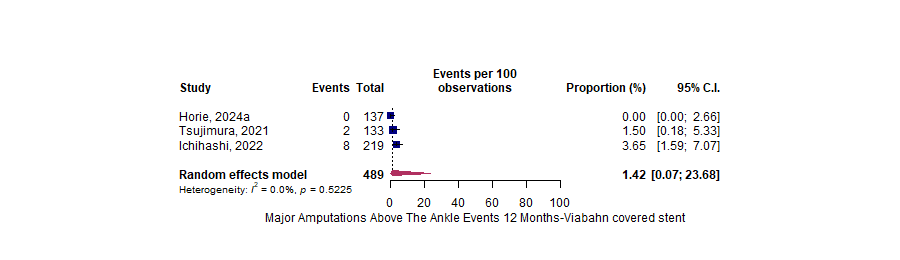


Supplementary Figure 87. Major amputations at 12 months for Viabahn®.


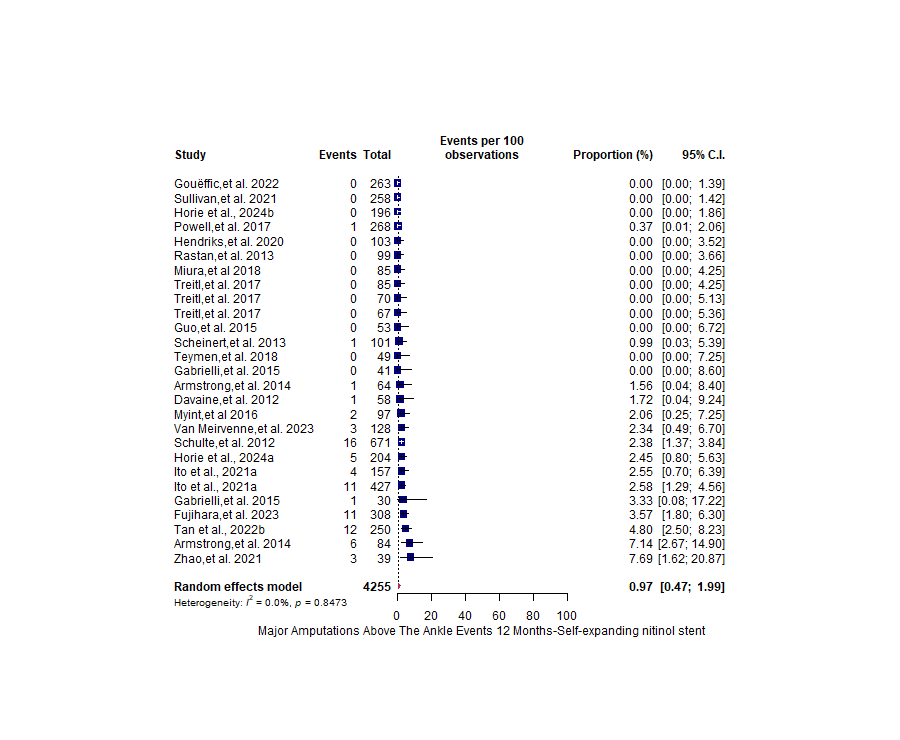


Supplementary Figure 88. Major amputations at 12 months for BMS.


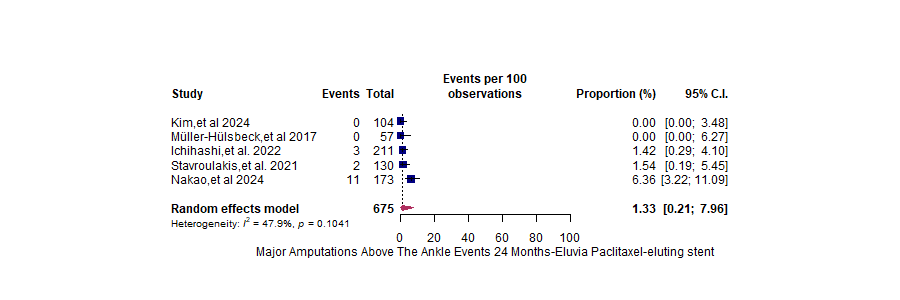


Supplementary Figure 89. Major amputations at 24 months for Eluvia™.


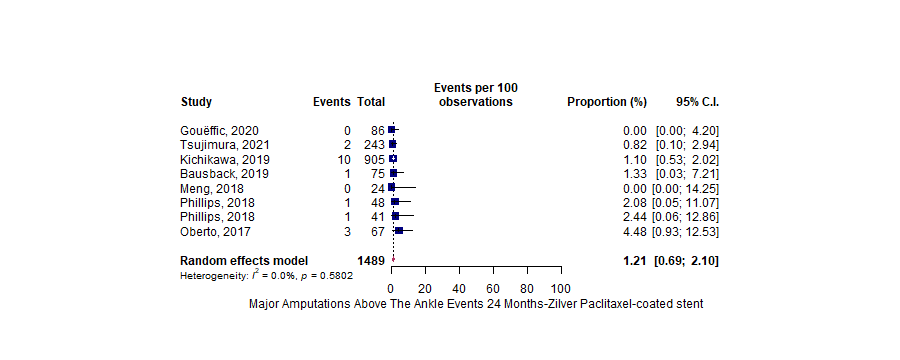


Supplementary Figure 90. Major amputations at 24 months for Zilver® PTX®.


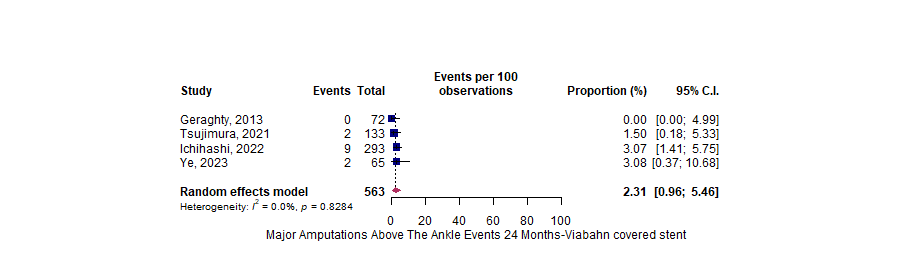


Supplementary Figure 91. Major amputations at 24 months for Viabahn.


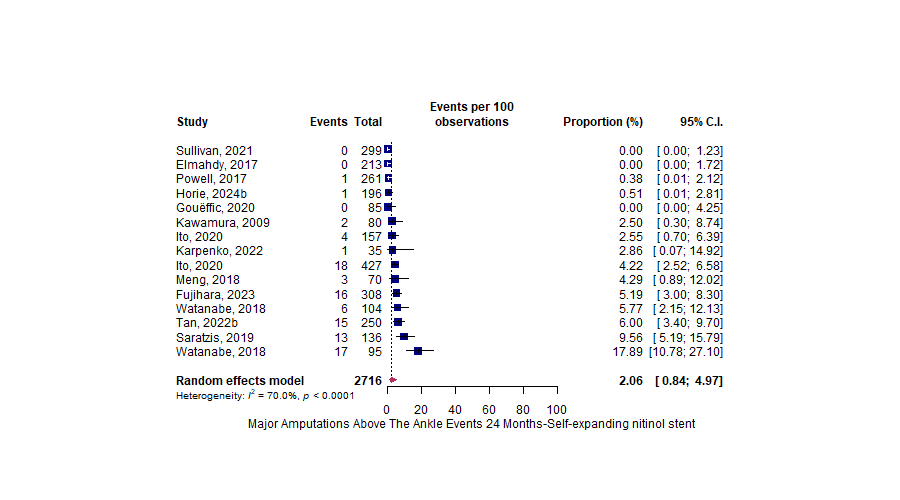


Supplementary Figure 92. Major amputations at 24 months for BMS.


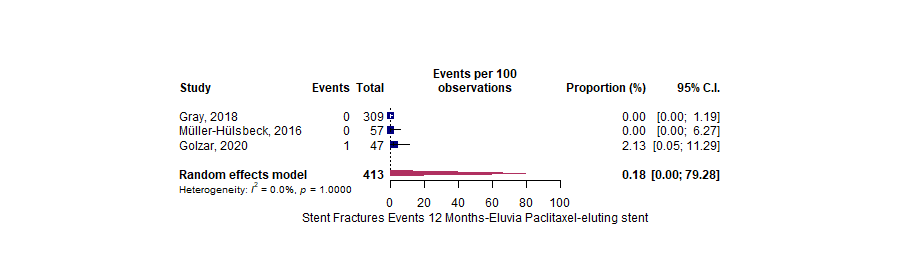


Supplementary Figure 93. Stent fractures at 12 months for Eluvia™.


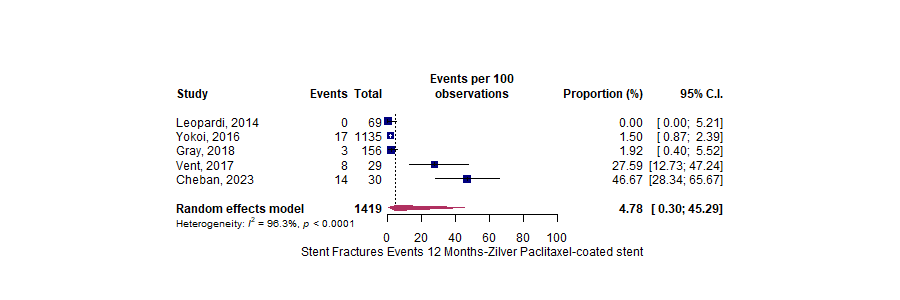


Supplementary Figure 94. Stent fractures at 12 months for Zilver® PTX®.


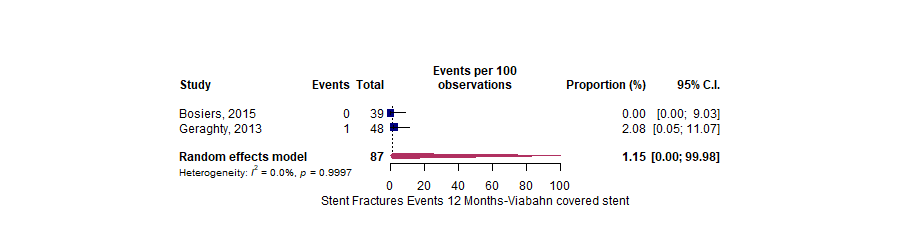


Supplementary Figure 95. Stent fractures at 12 months for Viabahn.


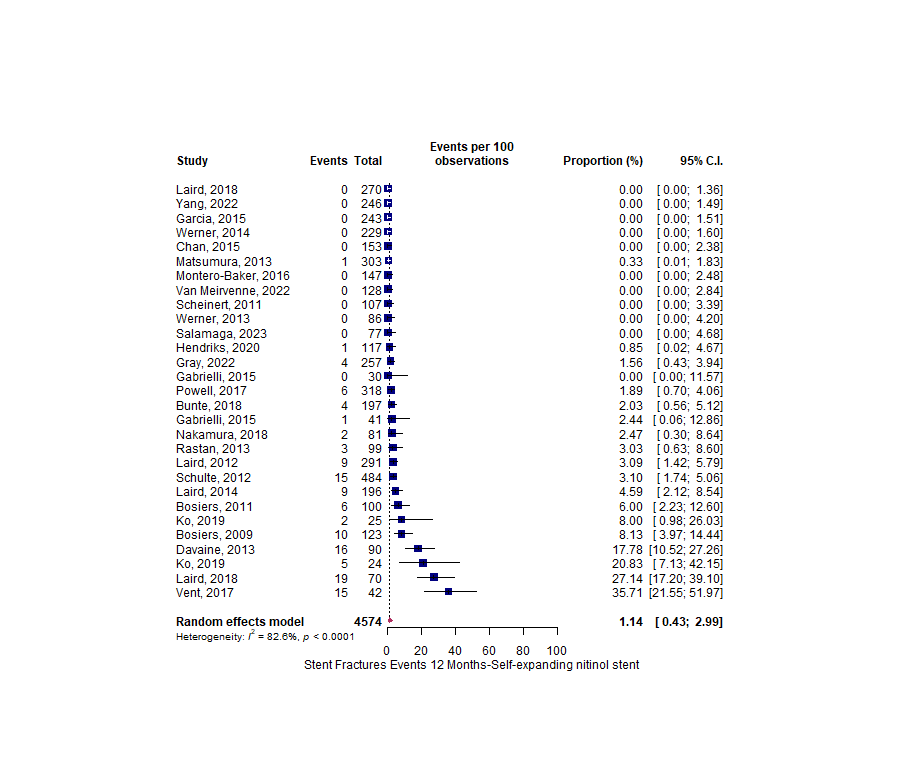


Supplementary Figure 96. Stent fractures at 12 months for BMS.


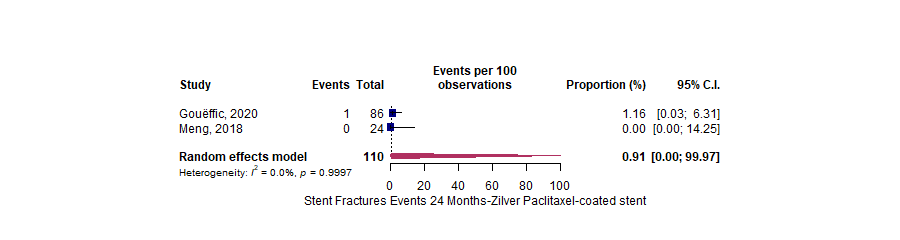


Supplementary Figure 97. Stent fractures at 24 months for Zilver® PTX®.


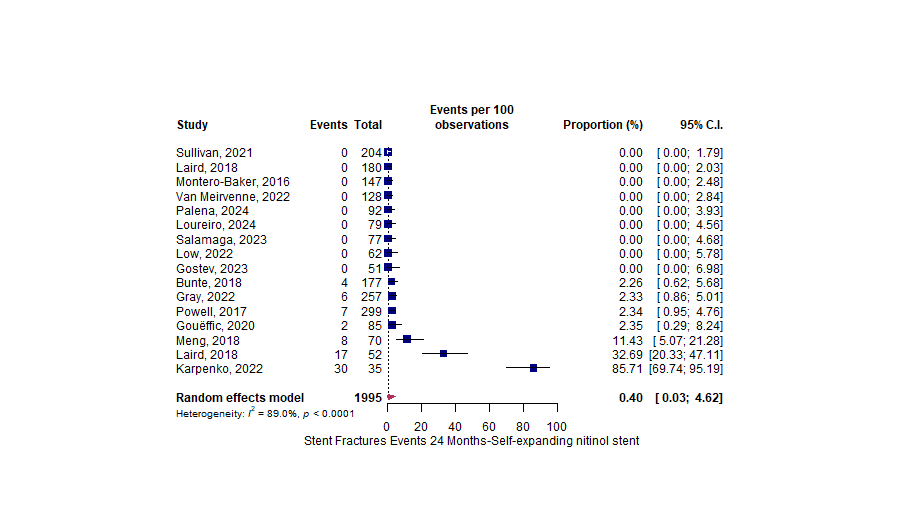


Supplementary Figure 98. Stent fractures at 24 months for BMS.


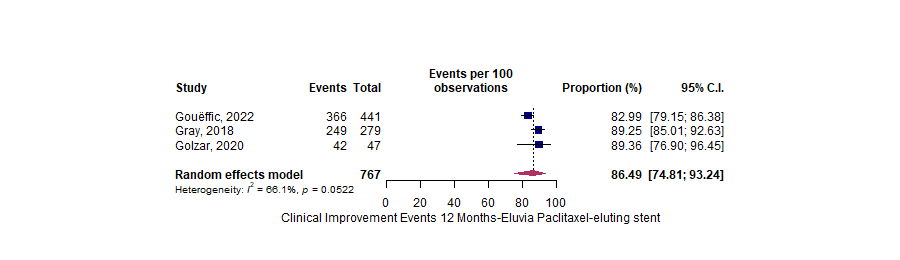


Supplementary Figure 99. Clinical improvement at 12 months for Eluvia™.


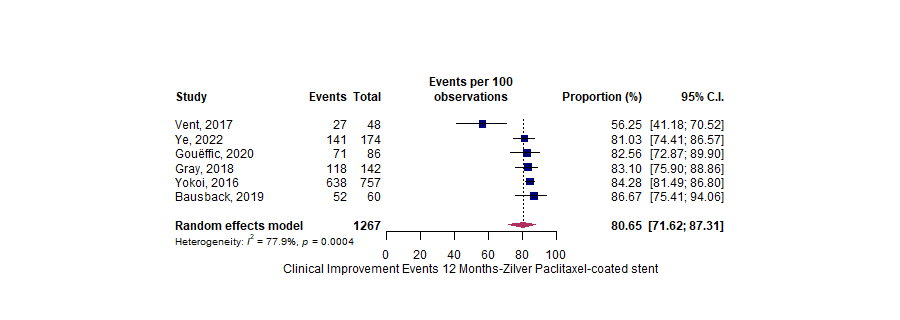


Supplementary Figure 100. Clinical improvement at 12 months for Zilver® PTX®.

Supplementary Figure 101. Clinical improvement at 12 months for BMS.
